# Efficient perovskite LEDs with tailored atomic layer number emission at fixed wavelengths

**DOI:** 10.1126/sciadv.adp9595

**Published:** 2025-02-14

**Authors:** Ligang Wang, Zher Ying Ooi, Feng-Yan Jia, Yuqi Sun, Yun Liu, Linjie Dai, Junzhi Ye, Jincan Zhang, Hio-Ieng Un, Yu-Hsien Chiang, Sanyang Han, Alessandro James Mirabelli, Miguel Anaya, Zhilong Zhang, Yang Lu, Chen Zou, Baodan Zhao, Dawei Di, Xiaodong Yang, Dengyang Guo, Yu Tan, Hao Dong, Shaocheng Liu, Tianjun Liu, Huanping Zhou, Samuel D. Stranks, Ling-Dong Sun, Chun-Hua Yan, Richard H. Friend

**Affiliations:** ^1^ School of Materials Science and Engineering, Peking University, Beijing 100871, P. R. China.; ^2^ Cavendish Laboratory, University of Cambridge, JJ Thomson Avenue, Cambridge CB3 0HE, UK.; ^3^ Beijing National Laboratory for Molecular Sciences, State Key Laboratory of Rare Earth Materials Chemistry and Applications, PKU-HKU Joint Laboratory in Rare Earth Materials and Bioinorganic Chemistry, College of Chemistry and Molecular Engineering, Peking University, Beijing 100871, P. R. China.; ^4^ Department of Applied Physics, Royal Institute of Technology, Albanova University Centre, 106 91 Stockholm, Sweden.; ^5^ Department of Chemical Engineering and Biotechnology, University of Cambridge, Cambridge CB3 0AS, UK.; ^6^ Institute of High Performance Computing (IHPC), Agency for Science, Technology and Research (A*STAR), 1 Fusionopolis Way, #16-16 Connexis, Singapore 138632, Republic of Singapore.; ^7^ Department of Engineering, University of Cambridge, Cambridge CB3 0FA, UK.; ^8^ State Key Laboratory of Extreme Photonics and Instrumentation, College of Optical Science and Engineering; International Research Center for Advanced Photonics, Zhejiang University, Hangzhou, P. R. China.; ^9^ Hubei Key Laboratory of Processing and Application of Catalytic Materials, College of Chemical Engineering, Huanggang Normal University, Huanggang, Hubei, P. R. China.

## Abstract

Colloidal quantum dots (QDs) have illuminated computer monitors and television screens due to their fascinating color-tunable properties depending on the size. Here, the electroluminescence (EL) wavelength of perovskite LEDs was tuned via the atomic layer number (ALN) of nanoplates (NPs) instead of the “size” in conventional QDs. We demonstrated efficient LEDs with controllably tailored emission from *n* = 3, 4, 5, and ≥7 ALN perovskite NPs with specific and discrete major peaks at 607, 638, 669, and 728 nanometers. These LEDs demonstrated peak external quantum efficiency (EQE) of 26.8% and high wavelength reproducibility with less than 1 to 2 nm difference between batches. High color stability without observable EL spectral change and operating stability with the best *T*_50_ of 267 minutes at 1.0 milliampere per square centimeter was also achieved. This work demonstrates a concept of tailoring specific ALN emission with fixed wavelengths, shedding light on efficient, emission-discrete, and color-stable LEDs for next-generation display.

## Teaser

Perovskite LEDs with tailored atomic layer number emission at fixed wavelengths show promise for next-generation display.

## INTRODUCTION

Quantum dot (QDs) are excellent light emitters for high-definition displays since the first demonstration in the early 1980s with the Chemistry Nobel Prize in 2023 awarded to the first pioneers (*1*–*3*). Ultrahigh quantum efficiency, simplicity of synthesis, and easy tunability of optical properties render perovskite nanoplates (NPs) promising materials for light-emitting diodes (LEDs) since the first demonstration of perovskite LEDs in 2014 (*4*). Extensive efforts have produced state-of-the-art perovskite NP LEDs with more than 20% external quantum efficiency (EQE) both in green and red emission ranges (table S1) (*5*–*29*). The wider tunability of wavelength and sharper emission provide a wider color gamut to restore natural colors and make the LED screens more vivid. It can also offer possibilities to meet the standards for different purposes and achieve higher photopic luminance due to the photopic sensitivity of humans reaching the peak at 555 nm (fig. S1).

The strategy of tuning the color of conventional QDs was achieved via size control which relies on synthesis conditions such as precursor composition, reaction temperature/time, and ligand type/ratio (*23*, *30*–*32*). Furthermore, the mixed-halide strategy has been widely used for emission wavelength tuning in perovskite LEDs (*10*, *33*–*36*), and this strategy can provide large and continuous emission wavelength tunability covering from deep blue to infrared range (*8*, *37*, *38*). However, mixed halide perovskite materials always suffer from color instabilities due to halide segregation under photoexcitation or electroluminescent operation (*10*, *39*–*41*).

Although it is widely known that the bandgap/emission wavelength changes as layer of QDs increases, to the best of our knowledge, there are still no available reports demonstrating effective methods for tailoring electroluminescence (EL) from emitters with specific integer layers of atoms and achieving high efficiency at the same time in optoelectronic field (*42*–*44*). Differing from size control in conventional QDs and the mixed halide in perovskite strategies demonstrating continuous wavelength turnability, the emission of these NP LEDs relies on the number of atomic unit cell monolayers [PbX_4_] (also defined as *n*-phase). This feature makes the wavelength of emission specific and discrete, similar to discrete energy levels in atoms. Moreover, the fixed feature can make the emission wavelength of LEDs highly reproducible due to the wavelength relying on the atomic layer number (ALN) rather than size or composition which is more susceptible to preparation conditions. Furthermore, Förster resonance energy transfer (FRET) and charge transfer (CT) are generally proposed to be responsible for the energy funneling in multiphase perovskite LEDs (*45*–*47*). However, the exact mechanism and the difference of energy funneling in PL and EL, which are important for reasonable design of LEDs, are still strongly debated and remain to be revealed, especially in the NP system which can be different from the conventional bulk quasi–two-dimensional (2D) perovskites.

## RESULTS

### LEDs with tailored ALN emission

**Fig. 1.**
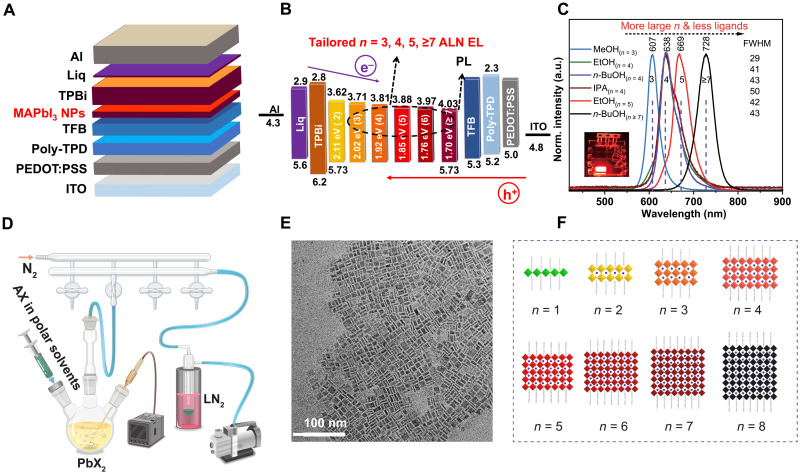
MAPbI_3_ NP LEDs with different ALN emission. (**A**) MAPbI_3_ NP LEDs with structure: ITO/PEDOT:PSS/poly-TPD/TFB/MAPbI_3_ NPs (~10 nm)/TPBi/Liq/Al. ITO, indium tin oxide; PEDOT:PSS, poly(3,4-ethylenedioxythiophene) polystyrene sulfonate; poly-TPD, poly[*N*,*N*′-bis(4-butylphenyl)-*N*,*N*′-bis(phenyl)-benzidine]; TFB, poly[(9,9-dioctylfluorenyl-2,7-diyl)-co-(4,4′-(*N*-(p-butylphenyl))diphenylamine)]; TPBi, 2,2′,2″-(1,3,5-benzinetriyl)-tris(1-phenyl-1-H-benzimidazole); Liq, lithium 8-hydroxyquinolinolate. (**B**) Energy band schematic of LEDs with *n* = 3, 4, 5, and ≥7 ALN emission. (**C**) EL spectra of LEDs with tailored *n* = 3, 4, 5, and ≥7 ALN emission; NPs were prepared by MeOH for *n* = 3 [marked as MeOH_(*n* = 3)_], EtOH, *n*-BuOH, and IPA for *n* = 4 [marked as EtOH_(*n* = 4)_, *n*-BuOH_(*n* = 4)_, and IPA_(*n* = 4)_], EtOH with further washing and less ligands for *n* = 5 [marked as EtOH_(*n* = 5)_], and *n*-BuOH with further washing and less ligands for *n* ≥ 7 [marked as *n*-BuOH_(*n* ≥ 7)_] emission, correspondingly. The inserted image is the working MAPbI_3_ NP LED prepared by EtOH_(*n* = 5)_ at 6 V. (**D**) Schematic of the setup and synthesis process of APbX_3_ (e.g., MAPbI_3_) NPs via the FEPS method, LN_2_, and liquid N_2_. The polarity of solvents is defined by relative polarity to water (1.000), and the relative polarity of alcohols used is MeOH (0.762) > EtOH (0.654) > *n*-BuOH (0.586) > IPA (0.546) (*67*). Detailed data are in table S4. (**E**) Transmission electron microscopy (TEM) image of MAPbI_3_ NPs prepared by *n*-BuOH. (**F**) Schematic of crystal structures of *n* = 1 to 8 MAPbI_3_ NPs terminated with protonated oleylamine, C_18_H_35_NH_3_^+^ (OM^+^), and deprotonated oleic acid, C_18_H_35_COO^−^(OA^−^). a.u., arbitrary units.

Discrete *n* = 3, 4, 5, and ≥7 ALN emission with peaks at ~607, 638, 669, and 728 nm was achieved in MAPbI_3_ [methylammonium (MA)] NP-based LEDs. The major EL peaks of these LEDs are highly fixed and demonstrate high reproducibility with ~1- to 2-nm difference from the central wavelength between batches (Fig. 1, A to C; figs. S2 and S3; and Table 1). The *n* = 4 LEDs could be prepared by different alcohols and demonstrated extensive preparation condition adaptability. To be notable, EL emission with inter-ALN major peaks has never been observed in these LEDs. In contrast, the EL peak of the reference conventional bulk quasi-2D PEA_2_MA_2_Pb_3_I_10_ [phenethylammonium (PEA)] is not specific/fixed and varies between *n* = 6 and 7 within 686 and 724 nm in different batches even prepared from the same composition precursor solutions (fig. S3F). Moreover, these NP LEDs demonstrate sharp EL with full width at half maximum (FWHM) of 29 to 43 nm, which is sharper than the conventional quasi-2D perovskite LEDs (61 nm). These MAPbI_3_ NPs were prepared by a newly proposed flash-evaporating polar solvent (FEPS) method, and the corresponding NP films in these LEDs are optimized to ~10-nm thick (fig. S4) with a roughness of 2.48 to 3.54 nm (figs. S5 and S6), while the reference bulk quasi-2D film is ~30-nm thick with a roughness of 3.03 nm (fig. S7).

**Table 1.** Performance parameters and stability test results of MAPbI_3_ NP LEDs. The numbers of LEDs for EQE_aver._ are shown in Fig. 3C. Average *T*_50_ is based on three devices tested at 1.0 mA cm^−2^. Aver., average; max, maximum; *L*_max_, maximum luminance; P.F._Max_, maximum photon flux.

NPs	Major EL ALN	Major peak (nm)	FWHM (nm)	EQE_aver._ (%)	EQE_Max._(%)	P.F._Max._ photon·(sr^−1^ s^−1^)	*L*_max._ (cd m^−2^)	Aver. *T*_50_ at 1.0 mA cm^−2^ (min)
**MeOH**_**(*n* = 3)**_	3	607	29	7.4 ± 1.8	11.3	8.84 × 10^13^	2.25 × 10^3^	11 ± 3
**EtOH**_**(*n* = 4)**_	4	638	42	15.8 ± 3.1	21.3	2.22 × 10^14^	2.22 × 10^3^	92 ± 2
***n*-BuOH**_**(*n* = 4)**_	4	638	43	21.4 ± 1.8	26.7	9.21 × 10^13^	8.18 × 10^2^	88 ± 3
**IPA**_**(*n* = 4)**_	4	638	50	19.7 ± 1.7	25.1	6.32 × 10^13^	5.83 × 10^2^	110 ± 25
**EtOH**_**(*n* = 5)**_	5	669	42	22.7 ± 2.0	26.8	1.30 × 10^14^	4.46 × 10^2^	252 ± 51
***n*-BuOH**_**(*n* ≥ 7)**_	≥7	728	43	8.3 ± 2.5	13.6	8.50 × 10^13^	7.13 × 10^1^	267 ± 42

### Synthesis of NPs and method for tailoring EL

The FEPS method provides a concept for the synthesis of perovskite nanomaterials (NMs; including nanocrystals or NPs; comparison with previous methods is in table S2), which is much simplified and relies on “environmentally green” precursors. In a typical synthesis process (Fig. 1D, table S3, and movie S1), A-site cation halide [AX, e.g., MA iodide (MAI)] dissolved in polar solvent [e.g., H_2_O and alcohols such as methanol (MeOH), ethanol (EtOH), *n*-butanol (*n*-BuOH), and isopropanol (IPA)] solutions was injected rapidly into the lead halide (PbX_2_, e.g., PbI_2_) precursor solutions with ligands oleic acid (OA) and oleylamine (OM) at 70° to 120°C, followed by immediate flash evaporation of the polar solvent via the Schlenk line connected with a vacuum pump. This method is widely applicable to prepare all APbX_3_ perovskite NMs with high photoluminescence quantum efficiency (PLQE; tables S3 and S4 and figs. S8 and S9). The NPs were separated and then followed a different washing process using pure ethyl acetate (EtOAc) for different ALN LEDs. The method of tailoring different ALN emission of MAPbI_3_ NP LEDs majorly relies on (i) *n*-phase distribution control via using solvents with different polarity in the FEPS synthesis process and the following postwashing process and (ii) CT variation relying on conductivity adjusted via the surface ligands/Pb ratio during the washing process.

### *n*-Phase distribution and surface composition

**Fig. 2.**
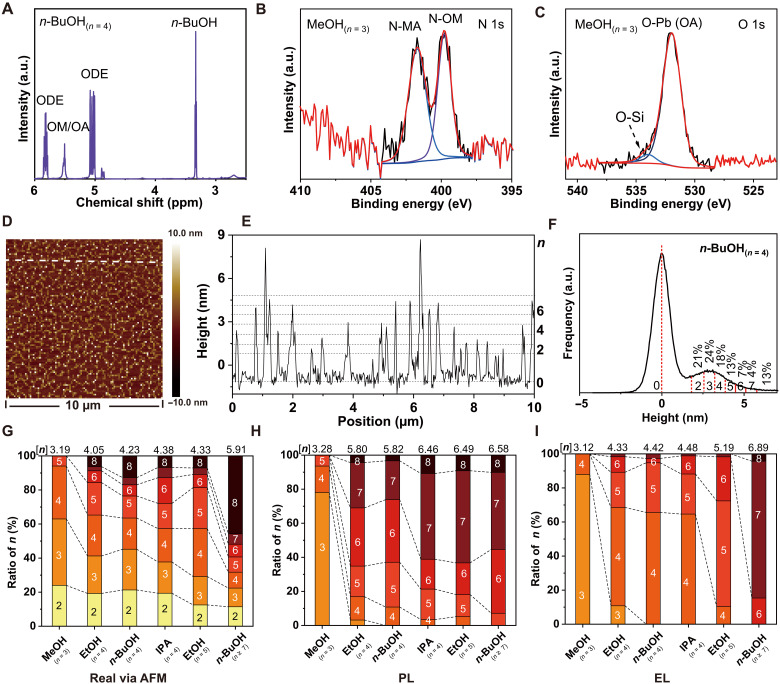
Surface composition and *n*-phase distribution of MAPbI_3_ NPs and LEDs. (**A**) ^1^H-NMR of MAPbI_3_ NP solutions prepared by *n*-BuOH and dispersing NPs in deuterated toluene (T-d_8_, C_6_D_5_CD_3_, toluene-d_8_). (**B**) XPS, N 1s {N from MA^+^ [binding energy (BE), 401.5 eV] and OM^+^ [BE, 399.7 eV]}. (**C**) XPS, O 1s [O from Si-O of the substrate and O from O-Pb (OA)] of MAPbI_3_ NPs prepared by MeOH; spin-coated on a silicon wafer with silica layer; more data are in fig. S17. (**D**) AFM image with a dashed line of a discontinuous film for height and *n*-phase distribution test of NPs. (**E**) Height along the dashed line. (**F**) AFM height statistics result of MAPbI_3_ NPs synthesized by *n*-BuOH, and AFM samples were prepared by spin coating diluted NP solutions (0.5 mg ml^−1^) on a silicon wafer to form a discontinuous film (not for a roughness test). (**G**) Real [*n*] and real *n*-phase ratio distribution of NPs via AFM; more data are in fig. S18. (**H**) [*n*] in PL and ratio of different *n*-phases from the fitting results of PL of NP films. (**I**) [*n*] in EL and ratio of different *n*-phases from the fitting results of EL of LEDs; fitting details are in fig. S2. The average [*n*] is defined as $\left[n\right]=\sum_{n=1}^{8}R_{n}n$, *R_n_* is the ratio (%) of *n*-phases. Figure 2 (G to I) is from the same group of NPs, NP films, and LED samples. ppm, parts per million.

In most cases of MAPbI_3_ NPs prepared by the FEPS method, multiple ALN components were observed. Transmission electron microscopy (TEM) images (Fig. 1E and fig. S10) show that the MAPbI_3_ NPs have a rectangle plate-like shape with a length/width of >10 nm and a lateral thickness of ~2 to 6 nm. Multiple specific components at ~550, 580, 610, 640, 670, 700, 730, and 765 nm in the PL of MAPbI_3_ NP solutions/films (figs. S2 and S11 to S15) correspond to the NPs with defined thicknesses of 1, 2, 3, 4, 5, 6, 7, and ≥8 ALN [PbI_4_] (Fig. 1F). The surface of NPs is terminated with protonated OM (C_18_H_35_NH_3_^+^, OM^+^) and deprotonated OA (C_18_H_35_COO^−^, OA^−^) (*48*) as proved by proton nuclear magnetic resonance (^1^H-NMR; Fig. 2A and fig. S16) and x-ray photoemission spectroscopy (XPS; Fig. 2, B and C, and fig. S17) analysis. We note that the simplified “MAPbI_3_” is used to represent the NPs whose formula should be (OM)_2_MA_*n*−1_Pb*_n_*I_3n + 1−*x*_OA*_x_*.

An average [*n*] was defined as the cumulative weight of different *n*-phases to describe the overall *n*-phase distribution and make the distribution trend and the comparison between real [*n*], [*n*] in PL, and [*n*] in EL more intuitive. The difference of real [*n*], [*n*] in PL, and [*n*] in EL indicates the carrier/energy transfer dynamics under optical and electrical excitations. The real *n*-phase distribution/real [n] was estimated from the atomic force microscopy (AFM) height statistics results (Fig. 2, D to G, and fig. S18). MAPbI_3_ NPs prepared by MeOH show real [*n*] = 3.19 and a typical height of ~3 nm majorly from *n* = 3 to 4 ALN NPs, while MAPbI_3_ NPs prepared by EtOH, *n*-BuOH, and IPA show the increasing [*n*] = 4.05 to 4.38 and the height between ~2 and 6 nm from *n* = 2 to ≥8 ALN NPs. The excitonic absorption features corresponding to different ALN NPs were observed in ultraviolet-visible (UV-vis) absorption spectra of NP solutions (fig. S19). Diffraction patterns from small *n* are obviously higher in MAPbI_3_ NPs prepared by MeOH than those prepared by EtOH, IPA, and *n*-BuOH in x-ray diffraction (XRD; fig. S20). Both the real [*n*] via AFM (3.19 to 4.38) and [*n*] in PL (3.28 to 6.46) of NP films shift monotonously to larger values with the decreasing polarity from MeOH to IPA (Fig. 2, G to I; figs. S11 to S15 and S21 to S23; and table S5). These results demonstrate that the *n*-phase distribution was modulated by applying polar solvents with different polarity. The reason can be ascribed to that weaker hydrogen bond, and other intermolecular interactions (proved by NMR in fig. S24 and solubility of MAI in different alcohols is shown in fig. S22 and table S5) between [PbI_4_] skeleton, MA^+^, and alcohols with smaller polarity mean the weaker ability to dissolve [PbI_4_] skeleton for the assembly process of MA^+^ and I^−^ ions to form the perovskite flakes and lastly to produce more large *n* components. The posttreatment further changed the *n*-phase distribution (Fig. 2G and fig. S18). As for *n* = 5 NPs, the additional one-time washing process caused the real [*n*] to slightly shift from 4.05 to 4.33 as proved by AFM. As for *n* ≥ 7 NPs, additional three times washing processes of EtOAc caused obvious aggregation/merging of NPs with the real [*n*] shifting from the normal *n*-BuOH NP [*n*] = 4.23 to 5.91.

### Performance and stability of LEDs

**Fig. 3.**
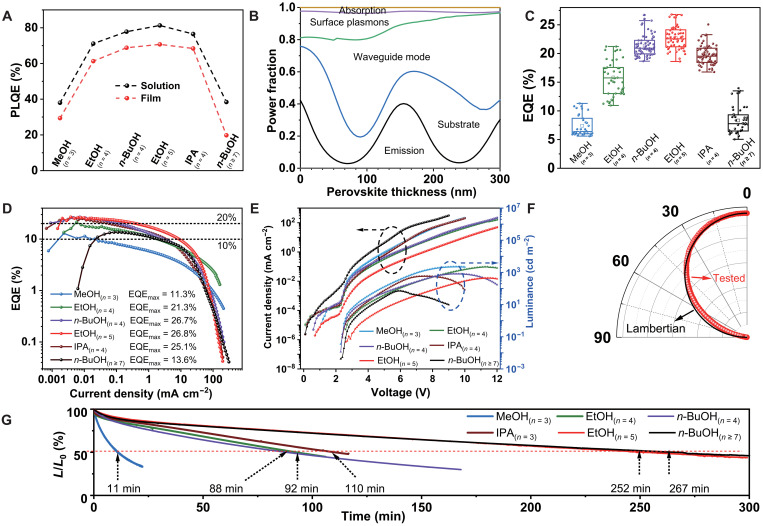
Performance of MAPbI_3_ NP LEDs with tailored ALN emission. NPs were prepared by MeOH_(*n* = 3)_, EtOH_(*n* = 4)_, *n*-BuOH_(*n* = 4)_, IPA_(*n* = 4)_, EtOH_(*n* = 5)_, and *n*-BuOH_(*n* ≥ 7)_, correspondingly. (**A**) PLQE of NP solutions and films, average value of three to five time tests, detailed data in table S6. (**B**) Optical simulation of LEDs with different thickness perovskite NP layers. (**C**) EQE distribution of MAPbI_3_ NP LEDs obtained from 34, 46, 62, 55, 67, and 38 devices, correspondingly. (**D**) EQE–current density curves of the most efficient LEDs. (**E**) Current density-voltage (*J*-*V*) and luminance-voltage curves of NP LEDs. (**F**) Angle-dependent EL of NP LEDs and ideal Lambertian profile. (**G**) Stability test of LEDs at 1.0 mA cm^−2^ with average original peak EQE = 7.1, 17.4, 22.1, 23.9, 21.5, and 7.9% and initial luminance of 132.5, 99.3, 82.1, 41.8, 95.2, and 8.6 cd m^−2^, correspondingly; the curves are obtained by averaging results of three devices at each condition to reduce deviation. *L*/*L*_0_, real-time luminance (*L*)/original luminance (*L*_0_).

Both PLQE of NP solutions and films and EQE of LEDs showed a “volcanic” shape distribution from MeOH_(*n* = 3)_ to *n*-BuOH_(*n* ≥ 7)_ (Fig. 3, A to C; table S6; and data S1). Results of photon outcoupling models prove that this optimized LED structure supports ~34% EQE at most (>25% EQE with ~70% PLQE) with ~10-nm emitting layer (Fig. 3B) (*49*). As shown in Fig. 3 (C to E), figs. S25 and S26, and Table 1, *n* = 3 at 607 nm LEDs prepared by MeOH show a peak EQE of 11.3% (average, 7.4 ± 1.8%) with sharp EL spectra, and this wavelength is near edge of the orange/red spectrum and means a higher photopic luminance (2.25 × 10^3^ cd m^−2^). The LEDs for *n* = 4 emission at 638 nm prepared by *n*-BuOH show a peak EQE of 26.7% (average, 21.4 ± 1.8%) with a maximum luminance of 8.18 × 10^2^ cd m^−2^ which could be further improved to 4.33 × 10^3^ cd m^−2^ with a peak EQE of 21.1% (fig. S26). These LEDs reach the peak EQE between ~0.01 and 0.02 mA cm^−2^ with a luminance of ~2 to 4 cd m^−2^, and it remains >25, >20, and >10% at ~0.02, ~0.1–0.2, and ~3 mA cm^−2^ with luminance of ~4, ~20, and ~200 cd m^−2^, correspondingly. The EtOH_(*n* = 5)_ LEDs for *n* = 5 at 669 nm achieved the highest efficiency with the peak EQE of 26.8% (average, 22.7 ± 2.0%), and EQE remains >25% at 0.023 mA cm^−2^, >20% at 0.40 mA cm^−2^, and >10% at 8.35 mA cm^−2^. EL emission of *n*-BuOH_(*n* ≥ 7)_ LEDs shifted to *n* ≥ 7 at ~728 nm with a peak EQE of 13.6% (average, 8.3 ± 2.5%). Angle-dependent EL measurement (Fig. 3F) reveals that the radiation distribution of these NP-based LEDs is Lambertian. The luminance of these MAPbI_3_ NP LEDs demonstrates an obvious decreasing trend with EL from an increasing *n* value due to the decreasing photopic sensitivity, which can be further improved ~8 times at most to the highest one of 4.62 × 10^3^ cd m^−2^ by increasing the thickness of NP layer and reducing the thickness of CT layers (fig. S26).

The operational stability and color stability of these perovskite NP-based LEDs were also studied in ambient air at a current density of 1.0 mA cm^−2^ (Fig. 3G and Table 1). The average operating half-lifetime (*T*_50_, the time that the original luminance is halved) of LEDs showed obvious relevance with the *n* values. The stability of *n* = 3 LEDs is the worst with *T*_50_ of only 11 ± 3 min. The *n* = 4 LEDs showed similar *T*_50_ of ~100 min. The *n* = 5 and *n* ≥ 7 LEDs showed the best stability with the longest *T*_50_ of 267 ± 42 min. These LEDs demonstrate improved stability compared with previous MA-based NP/QD LEDs in which MAPbI_3_ is generally thought to be less stable because of a volatile organic MA component (comparison is in table S1). These pure halide MAPbI_3_ NP LEDs also demonstrate high color stability without observable EL peak shift during the same stability test (fig. S27).

### PL-EL difference

As shown in fig. S3 (E and F), EL and PL spectra of the bulk quasi-2D system fabricated from the precursor solutions with a stoichiometric ratio of nominal *n* = 3 PEA_2_MA_2_Pb_3_I_10_ are very similar. However, both its EL and PL red shift from the stoichiometric ratio of *n* = 3 (~610 nm) to *n* = 6 to 7 with unfixed inter-ALN peaks between 686 and 724 nm. This shift can be ascribed to the energy/charge funneling which cascades energy/charge along the “ladder-like” energy levels, and the emission ultimately accumulates to the large *n* with the narrowest bandgap (*45*, *50*, *51*). In contrast, the situation is quite different in MAPbI_3_ NP LEDs. The overall trend demonstrated in Fig. 2 (G to I) is that real [*n*] via AFM < [*n*] in EL < [*n*] in PL. A much higher ratio of photons emits from small *n* rather than large *n* NPs in EL, making the EL of these NP LEDs always blue-shifted (the largest, 89 nm) compared to the PL of its counterpart NP films/solutions (Fig. 2, H and I, and figs. S2 and S21). To the best of our knowledge, such a huge PL-EL difference has never been observed before in the optoelectronic field. This big PL-EL difference in this NP system indicates that the energy transfer routes are different under optical and electrical excitations, and the carrier dynamics of these NP systems is also very different from conventional bulk quasi-2D perovskites.

### Carrier dynamics and photophysics

**Fig. 4.**
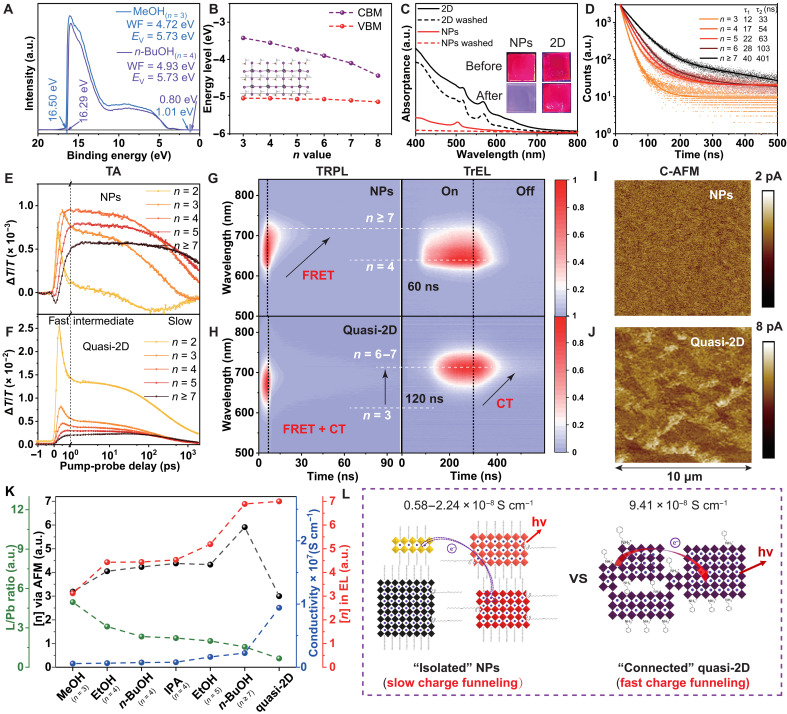
Carrier/energy dynamics of MAPbI_3_ NPs and comparison with bulk quasi-2D perovskite. (**A**) UPS of MAPbI_3_ NPs prepared by MeOH and *n*-BuOH; more data are in fig. S28. (**B**) Energy levels (referenced to the vacuum level) of different ALN MAPbI_3_ NPs by DFT simulations; the inset is the *n* = 4 model for simulation. (**C**) UV-vis and photos of NP film and quasi-2D film before/after washing by toluene. (**D**) PL decay kinetics (λ_exc_ = 405 nm) with fitted lifetime of MAPbI_3_ NP solution prepared by *n*-BuOH, collected at different wavelengths for *n* = 3 to ≥7, respectively. Decay kinetics in TA spectra measurement of (**E**) NPs and (**F**) quasi-2D films; more data and fitting results are in figs. S29 and S30; the excitation fluence for the TA measurement was 5.09 μJ cm^−2^. TrEL (0 to 300 ns, turn-on; 300 to 600 ns, turn-off) and TRPL of (**G**) NPs prepared by *n*-BuOH and (**H**) quasi-2D LEDs/films; more data are in fig. S32. Conductive AFM (C-AFM) of (**I**) NPs prepared by *n*-BuOH and (**J**) quasi-2D films. (**K**) Correlation of ligand/Pb ratio (L/Pb), conductivity, average real *n*-phase ([*n*] via AFM), and average *n*-phase in EL ([*n*] in EL); more details are in fig. S31 and table S7. L/Pb was obtained from XPS data. (**L**) Schematic of CT in multi–*n*-phase NP film with slow charge funneling and quasi-2D film with normal charge funneling. WF, work function.

Ultraviolet photoelectron spectroscopy (UPS; Fig. 4A and fig. S28) and density functional theory (DFT) calculations (Fig. 4B) were carried out and prove that the decreasing bandgap mainly arises from the lowering of the conduction band minima (CBM) levels, while the valence band maxima (VBM) levels remain almost constant across the increasing *n* value. These ladder-like levels can cascade energy/charge from small to large *n* NPs. The CT and FRET could be possible major energy transfer routes in NP and quasi-2D systems under optical/electrical excitations. NP films can be easily dissolved in toluene as shown in UV-vis gives intuitive evidence that NPs in films are much more “isolated” (more soluble) than the bulk quasi-2D film (Fig. 4C). This “isolation” has a different influence on FRET and CT (more discussions are in the Supplementary Materials).

Combined measurements were used to reveal which route dominates under optical/electrical excitations and the difference between conventional bulk quasi-2D perovskites and these NPs. The gradually increasing τ_1_ and τ_2_ components in a time-resolved PL (TRPL) test demonstrated decreasing recombination rate as the *n* value increases (Fig. 4D). Transient absorption (TA) spectroscopy provides information of the fast energy/CT process in a subnanosecond timescale under optical excitation (Fig. 4, E and F, and figs. S29 and S30), in which the fast decay-rise process at the ~1-ps timescale can be ascribed to FRET from small to large *n* NPs and the carriers’ cooling process (*52*, *53*). Following the fast ~1-ps process, the intermediate stage decays with a typical gradually increasing lifetime from ~20 to ~100 ps as *n* increases. This intermediate process in the subnanosecond timescale can be also ascribed to FRET (*46*). The rate of FRET in NP films is slightly slower or similar to shorter lifetimes compared with the quasi-2D films, because the isolation in NP films does not obviously change the distance (the size of NPs, >10 nm) in the FRET process. So the obvious concentrating trend from small to large *n* could also be found in the TRPL of NP films, and the PL of both bulk quasi-2D and NP films is majorly from large *n* (Figs. 2H and 4, G and H).

However, the excitation and the following charge/energy transfer processes are quite different in EL, in which electrons and holes inject separately from CT layers to perovskite layers. The “separated” injection of holes and electrons strongly reduces the possibility of FRET. The CT becomes the major energy transfer route in EL. The more isolated nature of NP films can make the CT from small to large *n* less efficient than in the bulk 2D films. The conductivity of films was used to estimate the CT rate. The conductivity of NP films is 0.58 to 2.24 × 10^−8^ S cm^−1^, which is much smaller than 9.41 × 10^−8^ S cm^−1^ of the bulk quasi-2D film (fig. S31 and table S7). The tunneling current of NP films is only about one-fourth of quai-2D film in the conductive AFM (C-AFM) test (Fig. 4, I and J). Transient EL (TrEL; Fig. 4, G and H, and fig. S32) gives further evidence of electronic process difference in a submicrosecond timescale in these NP LEDs and quasi-2D LEDs (*54*). *T*_d_ is defined as the delay time from the turn-on of a pulse voltage to the onset of TrEL, which is related to the CT process (*55*). The τ_d_ increases from 60 and 80 to 120 ns in *n* = 4, 5, to ≥7 NP LEDs, while the τ_d_ of quasi-2D LEDs is 120 ns. It demonstrates a prolonged CT process from small to large *n* NPs and a slower carrier hole transfer layer (HTL)/interface injection in large *n* NPs and quasi-2D LEDs. The obvious concentrating trend from small to large *n* could be found in the TrEL of quasi-2D LEDs with long decay tailing of large *n*-phase. In contrast, the decay tailing of TrEL appears in small *n* due to the charge funneling via CT being much slower in NP LEDs.

The correlation analysis (Fig. 4K, fig. S31, and table S7) demonstrates that two factors influence the [*n*] in EL: (i) The real *n*-phase (real [*n*] via AFM) distribution is the dominating factor and the prerequisite of this tailored ALN emission; (ii) the conductivity of the films, which is inversely correlated with the numbers of ligands (L/Pb) on the surface. The improved conductivity can shift the [*n*] in EL to a larger value (Fig. 2, G to I). Moreover, additional one to two EL peaks from larger *n* (fig. S33) appear after the ligand exchange process, which removes more ligands and benefits the CT (*7*). All of the systematic evidence indicates that the CT is the major energy/CT route in working LEDs, and the slower CT and the low probability of FRET block the further shift of EL to a larger *n* value, resulting in a big PL-EL difference in the NP system (Fig. 4L). It is also the prerequisite to achieve fixed/discrete emission in NP LEDs instead of unfixed inter-ALN emission in quasi-2D LEDs (*56*).

## DISCUSSION

In conclusion, we demonstrated tailored ALN EL in perovskite NP LEDs and achieved highly efficient and color-stable LEDs with specific, sharp, and tunable EL. This concept opens up new avenues for wavelength tunability of emission based on emitters with specific layers of atoms, which are not susceptible to preparation conditions in contrast with the size or composition control strategies in conventional QDs and perovskites. This work simplifies preparation requirements and is promising to make the scale production of QDs and its LEDs easier, cheaper, and more reproducible for the display industry. This work uses environmentally green precursors and obviates the need of some presynthesized precursors in the traditional hot injection method for colloidal perovskite NM preparation. The study of carrier dynamics reveals that CT is the major energy transfer route in working perovskite LEDs, and the slower CT and the low probability of FRET result in a large blue shift of EL compared to its PL in these NP systems. This study of carrier dynamics and photophysics also settles the argument about the energy transfer route via FRET or CT in a multiphase system, and this will enlighten the reasonable design of NP LEDs.

## MATERIALS AND METHODS

### Chemicals

All chemicals were used as received without further purification. CsCl (99.0%, Sigma-Aldrich), CsBr (99.5%), CsI (99.9%, Sigma-Aldrich), MA chloride (MACl), MA bromide (MABr), MA iodide (MAI), formamidinium chloride (FACl), formamidinium bromide (FABr), formamidinium iodide (FAI), and phenethylammonium iodide were purchased from Greatcell Solar Materials. PbCl_2_ (99.99%, TCI Chemicals), PbBr_2_ (99.99%, Aladdin), PbI_2_ (99.99%, TCI Chemicals) were stored in N_2_ glovebox. OA (99.0%), OM (70%) octadecene (ODE), EtOAc, MeOH, EtOH, isopropyl alcohol (IPA), *n*-butyl alcohol (1-butanol, *n*-BuOH), toluene, xylene, deuterated toluene (T-d_8_, C_6_D_5_CD_3_, toluene-d_8_), deuterated ethanol (EtOH-d_6_, C_2_D_5_OD), and *N*,*N*-dimethylformamide were anhydrous and purchased from Sigma-Aldrich. The following chemicals were also used: poly(3,4-ethylenedioxythiophene) polystyrene sulfonate (PEDOT:PSS; Ossila), poly[*N*,*N*′-bis(4-butylphenyl)-*N*,*N*′-bis(phenyl)-benzidine] (poly-TPD; American Dye Source), poly[(9,9-dioctylfluorenyl-2,7-diyl)-co-(4,4′-(*N*-(p-butylphenyl))diphenylamine)] (TFB; American Dye Source), 2,2′,2″-(1,3,5-benzinetriyl)-tris(1-phenyl-1-H-benzimidazole) (TPBi; Ossila), and lithium 8-hydroxyquinolinolate (Liq; Ossila).

### Synthesis of APbX_3_ perovskite NPs

All perovskite NPs were synthesized via a standard Schlenk line with a vacuum pump under ambient conditions in a fume hood at both Peking University and the University of Cambridge. As shown in Fig. 1D and table S3, APbX_3_ NPs were synthesized as follows: Lead halide (PbX_2_; 1.0 mmol) was added into a three-neck round bottom flask (100 ml), and then a certain amount of OA, OM, and ODE was added into the flask. The solutions were degassed at 120°C until PbX_2_ completely dissolved and bubbles no longer emerged by using the Schlenk line. At the same time, 1.0 mmol of CsX (CsCl, CsBr, CsI), MAX (MACl, MABr, MAI), and FAX (FACl, FABr, FAI) precursors was dissolved in corresponding polar solvents with heating and then cooled down to room temperature. The PbX_2_ precursor solutions were cooled down to a certain temperature (70° to 120°C) for injection. AX precursor solutions were injected into PbX_2_ precursor solutions with fast stirring. Then, polar solvents were removed immediately by the Schlenk line until bubbles no longer emerged to ensure the complete removal of polar solvents. NPs were separated from the crude solutions by centrifugation at 14,000 rpm and then washed with toluene, EtOAc, etc. The NPs prepared by MeOH for *n* = 3 ALN emission and normal EtOH, *n*-BuOH, and IPA MAPbI_3_ for *n* = 4 ALN emission NP solutions (2.0 mg ml^−1^) were washed once by EtOAc, and the EtOH_(*n* = 5)_ NP solution for *n* = 5 ALN emission at 670 NP LEDs was prepared from the same solution of the normal EtOH sample with an additional washing process of EtOAc. The *n*-BuOH_(*n* ≥ 7)_ NP sample for *n* ≥ 7 ALN emission at 728 NP LEDs was prepared from the normal *n*-BuOH NP solution with additional three times washing processes of EtOAc. NPs were redispersed in toluene, and large particles were removed by subsequent centrifugation and filtering. The concentration of NP solutions was measured by UV-vis absorption spectrometry, and then solutions were diluted to 2.0 mg ml^−1^ for LED preparation. This synthesis method was cross-checked and repeated dozens of times by different authors both at the University of Cambridge and Peking University.

### Fabrication of MAPbI_3_ NP LEDs

All MAPbI_3_ NP LEDs were prepared in the Optoelectronics Group of Cavendish Laboratory, University of Cambridge. Indium tin oxide (ITO) substrates were cleaned by an ultrasonification process in deionized water, acetone, and IPA for 10 min. After the 10-min plasma treatment, PEDOT:PSS was spin-coated onto ITO substrates at 4000 rpm for 40 s, followed by an annealing process at 150°C for 10 min. Poly-TPD solution (10 mg ml^−1^ in chlorobenzene) and TFB solution (10 mg ml^−1^, in xylene) were sequentially spin-coated onto the substrates at 4000 rpm for 40 s and annealed at 150°C for 10 min. The MAPbI_3_ NP (2.0 mg ml^−1^) solutions were spin-coated onto the substrates at 2000 rpm for 30 s in an N_2_-filled glove box. TPBi (110 nm), Liq (1 nm), and Al (100 nm) were sequentially deposited using a thermal evaporation system. For the LEDs with high luminance, the concentration of NP solutions was increased to 4.0 mg ml^−1^ for the thicker emission layer, the concentration of poly-TPD and TFB solution was reduced to 6 mg ml^−1^, and the thickness of TPBi was reduced to 60 nm. LEDs were encapsulated with glass in the glove box.

### DFT calculations

All DFT calculations were carried out using the Quantum Espresso suite (v7.0) (*57*, *58*). Perdew-Burke-Ernzerhof functional was used to approximate the exchange correlation (*59*), and the ultrasoft pseudopotentials from the GBRV library were used to treat the core-valence interactions (*60*). The electronic wave functions were expanded in a plane wave basis with a charge density cutoff of 200 rydberg (Ry) and a cutoff of 40 Ry, and the dispersion correction was included empirically using the DFT-D3 method (*61*). The Brillouin zone was sampled with a Γ-centered Monkhorst-Pack *k*-point grid of 6 × 6 × 6 (*62*). The atoms are relaxed until the Hellman-Feynman force converges below 0.01 eV Å^−1^, and the bulk volume is relaxed until all components of the stress tensor are below 10^−2^ GPa. For the n-layered perovskites, a vacuum spacing of 20 Å was added to the supercell in the *z* direction to remove any spurious interactions, and a commensurate 6 × 6 × 1 *k*-point grid was used. A saw-like potential simulating an electric field was added to the bare ionic potential, to correct for the dipole moment in the *z* direction induced by the polar MA^+^ ion. We note that the bandgap of the perovskites is lower than experimentally measured values, due to the well-known underestimation of semiconductor bandgaps by DFT, but the trend observed here is valid. From a molecular orbital perspective, the VBM of the perovskites is dominated by the antibonding orbitals of Pb 6s and I 5p at the R point in the Brillouin zone. The spherical symmetry of the Pb 6s orbital and the complete number of PbI_6_ octahedral in these layered perovskites ensure that the VBM energies are not affected by the surface termination. For CBM on the other hand, it is mainly composed of the Pb 6p orbitals. The surface termination breaks the symmetry of the 6p orbitals, as those that lie in parallel in the *xy* (6p*_x_* and 6p*_y_*) plane are in a different energy than the 6p*_z_* orbitals. Therefore, the quantum confinement effect in the *z* direction is mainly manifested by the splitting of the orbital energies. This in turn means that the position of the CBM lowers in energy, as the number of perovskite layers increases, weakening the quantum confinement effects and the energy splitting in the p orbitals (*63*–*66*).

## Supplementary Materials

### The PDF file includes:

Supplementary Materials and Methods Tables S1 to S7 Figs. S1 to S33 Legend for movie S1 Legend for data S1 References
