# Supplementary material for: Efficient perovskite LEDs with tailored atomic layer number emission at fixed wavelengths
Source: Sci Adv. 2025 Feb 14;11(7):eadp9595. doi: 10.1126/sciadv.adp9595 (PMC11827643; doi:10.1126/sciadv.adp9595)
Supplement: Supplementary file 1 — Supplementary Materials and Methods Tables S1 to S7 Figs. S1 to S33 Legend for movie S1 Legend for data S1 References [file sciadv.adp9595_sm.pdf]

Science Advances
AAAS

Supplementary Materials for

Efficient perovskite LEDs with tailored atomic layer number emission at fixed wavelengths

Ligang Wang *et al.*

Corresponding author: Ligang Wang, ligangwang@pku.edu.cn; Huanping Zhou, happy_zhou@pku.edu.cn; Ling-Dong Sun, sun@pku.edu.cn; Chun-Hua Yan, yan@lzu.edu.cn; Richard H. Friend, rhf10@cam.ac.uk

Sci. Adv. **11**, eadp9595 (2025)
DOI: 10.1126/sciadv.adp9595

The PDF file includes:

Supplementary Materials and Methods
Tables S1 to S7
Figs. S1 to S33
Legend for movie S1
Legend for data S1
References

Other Supplementary Material for this manuscript includes the following:

Movie S1
Data S1

Materials and Methods

Fabrication of quasi-2D films and LEDs

Precursor solution of quasi-2D (n = 3) perovskite was prepared with a stoichiometric ratio of nominal n = 3, $PEA_2MA_2Pb_3I_{10}$ by dissolving MAI (50 nmol, 7.9 mg), PEAI (50 nmol, 12.5 mg), PbI_2 (75 nmol, 34.6 mg) in 1.5 mL DMF. Films and LEDs were fabricated by spin-coating the precursor solution on substrates. Other steps are the same as NP films/LEDs.

Characterization of LED performance

All $MAPbI_3$ NP LEDs were measured under ambient conditions in the Optoelectronics Group of Cavendish Laboratory, University of Cambridge. The detailed procedures and information of the setup employed can be found in our previous paper (*68*). LEDs were mounted with aluminum legs and placed on a device holder and electrically connected with a source measure unit (Keithley K2400) that acted as voltage source for the current density-voltage (J-V) characteristics measurements. The active area of the LEDs is 4.5 mm^2. The photon flux was simultaneously collected by using a calibrated silicon photodiode with a circular geometry placed right above the devices. The responsibility of the photodetector is provided by the manufacturer. The EL spectra of the devices were collected by a Labsphere CDS 610 spectrometer (cross-checked against Edinburgh Instruments FLS980 Spectrometer in Cavendish Laboratory). The photodiode quantum efficiency was calculated based on the EL spectra of each device. The photopic curve employed in this paper uses a function normalized at a wavelength of ~555 nm with ~683 $lm\cdot W^{-1}$. The conversion factor ($lm\cdot s\cdot photon^{-1}$) was calculated by the EL spectra of each device. The luminance value ($cd\cdot m^{-2}$) of LEDs was calculated based on the conversion factor and emission function of the LEDs, and the EQE of the devices were calculated assuming a Lambertian emission profile. The performance of these LEDs was double and cross-checked by different authors.

Cross-check of LED performance measurement setup

To confirm the accuracy of the LED performance measurement setup employed in the Optoelectronics Group of Cavendish Laboratory. We performed a cross-check of the setup with Prof. Tae-Woo Lee's group at Seoul National University. Firstly, a batch of organic LEDs (OLEDs) were prepared and tested in Prof. Tae-Woo Lee's group. The OLEDs were taken to Cavendish Laboratory by flight immediately. The performance of OLEDs was tested again by the setup in Cavendish Laboratory. The EQE and related parameters of OLEDs matched almost perfectly.

EL spectra angular distribution of NP LEDs

EL spectra angular distribution was tested in a homebuilt set-up equipped with a Thorlabs motor (the accuracy of the angle is less than 1°) for angle adjustment. LEDs were electrically connected with a source measure unit (Keithley K2400) that acted as a voltage source at 6 V. The photons were collected by a fiber connected with an Andor iDus DU420A Si detector for spectra and intensity measurement.

Optical modelling of NP LEDs

In this model, we assumed the charge injection is well balanced with $\gamma = 1$, and the intrinsic quantum yield of perovskite thin film is assumed to be 95%. The emission zone is assumed to be infinitely thin and located at the bottom of the perovskite layer. The dipole orientation of emitters is assumed to be isotropic. The refractive index and thickness of each layer in the LED stack are from previous papers. The optical power generated in red PeLEDs is distributed to four channels, separated by the in-plane wavevector $k_{//}$. (1) Direct emission: $k_0\cdot n_{air} \geq k_{//} \geq 0$, where $k_0 = 2\pi/\lambda$ is the vacuum wave vector and n_{air} is the refractive index of air. In this region, light emits into the air from NP LEDs. (2) Substrate mode: $k_0\cdot n_{sub} \geq k_{//} \geq k_0\cdot n_{air}$, where n_{sub} is the refractive index of the substrate. In this region, light is trapped in the substrate because of the total internal reflection (TIR) at the interface of the substrate and air. (3) Waveguide mode: $k_0\cdot n_{eff} \geq k_{//} \geq k_0\cdot n_{sub}$, where n_{eff} is the effective refractive index of the functional layers (from ITO to TPBi). In this region, light is trapped in functional layers. (4) Surface plasmon mode: $k_{//} \geq k_0\cdot n_{eff}$. In this region, light couples to the top metal electrode in the form of evanescent waves. For the four optical channels, part of the dissipated power is absorbed by each layer inside the device instead of leaking into the corresponding optical channel.

Conductivity test based on 4-probe method

The conductivity of NM, 3D $MAPbI_3$ and quasi-2D films was tested based on the 4-probe method. The electrode pattern was designed by AutoCAD, the gap between the 4 in-line electrodes is 135 μm with length of 2 mm. The pattern was defined by ultra-violet (UV) lithography (MLA150) using photo-positive resists (S1813) and developer (MF319). 10 nm adhesive chromium (Cr) and 90 nm gold (Au) were deposited on the quartz substrate, then followed a lift-off process to get the designed electrode pattern. The condensed NM, 3D $MAPbI_3$ and quasi-2D solutions were spin-coated on the substrates with

the designed electrode. The perovskite layer except for the electrode area (2 × 2 mm) was removed under optical microscopy. The thickness of perovskite films was checked by a profiler. All measurements were measured using Agilent 4155B SPA in an N_2-filled glovebox.

Transmission electron microscopy

Transmission electron microscopy (TEM) measurements were taken by JEM-2100 (JEOL Ltd.) operated at 200 kV. The TEM samples were prepared by dropping NP solutions onto copper TEM grids and drying them in the air. TEM images of NPs were cross-checked both at the University of Cambridge and Peking University.

Scanning electron microscopy

Scanning electron microscopy (SEM) measurements were carried out by Magellan 400 with an acceleration voltage of 3 kV. Perovskite NP film samples were prepared by spin-coating NPs on ITO/PEDOT:PSS/poly-TPD/TFB substrates. Cross-sectional images of the LED structure were carried out by cutting LED devices with a diamond pen.

X-ray photoelectron spectrometer and ultraviolet photoelectron spectroscopy

X-ray photoelectron spectrometer (XPS) data were acquired by AXIS Supra (Kratos Analytical Ltd) equipped with monochromatic Al Kα (1486.69 eV) X-rays at 150 W, with an energy step 0.1000 eV and total acquisition time ~60 s. The energy resolution is 0.48 eV for Ag $3d_{5/2}$. The depth is less than 10 nm in surface-sensitive tests for quantitative and semi-quantitative measurements. The spectra were calibrated to the main line of the carbon 1s spectrum (adventitious carbon) set to 284.8 eV. Ultraviolet photoelectron spectroscopy (UPS) was used to test the work function and VBM of the $MAPbI_3$ perovskite NP films. UPS data were also obtained by the same instrument as XPS with an ultrahigh-vacuum chamber equipped with He I ultraviolet light source with the energy of emission line at 21.22 eV, operated at an energy step 0.050 eV. The work function is calculated as $\Phi = 21.22$ eV – onset, as shown in Fig. S28. The gap of valence-band maximum (VBM) to work function is determined by the linear extrapolation of the leading edge of the valence band to zero baseline intensity. Samples were prepared by dropping NP solutions on silicon wafer and dried in an N_2-filled glovebox. Data were analyzed by using the software CasaXPS software.

Nuclear magnetic resonance (^{1}H-NMR)

Nuclear magnetic resonance (^{1}H-NMR) spectra were recorded on a Bruker-600MHz NMR spectrometer equipped with a 14.1 Tesla standard bore magnet and CP BBO 600S3 BB-H&F-D-05 Z probe. All $MAPbI_3$ NP solutions samples were prepared by dispersing NP solids after washing with ethyl acetate and drying at room temperature into 1 mL of deuterated toluene (toluene-d_8). The NMR of EtOH is from the un-purified deuterated EtOH (CD_3CD_2OD) with residual normal EtOH, MAI + EtOH was prepared by dissolving MAI into CD_3CD_2OD. Data were analyzed by Topspin 3.5 software.

Ultraviolet−Visible absorption spectrometry

Ultraviolet-visible absorption spectra (UV-Vis) were recorded on a Shimadzu UV-3600Plus UV-Vis-NIR Spectrophotometer. All solution samples were prepared by dispersing NPs into toluene (< 0.5 $mg \cdot mL^{-1}$ to avoid absorption saturation). The film samples were prepared by spin-coating NPs/quasi-2D solutions onto the fused glass.

Atomic force microscopy (AFM) and conductive AFM (C-AFM)

Atomic force microscopy (AFM) measurements were performed by using a Bruker dimension icon with scansyst air tip, operated at scansyst mode. Samples for height and n-phase distribution tests were prepared by spin-coating $MAPbI_3$ NP solutions (< 0.5 $mg \cdot mL^{-1}$) at 5000 r/min onto silicon substrates in the glovebox. Samples for the roughness test were prepared by spin-coating the final solutions (2.0 $mg \cdot mL^{-1}$) on ITO/PEDOT:PSS/poly-TPD/TFB substrates. C-AFM was obtained with a bias voltage of -1 V under contact mode-based TUNA (contact TUNA) using the same instrument as AFM, the samples were prepared on the conductive heavily doped silicon wafer.

X-ray diffraction

X-ray diffraction (XRD) was performed by a PANalytical X'Pert3 powder diffractometer, using Cu Kα radiation ($\lambda = 1.5406$ Å) radiation source. Spectra were collected with an angular range of $5° < 2\theta < 40°$. Measurements were performed on as-prepared NP films on glass substrates.

Photoluminescence measurements

Photoluminescence (PL) data were obtained by Edinburgh Instruments FLS980 Spectrometer with a xenon lamp. The light from the samples was collected by a Si-based single-photon avalanche photodiode. All solution samples were prepared by dispersing NPs into toluene. The film samples were prepared by

spin-coating $MAPbI_3$ NP solutions (2.0 mg·mL^{-1}) onto corresponding substrates in the glovebox. Lifetimes were obtained by using a time-correlated single-photon counting (TCSPC) appendix with a 405 nm pulsed laser. All of the TRPL decay curves were fitted by a double-exponential decay model with the biexponential equation: $I_{(t)} = A_1 \exp(-t/\tau_1) + A_2 \exp(-t/\tau_2)$. The lifetime of the τ_1 component is ascribed to monomolecular recombination which is related to the surface defect density, higher defect density will induce shorter τ_1 and lower device performance. While the τ_2 component is related to radiative bi-molecular recombination of electron and hole, the large n phase possesses smaller exciton binding energy and insufficient recombination rate for better devices.

Photoluminescence quantum efficiency

Absolute photoluminescence quantum efficiency (PLQE) of $MAPbI_3$ NP solutions and films was measured by Hamamatsu C9920-02 PL quantum yield measurement system with integrating sphere. A continuous-wave 405 nm laser with a ~0.3 mm^2 focused beam spot was used to pump the samples. PLQE was cross-checked with another instrument (Edinburgh Instruments FLS980 Spectrometer with an integrated sphere) both at the University of Cambridge and Peking University.

Time-resolved photoluminescence

Time-resolved photoluminescence (PL) measurements were tested with an electrically gated intensified CCD (iCCD) camera system (Andor iStar DH740 CCI-010) connected to a grating spectrometer (Andor SR303i). Photoexcitation was from the femtosecond laser pulses generated in a homebuilt set-up by second harmonic generation in a beta-barium borate (BBO) crystal from the fundamental output (1.55 eV pulse energy, 80 fs pulse length) of a Ti:sapphire laser system (Spectra-Physics Solstice). The second harmonic laser pulses had a wavelength of 400 nm (3.1 eV). The temporal resolution of the PL emission was obtained by measuring the PL from samples by stepping the intensified CCD gate delay relative to the pump pulse. The gate width was set as 5 ns. The $MAPbI_3$ films were prepared by spin-coating on pre-cleaned fused-silica substrates with the same method described in the device fabrication sections. All solution samples were prepared by dispersing NCs into toluene (∼2 mg·mL^{-1}).

Transient electroluminescence

In the transient electroluminescence (TrEL) test, short square voltage pulses (1 MHz with 300 ns duration) were generated by an HP8116A function generator and applied to the device. A grating spectrometer (Andor SR303i) was used to collect the EL of the LEDs. The gate width was set as 3 ns.

Transient absorption spectroscopy

Transient absorption (TA) studies were performed on home-built setups powered by a Ti:sapphire amplifier (Spectra Physics Solstice Ace, 100 fs pulses at 800 nm, 7 W output at 1 kHz and generating ~100 fs pulses was split into the pump and probe beam paths). The 400-nm pump beam was from the second harmonic generation in a beta-barium borate (BBO, 1 mm thickness, Eksma Optics) crystal of the fundamental output (1.55 eV pulse energy, 80 fs pulse length) of a Ti:sapphire laser system. The pump was blocked by a chopper wheel rotating at 500 Hz while a computer operated a mechanical delay stage (Newport XPS-C8) to adjust the delay between the pump and the probe. The visible broadband beam (520-780 nm) was generated in a home-built noncollinear optical parametric amplifier (NOPA), and the white light was split into two identical beams (probe and reference) by a 50/50 beam splitter. The reference beam passing through the sample did not interact with the pump, which allows for correcting for any shot-to-shot fluctuations in the probe that would otherwise greatly increase the structured noise in the experiments. Based on this arrangement, small signals with a $\Delta T/T$~10^{-5} could be measured. The $MAPbI_3$ NP solution/film samples were excited by a pump pulse and then probed and recorded at different delayed times (0.3-1750 ps) using a broadband probe pulse 520-780 nm. The solution samples were held in a 1 mm quartz cuvette with lids, mounted in a holder. The beams were focused into an imaging spectrometer (Andor Shamrock SR-303i-B) and detected using a pair of dual-line array detector image InGaAs sensors (Hamamatsu G11608-512DA) and Si (Hamamatsu S8381-1024Q) driven and read out at the full laser repetition rate by a custom-built board from Stresing Entwicklungsbüro.

Cross-check, comparison and fitting of PL and EL spectra

To study and confirm the source of the difference between PL and EL. The cross-check was performed carefully by different spectrometers (Labsphere CDS 610 fiber spectrometer and Edinburgh Instruments FLS980 Spectrometer). Fittings of PL and EL were performed by Origin 2019 software based on the Gauss equation. Parameters of PL and EL in CIE 1931 Chromaticity Coordinate were calculated by software CIE1931xy.V.1.6.0.2

Stability test of LEDs

All stability tests of LEDs were performed on the same setup for the characterization of LED performance. All $MAPbI_3$ stability tests of NP LEDs were performed in the Optoelectronics Group of Cavendish Laboratory, University of Cambridge. The stability tests were performed on fixed current densities supported by a source measure unit (Keithley K2400). T_{50} is the defined time that the original luminance is halved. The photon flux was collected by a calibrated silicon photodiode. The luminance and voltage were recorded by software simultaneously. The EL spectra of LEDs were tested immediately after the stability test without turning off the devices to avoid the possible recovery procedure. The EQE, J-V and luminance-current density were performed before and after the stability test. The lifetime of LEDs by different alcohols and under different current densities was calculated based on 3 devices at least.

Definition of "flash-evaporation" in FEPS method

$$\frac{dP}{dT} = \frac{L}{T\Delta V} = \frac{\Delta S}{\Delta V} \quad \text{or} \quad ln\frac{P_2}{P_1} = \frac{L}{R}\left(\frac{1}{T_1} - \frac{1}{T_2}\right)$$

Clausius–Clapeyron relation gives the pressure–temperature (P–T) relation. P is pressure, T is the temperature, L is the specific latent heat, ΔV is the volume change of evaporation and ΔS is the evaporation entropy of polar solvents. The boiling point is lower under lower pressure. The pressure of the system for FEPS can reach 1 bar (100 Pa), and the boiling point of polar solvents decreases dramatically. It can be degassed quickly, that is so-called flash evaporation.

State of art perovskite LEDs

As shown in Table S1, the highest EQE based on quasi-2D/3D $MAPbI_3$ or its NPs, to the best of our knowledge, is 17% and shows EL emission at the infrared range. This work demonstrates the EQE of 26.8%. To be noticeable, this record efficiency was achieved without any additional surface treatment or ligand exchange. The LEDs show sharp EL with FWHM of 29-43 nm, among which the FWHM of the most efficient LEDs prepared n-BuOH and $EtOH_{(n = 5)}$ is 43 and 42 nm, correspondingly, which is much sharper than the traditional bulk quasi-2D perovskite LEDs (61 nm). The EQE of the most efficient n-BuOH LEDs reaches peak between 0.0073-0.01859 $mA \cdot cm^{-2}$ with luminance of 1.5-3.7 $cd \cdot m^{-2}$, it remains >25% at ~0.02 $mA \cdot cm^{-2}$ with luminance of ~4 $cd \cdot m^{-2}$, >20% at ~0.1-0.2 $mA \cdot cm^{-2}$ with luminance of ~20 $cd \cdot m^{-2}$ and >10% at ~3 $mA \cdot cm^{-2}$ with luminance of ~250 $cd \cdot m^{-2}$. For the most efficient $EtOH_{(n = 5)}$ LEDs, the EQE reaches peak between 0.00377-0.02909 $mA \cdot cm^{-2}$ with luminance of 0.3-2.3 $cd \cdot m^{-2}$, it remains >25% at ~0.02-0.03 $mA \cdot cm^{-2}$ with luminance of ~2-3 $cd \cdot m^{-2}$, >20% at ~0.2-0.4 $mA \cdot cm^{-2}$ with luminance of ~10-25 $cd \cdot m^{-2}$ and >10% at ~3-8 $mA \cdot cm^{-2}$ with luminance of ~100-260 $cd \cdot m^{-2}$. These data are high enough to get accurate EQE. The maximum luminance of LEDs prepared by n-BuOH has been further improved to 4.33×10^3 at ~120 $mA \cdot cm^{-2}$ with a peak EQE of 21.1% (Fig. S26). The longest lifetime (T_{50}) of 267 min at 1.0 $mA \cdot cm^{-2}$ was achieved, that is a pretty good result considering these LEDs are based on $MAPbI_3$ rather than inorganic $CsPbI_3$, $MAPbI_3$ is generally thought to be less stable because of volatile organic MA component.

Table S1. Efficient perovskite LEDs published recently.

Perovskite Type	EL_{peak} (nm)	FWHM (nm)	EQE_{Peak} (%)	L_{max} ($cd{\cdot}m^{-2}$)	Operational lifetime (T_{50})	References
			**Green**			
3D $MAPbBr_3$	~540	/	8.53	/	/	(69)
3D $CsPbBr_3$:PEABr:PEG	~514	~21	20.3	~2 × 10^4	/	(70)
7 nm $CsPbBr_3$ QDs	~500	/	22	~5 × 10^5	60 min @ 1,200 $cd{\cdot}m^{-2}$	(23)
Quasi-2D $PEAMAPbBr_3$	~520	22	20.4	8.2 × 10^4	6.5 min @ 10,000 $cd{\cdot}m^{-2}$	(71)
$FA_{1-x}GA_xPbBr_3$ QDs	531	~24	23.4	~2.4 × 10^4	132 min @ 100 $cd{\cdot}m^{-2}$	(5)
Quasi-2D crown:MPEG-MAA-PEABr:$CsPbBr_3$	514	~32	28.1	3.7 × 10^4	4.04 h @ 100 $cd{\cdot}m^{-2}$	(56)
Quasi-2D TFPPO-$PEA_2Cs_{1.6}MA_{0.4}Pb_3Br_{10}$	517	20	25.6	~5 × 10^4	115 min @ 7,200 $cd{\cdot}m^{-2}$	(36)
Bulk $(FA_{0.7}MA_{0.1}GA_{0.2})_{0.87}Cs_{0.13}PbBr_3$ QDs in 3D solid	540	~22	28.9	4.7 × 10^5	520 h @ 1,000 $cd{\cdot}m^{-2}$	(14)
FAGAPbBr	530	20	23.26	~1.0 × 10^4	56-81 min @ 100 $cd{\cdot}m^{-2}$	(15)
PEA-$CsPbBr_3$ NCs	530	/	25.32	1.3 × 10^5	174 min @ 3000 $cd{\cdot}m^{-2}$	(21)
Quasi-2D CsBAPbBr	511	20	24.5	8 × 10^4	16 min @ 500 $cd{\cdot}m^{-2}$ T_{80}	(72)
Bulk $CsMAPbBr_3$	529	/	22.06	1.0 × 10^5	/	(73)
$CsPbBr_3$ NCs	514	20	26.7	~5 × 10^3	3h @ 1 $mA{\cdot}cm^{-2}$	(74)
Quasi-2D DBPF-OK-PEAMAFAPbBr	528	/	23.2	~1.0 × 10^4	~210 min @ 100 $cd{\cdot}m^{-2}$	(75)
$FAPbBr_3$ NCs	536	20	22.5	5 × 10^4	45.6 h @ 100 $cd{\cdot}m^{-2}$ in N_2	(76)
$CsPbBr_3$ NCs	520	21	22.5	~1.0 × 10^4	/	(77)
Quasi-2D CsFAPEAPbBr	515	/	26.0	8.4 × 10^4	/	(78)
Quasi-2D BACsGAPbBr	512	/	20.1	1.0 × 10^5	15.3 h @ 100 $cd{\cdot}m^{-2}$	(79)
Bulk CsRbPbBr film	512	/	26.09	1.2 × 10^4	/	(80)
Quasi-2D PEAMAFAPbClBr film	530	24	29.5	1.5 × 10^5	18.7 h @ 12,000 $cd{\cdot}m^{-2}$	(81)
Quasi-2D CsBAPEAPbBr film	532	/	26.2	>1 × 10^5	920 min @ 1000 cd m^{-2}	(82)
Bulk $CsMAFAPbBr_3$	529	/	24.6	~8 × 10^3	23.8 min	(83)
Bulk $Cs_xFA_{1.3-x}PbBr_3$ film	528	17	21.3	~4.6 × 10^5	~125 h at 1,000 $cd{\cdot}m^{-2}$	(84)
Bulk $CsPEALiPbBr_3$ film	516	24	32.1	1.1 × 10^4	3.56 h @ 100 $cd{\cdot}m^{-2}$	(85)
Tandem organic + bulk $FAGAPbBr_3$	529	27.3	37.0	~1 × 10^4	5,596 h @ 100 $cd{\cdot}m^{-2}$	(86)
			**Red**			
$CsPb(Br/I)_3$ QDs	649	33	21.3	7.9 × 10^2	180 min @ 100 $cd{\cdot}m^{-2}$	(16)
$MAPb(I_xBr_{1-x})_3$ QDs	620	~55	20.3	6.3 × 10^2	130 min @ 1 $mA{\cdot}cm^{-2}$	(7)
$CsPbI_3$ QDs	640	31	23	~1 × 10^3	10 h @ 200 $cd{\cdot}m^{-2}$	(22)
$Cs_4PbI_6/FA_xCs_{1-x}PbI_3$ (0D/3D) heterostructure	705	34	21.0	1.4 × 10^3	/	(87)
$CsPb(I_xBr_{1-x})_3$ QDs	~650	30, 42	24.4	~2.9 × 10^2	20 h @ 290 $cd{\cdot}m^{-2}$	(34)
$CsPbI_3$ QDs	620-650	34-47	20.8	3.8 × 10^3	7 min @ 110 $cd{\cdot}m^{-2}$	(13)
Quasi-2D $((PEA/m\text{-}F\text{-}PEA)_xNMA_{1-x})_2CsPb_2I_7$	680	34, 43	25.8	1.3 × 10^3	40 min @ 100 $cd{\cdot}m^{-2}$	(33)
Bulk $CsPbI_3$ QDs in solid	679	~33	20.8	~1.1 × 10^3	375 min @ 100 $cd{\cdot}m^{-2}$	(8)
$CsPbI_3$ NCs	681	35	20.65	3.9 × 10^3	128 min @ 150 $cd{\cdot}m^{-2}$	(12)

Quasi-2D $(PPT)_2FA_2Pb_3I_{10}$	~700	~65	26.3	~3 × 10^2	2.8 h @ 100 cd·m^{-2}	(35)
$CsPbBr_xI_{3-x}$ QDs	~640	~35	16.7	1.4 × 10^4	/	(88)
Quasi-2D LFAPb(Br/I)	679-724	/	26.2	~1.2 × 10^4	/	(41)
$CsPbI_3$ NCs	656	33	~23	1.3 × 10^3	489 min@100 cd·m^{-2}	(11)
$CsPb(Br/I)_3$ NCs	635	33	22.8	1.3 × 10^4	63 min @ 100 cd·m^{-2}	(18)
$CsPb(Br/I)_3$ NCs	630-640	/	23.5	1.5 × 10^3	97 min @ 100 cd·m^{-2}	(10)
$CsPbBrI_2$ NCs	646 - 676	49	23.6	~2 × 10^3	~2500 min	(29)
$CsPbI_3$ QDs	638	36	26.1	2.5 × 10^3	7.5 h @ 100 cd·m^{-2}	(24)
$CsPbI_3$ QDs	703	29	18.8	8.0 × 10^3	33.6 h @ 100 mA·cm^{-2}	(25)
$CsPbI_3$ QDs	644	/	28.5	4.1 × 10^3	30 h @ 100 cd·m^{-2}	(26)
AnHI/TMSBr treated $CsPbBr_xI_{3-x}$ QDs	636	28	>22	7 × 10^3	780 h, T_{90} @ 100 cd·m^{-2}	(28)
$CsPb(Br/I)_3$ NCs	~640	32	23.0	2.9 × 10^3	/	(27)
Bulk $CsPbBr_x/I_{3-x}$ films	636	27	~21	1.6 × 10^3	240 min @ 130 cd·m^{-2}	(89)
Bulk CsMBAMPPAPbI	620-650	/	28.7	~2 × 10^3	7,600 min @ 100 cd·m^{-2}	(90)
**$MAPbI_3$ NPs**	**607**<br>**638**<br>**669**<br>**728**	**29**<br>**43**<br>**42**<br>**43**	**11.3**<br>**26.7**<br>**26.8**<br>**13.6**	**4.62 × 10^3**<br>**4.33 × 10^3**<br>**1.50 × 10^3**<br>**6.03 × 10^2**	**11-267 min @ 1.0 mA·cm^{-2} (~100 cd·m^{-2})**	**This work**
			**Infrared**			
				W·sr^{-1}·m^{-2}		
3D PImI:$MAPbI_3$	752	~42	15.6	~1 × 10^2	2 h @ 10 mA·cm^{-2}	(91)
3D PMA: $MAPbI_3$	~750	~47	17	2.6 × 10^3	117 min @ 10 mA·cm^{-2}	(92)
3D ODEA:$FAPbI_3$	~800	~49	21.6	3.1 × 10^2	20 h @ 25 mA·cm^{-2}	(93)
3D AEAA:$FAPbI_3$	~800	/	22.2	2.5 × 10^2	18.6 h @ 100 mA·cm^{-2}	(94)
3D $FAPbI_3$-MSPE	~800	~45	23.8	5.0 × 10^2	32 h @ 100 mA·cm^{-2}	(95)
3D $FAPbI_3$	~800	/	23.6	9.6× 10^2	106.1 @ 100 mA·cm^{-2}	(96)
$CsSnI_3$	948	71	/	226	39.5 h (100 mA cm^{-2})	(97)
3D $FAPbI_3$	~800	/	32.0	3.9 × 10^2	19 h @ 100 mA·cm^{-2}	(98)

State of the art synthesis methods for perovskite NPs

Ligand-assisted reprecipitation (LARP) and hot-injection are widely used approaches for the synthesis of perovskite nano materials (NMs). LARP is performed at room temperature in the air in which the long chain alkyl ammonium is used as ligand and dipping N,N-Dimethylformamide (DMF) solutions of precursors (e.g. MABr, $PbBr_2$) into antisolvent such as toluene, hexane, etc. However, the presence of DMF used in this method will result in the dissolution/decomposition of the perovskite NPs formed to convert back to precursors. Hot injection or similar modified methods are widely used for the synthesis of the ABX_3 NPs family. Generally, pre-synthesized A-site cation oleate (AOA, e.g. cesium oleate (CsOA), methylammonium oleate (MAOA)) is injected into the lead halide in the presence of oleic acid (OA), oil amine (OM), octadecene (ODE). The mole ratio of lead: halide is 1:2 in the lead halide precursor solution, the AOA doesn't introduce any further halide. It means the precursor ratio of Cs: Pb: halide can't be the ideal composition of perovskite which is 1:1:3. It will cause a lack of halide and formation of by-product $Pb(OA)_2$ which often separates and be observed in the obtained crude NP solutions. The functionality of perovskite NPs is extremely sensitive to the nature of the surface. This by-product can contaminate the surface and deteriorate the performance of perovskite NP devices. In contrast, injection of A-site cation halide (AX) polar solvent (e.g., H_2O, alcohols) solution into the lead halide (PbX_2) precursor solution in the FEPS method introduces the A-site cation and equivalent halide, which makes the ratio of A: Pb: halide in precursors to be ideal 1:1:3. This method also obviates the need of pre-synthesized precursors such as CsOA, MAOA in traditional hot-injection method. The yield of the targeted $MAPbI_3$ NPs for LED preparation is ~30-60% in this FEPS method. Furthermore, the n-phase distribution of perovskite NPs has been proved to be modulated by applying polar solvents with different polarity in this FEPS method, which is the dominating factor and prerequisite of this tailored ALN EL emission. There is an important step "flash-evaporation of polar solvent". This method first proposed a concept of flash-evaporating polar solvents for nanomaterials synthesis to the best of our knowledge, which is much simplified, widely applicable with excellent performance. It is highly repeatable and easy to learn and has been repeated more than 80 times both at Peking University and the University of Cambridge by different co-authors.

Table S2. Some representative methods to prepare and control the size/thickness of perovskite and methods for NPs synthesis using polar solvents such as H_2O and alcohols.

Method	Precursor + Solvent	condition	Applicable to	Size control method	References.
**Hot injection**	PbX_2 , AOA OA, OM, ODE	120-180 °C	ABX_3 with different AOA	Temperature, ligands, precursor ratio	(99) (100) (101)
**Ligand-assisted re-precipitation (LARP)**	$PbBr_2$, MABr DMF, anti-solvents	RT in air	$MAPbBr_3$	/	(102)
**Modified LARP**	AX, BX_2, LX DMF	RT	$APbX_3$, $ASnX_3$ with n = 1, 2, ∞	Precursor, ligands ratio	(103)
**Ligand-assisted ultrasonication**	Cs_2CO_3, PbX_2 OM, OA	ultrasonicat ion	$CsPbX_3$	None	(99)
**Water-induced $MAPbBr_3$@PbBr(OH)**	$PbBr_2$, MABr, DMF, H_2O	70 °C	$MAPbBr_3$@PbBr(O H)	None	(104)
**Polar alcohols for $CsPbBr_3$ Nanorods**	CsBr, $PbBr_2$ OA, OM, butanol	ultrasonicat ion	$CsPbBr_3$	None	(105)
**H_2O-DMF-DMSO Solvent Synthesis**	$PbBr_2$, CsBr, H_2O, DMF, DMSO	80 °C, oil bath	Cs_4PbBr_6	None	(106)
**Polar solvent controlled ionization (PCI) method**	CsOA, $Pb(OA)_2$, AX, IPA, hexane	RT	$CsPbX_3$ nanorods, nanoparticles, nanowires	None	(107)
**LARP with water**	CsBr, $PbBr_2$, DMF, toluene, 30 μL H_2O	RT	$CsPbBr_3$	Water	(108)
**This work FEPS**	PbX_2, AX, alcohols, H_2O, OA, OM, ODE	70-120 °C vacuum	All ABX_3	Solvents with different polarity, postprocessing	

Details of NMs synthesis

Table S3. The detailed synthesis condition of $APbX_3$ perovskite NMs by FEPS method and cesium oleate (CsOA) method. EtOAc: ethyl acetate.

NPs	OA (mL)	OM (mL)	ODE (mL)	PbX_2 (mg)	AX (mg)	AX solutions and injection condition	Details of centrifugation and wash solvents
$CsPbCl_3$	1.0	2.0	15	278	CsCl 168	0.5 mL H_2O + 0.5 mL MeOH 120 °C	14000 $r \cdot min^{-1}$ 10 mL EtOAc
$CsPbBr_3$	1.2	1.5	15	367	CsBr 213	0.5 mL H_2O + 0.5 mL MeOH 120 °C	14000 $r \cdot min^{-1}$ 10 mL EtOAc
$CsPbI_3$	1.4	1.4	15	461	CsI 260	0.5 mL H_2O + 0.5 mL MeOH 120 °C	14000 $r \cdot min^{-1}$ 10 mL toluene
$MAPbCl_3$	1.0	2.0	15	278	MACl 67.5	1.4 mL EtOH 90 °C	14000 $r \cdot min^{-1}$ 10 mL EtOAc
$MAPbBr_3$	1.2	1.5	15	367	MABr 112	1.0 mL EtOH 90 °C	14000 $r \cdot min^{-1}$ 10 mL EtOAc
$MAPbI_3$	1.5	1.5	15	461	MAI 157	1.0 mL MeOH 70 °C	14000 $r \cdot min^{-1}$ 10 mL EtOAc
$MAPbI_3$	1.5	1.5	15	461	MAI 157	1.5 mL EtOH 70 °C	14000 $r \cdot min^{-1}$ 10 mL EtOAc
$MAPbI_3$	1.5	1.5	15	461	MAI 157	2.0 mL IPA 70 °C	14000 $r \cdot min^{-1}$ 10 mL EtOAc
$MAPbI_3$	1.5	1.5	15	461	MAI 157	3.0 mL n-BuOH 70 °C	14000 $r \cdot min^{-1}$ 10 mL EtOAc
$FAPbCl_3$	1.0	1.5	15	278	FACl 80.4	1.0 mL EtOH 90 °C	14000 $r \cdot min^{-1}$ 10 mL EtOAc
$FAPbBr_3$	1.2	1.5	15	367	FABr 125	1.0 mL EtOH 90 °C	14000 $r \cdot min^{-1}$ 10 mL EtOAc
$FAPbI_3$	1.5	1.5	15	461	FAI 172	1.0 mL EtOH 70 °C	14000 $r \cdot min^{-1}$ 10 mL toluene

As transmission electron microscopy (TEM) images show in Fig. S8, the broad range of typical $APbX_3$ perovskite nanomaterials (NMs, A = Cs^+, MA^+, FA^+; X = Cl^-, Br^-, I^-) were successfully obtained by this method. The $CsPbX_3$ series are uniform in size and with a cube shape, while $MAPbX_3$, and $FAPbX_3$ series are plate-shape. Solutions of $APbX_3$ NMs show sharp photoluminescence (PL) which covers from deep blue to infrared region. The PL peak and full width at half maximum (FWHM) of the $CsPbX_3$ series are 407 nm/10 nm ($CsPbCl_3$), 506 nm/15 nm ($CsPbBr_3$), 692 nm/33 nm ($CsPbI_3$), respectively. The PL peaks of $MAPbBr_3$ (519 nm, 29 nm) and $FAPbBr_3$ (532 nm, 33 nm) showed a gradual shift to longer wavelength with the increasing size of Cs^+, MA^+ and FA^+ compared to $CsPbBr_3$. $MAPbI_3$ NPs by MeOH show a sharp peak at the edge of the orange/red range with a short wavelength (615 nm, 30 nm), which is visually brighter. PL of $FAPbI_3$ (767 nm, 56 nm) red-shifts to the infrared region. The perovskite NMs obtained by this method achieved high PLQE, among which the highest one reaches 99% in $Cs_xFA_{1-x}PbI_3$, which is close to the theoretical limit. The PLQE of the $APbI_3$ series by this method is generally high, 72-91%, the PLQE of the $APbBr_3$ series is slightly lower, 66-87%, the PLQE of $APbCl_3$ series NMs is low, and the PLQE of $MAPbCl_3$ and $FAPbCl_3$ is very low, about 4%. All of the $CsPbX_3$, $MAPbX_3$, and $FAPbX_3$ series perovskite NMs show a large color gamut which is much larger (>160%) than the Standard Red Green Blue (sRGB) color space from International Electrotechnical Commission (IEC).

Table S4. Physicochemical properties of solvents used in this project (*49*).

Solvent	Dielectric Constant (ε_r)	Boiling Point (°C)	Relative polarity
**H_2O**	80.10	100.0	1.000
**MeOH**	32.70	64.7	0.762
**EtOH**	24.55	78.3	0.654
**n-BuOH**	18.77	117.6	0.586
**IPA**	19.92	82.3	0.546
**toluene**	2.38	110.6	0.099

Charge/energy transfer in NP solutions/films and quasi-2D films

There are 3 possible charge/energy routes including charge transfer (CT), non-radiative Förster resonance energy transfer (FRET) and radiative absorption-reemission (AR) energy transfer process in NP films/solutions and quasi-2D film under optical/electrical excitations. Among these, the charge transfer majorly depends on the conductivity of materials, the FRET efficiency is determined by donor-acceptor distance and the spectral overlap, and the AR largely relies on the absorptance of smaller bandgap emitters. The AR could be excluded firstly because of the limited thickness of emitter layers (~10 nm) makes the large ALN emitters cannot sufficiently absorb the photons from small n in NP film/LEDs under both optical and electrical excitations.

1. Non-radiative Förster resonance energy transfer (FRET):

$$k_{ET} = \frac{1}{\tau_D}\frac{1}{R^6}\left[\frac{3}{4\pi}\int \frac{c^4}{\omega^4\varepsilon^4}F_D(\omega)\sigma_A(\omega)d\omega\right]$$

τ_D is the lifetime of small n excited states, R is the distance between small and large n NP emitters, c is the light speed, ω is the angular frequency of emission light, ε is the dielectric constant of materials, F_D is the fluorescence spectrum of small n, and σ_A is the absorption spectrum of large n.

2. Radiative absorption-reemission (AR), Beer-Lambert law:

$$\mathrm{A} = \log_{10}\frac{T_t}{T_0} = K \cdot \mathrm{l} \cdot \mathrm{c}$$

The K (mol·cm^{-2}) is the absorptance coefficient, l is the length (cm), c is the concentration of perovskite. The density of $MAPbI_3$ is 4.1 g·cm^{-3}.

3. Charge transfer (CT)

The rate of charge transfer (CT) can be estimated by the conductivity of the NP films, which is inversely correlated with the amount of ligands (L/Pb) on the surface. The conductivity of NP films is between 0.58-2.24 × 10^{-8} S·cm^{-1}, which was much smaller comparing with 9.41 × 10^{-8} S·cm^{-1} and 4.57 × 10^{-7} S·cm^{-1} of the bulk quasi-2D and 3D $MAPbI_3$ films.

The results of UV-Vis (Fig. 4C, Fig. S23) demonstrate 10 nm $MAPbI_3$ film can only absorb <1% photons at n = 4 (640 nm) and n = 5 (670 nm) wavelength, so the AR could be excluded firstly in the NP film/LEDs under both optical and electrical excitation. NP solutions in 1.0 cm thick cuvette can absorb 95% photons at n = 4 (640 nm) even in 1 mg·mL^{-1} NP solutions, the AR process can't be ignored. Obvious differences in PL spectra could be observed in NP solutions with different concentrations (Figs. S11-15). The ratio of PL components from small n-value NPs increased and the [n] in PL shifted 2-3 to small value when the crude solutions were diluted. Small n = 1-2 components could be observed in diluted crude solutions (Fig. S12). The major PL peaks of NP solutions prepared by MeOH blue-shifted from 650 nm (n = 4) to 614 nm (n = 3) when solutions were diluted from 28 to 0.055 mg·mL^{-1}, n = 3 peak appeared when the solutions were diluted to low concentration (< 0.22 mg·mL^{-1}) (Fig. S14). This phenomenon indicates that more emission from small n-value NPs is absorbed and then re-emitted by large n-value NPs in concentrated solutions. The average distance of NPs in solutions increases from ~30 to ~300 nm when the NPs are diluted from 28 to 0.055 mg·mL^{-1} (Fig. S14C). This distance is much larger than the effective one for both FRET (up to 10-12 nm) and CT, so these two routes can be excluded in NP solutions. All of these results demonstrate that AR is the dominating process in NP solutions.

As shown in Fig. 2G-I, Fig. 4K and Fig. S21, the [n] in EL slightly shifts to a larger value but the [n] in PL largely shifts 2-3 to a larger value than the real [n] via AFM. A much higher ratio of photons emits from small n NPs in EL, making the EL of these NP LEDs always blue-shifted (the largest, 89 nm). The shift to larger [n] in PL of NP films can be ascribed to FRET which happened in the picosecond time range as proved by the TA results. The TA results (Fig. S29) showed that the early-time GSB of n = 2 NPs is strong in both NP films prepared by EtOH, IPA and n-BuOH. However, no obvious peak from n = 2 NPs could be observed from both their PL and EL. It proves that energy transfer from n = 2 NPs to larger NPs (n = 4-5) occurs immediately in the sub-picosecond time scale. Both the fast, intermediate and slow stages in NP solutions (Fig. S30) are even much slower than the ones in quasi-2D films because the energy transfer via FRET and charge transfer are hindered by the much larger distance between NPs in solutions.

Furthermore, the excitation and the following charge/energy transfer processes are quite different in PL and EL. The energy spontaneously can aggregate towards the large n via the FRET process under photoexcitation, so the PL of both bulk quasi-2D and NP films are majorly from large n-phase. Electron

and hole inject separately from charge transfer layers under electro-excitation. The “separated” injection of hole and electron greatly reduces the possibility of energy transfer via FRET, so the contribution of FRET should be very minor in working LEDs, our results are consistent with previous reports. As proved in TrEL, the τ_d difference happened in the nanosecond rather than the picosecond time scale (the time scale of FRET is picosecond to sub-nanosecond). The [n] in EL relies on the conductivity of NP films. All of the systematic evidence proves that the CT is the major energy transfer route in working LEDs, but the CT was reduced in NP LEDs, so the EL of NP LEDs blue-shifts than its PL.

The contributions of the CT, FRET and AR in NP films/solutions and quasi-2D film under optical/electrical excitations can be summarized as follows.

	**FRET**	**CT**	**AR**
**Quasi-2D film (PL)**	major	moderate	minor
**Quasi-2D film (EL)**	minor	major	minor
**NP films (PL)**	major	minor	minor
**NP films (EL)**	minor	minor/moderate	minor
**NP solutions (PL)**	minor	minor	major

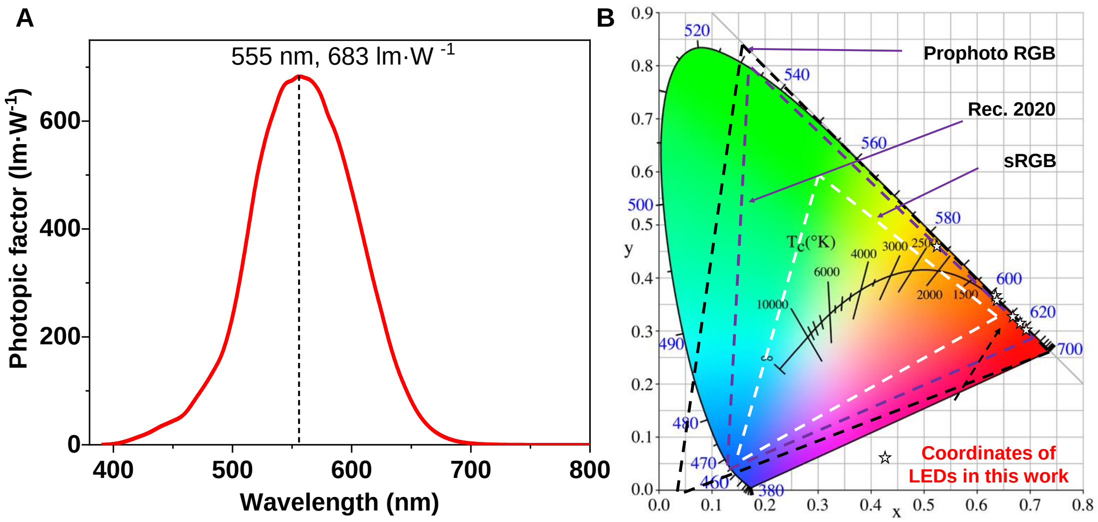

Fig. S1. The photopic curve and coordinates of LEDs in this work. (**A**) The photopic curve $P(\lambda)$ demonstrates the response of the human eye to light and is given in lm·W^{-1}, raw data are from the previous paper (68). (**B**) the Color gamut of different standards and coordinates of LEDs in this work. Standard RGB (red, green, blue) color space, IEC 61966-2-1:1999, Rec. 2020 and ProPhoto RGB.

The wavelength of the red primary color defined in the standard Rec. 2020 for ultra-high-definition television is 630 nm. Longer wavelength such as 700 nm can offer an especially large gamut, which is the ideal wavelength in standard ProPhoto RGB designed for use with photographic output.

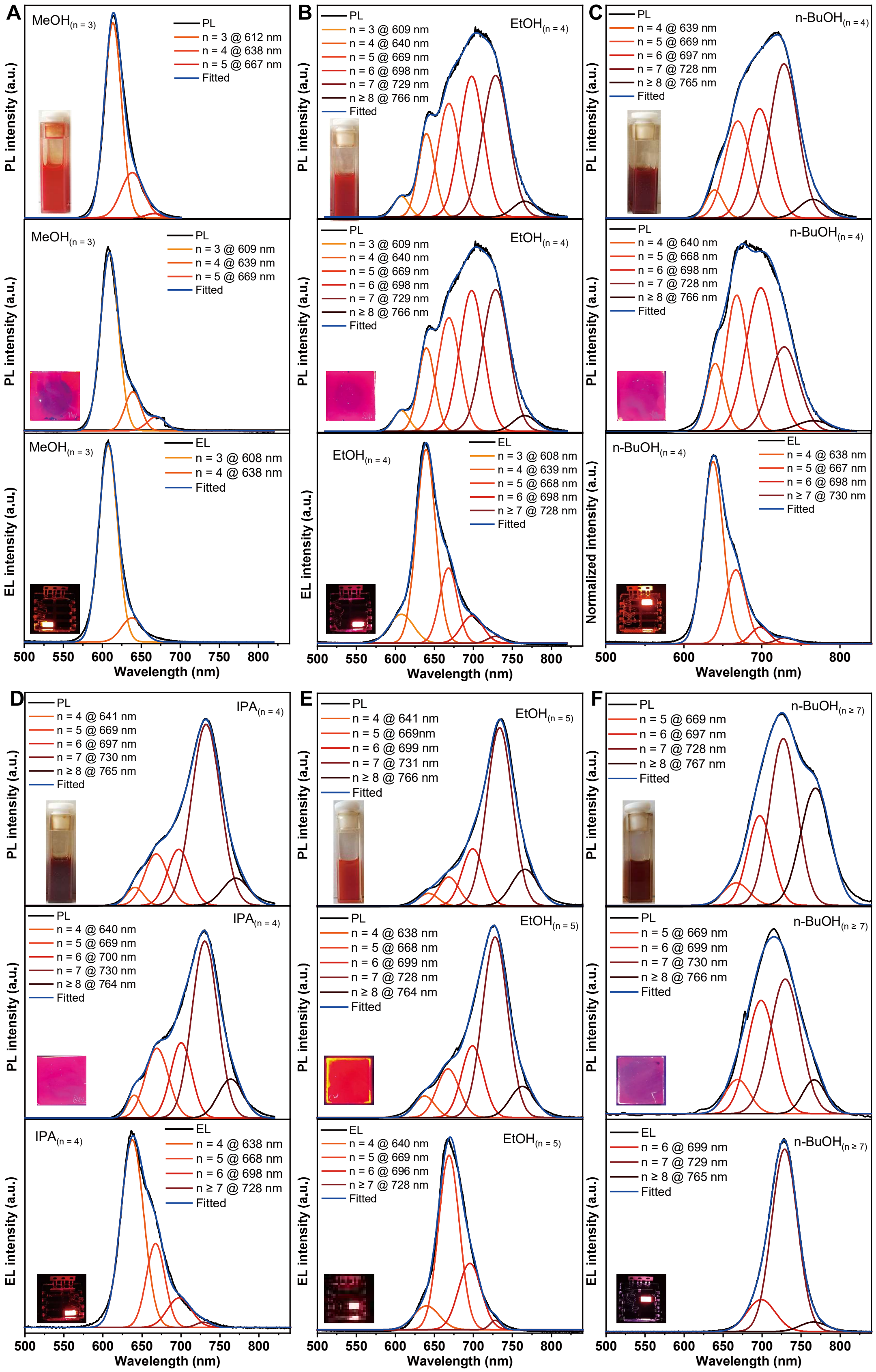

Fig. S2. PL and EL comparison. Fitting results of PL of $MAPbI_3$ NP solutions (2.0 mg·mL^{-1}) in a cuvette, films on glass and EL of LEDs (in Fig. 1C), prepared by (**A**) MeOH$_{(n=3)}$; (**B**) EtOH$_{(n=4)}$; (**C**) n-BuOH$_{(n=4)}$; (**D**) IPA$_{(n=4)}$; (**E**) EtOH$_{(n=5)}$; (**F**) n-BuOH$_{(n\geq7)}$. Inserted photos of films under UV light were prepared by spin-coating final $MAPbI_3$ NP solutions (2.0 mg·mL^{-1}) on glass. Photos of working LEDs were taken at 6 V.

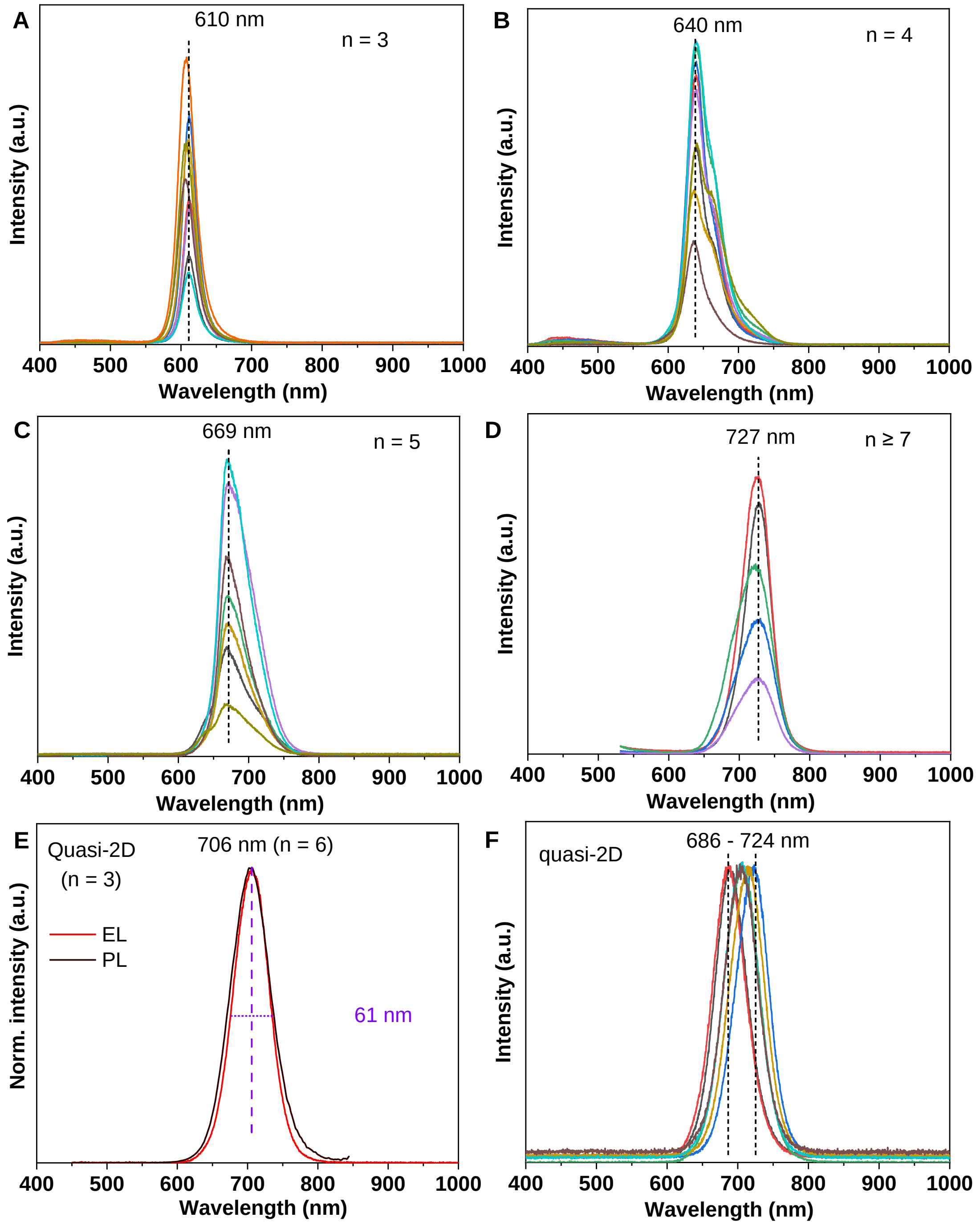

Fig. S3. EL spectra of different batches of NP LEDs. (**A**) n = 3 ALN emission prepared by MeOH; (**B**) n = 4 ALN emission prepared by IPA or n-BuOH; (**C**) n = 5 ALN emission prepared by $EtOH_{(n = 5)}$; (**D**) n ≥ 7 ALN emission prepared by $n\text{-}BuOH_{(n \geq 7)}$. (**E**) PL/EL of bulk quasi-2D (nominal n = 3) film/LEDs. (**F**) EL spectra of different batches of quasi-2D LEDs, fabricated from the precursor solutions with a stoichiometric ratio of nominal n = 3, $PEA_2MA_2Pb_3I_{10}$.

Cross section images of $MAPbI_3$ NP LEDs

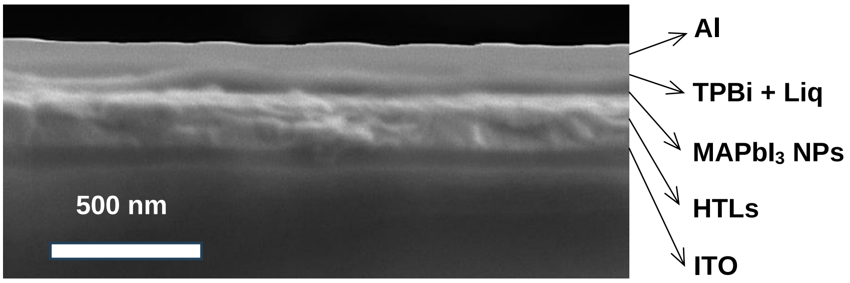

Fig. S4. Cross section image of $MAPbI_3$ NP LEDs. The thickness of each layer: ITO (~130 nm), HTLs (~100 nm), perovskite NPs (~10 nm), TPBi + Liq (~110 nm), Al (~100 nm).

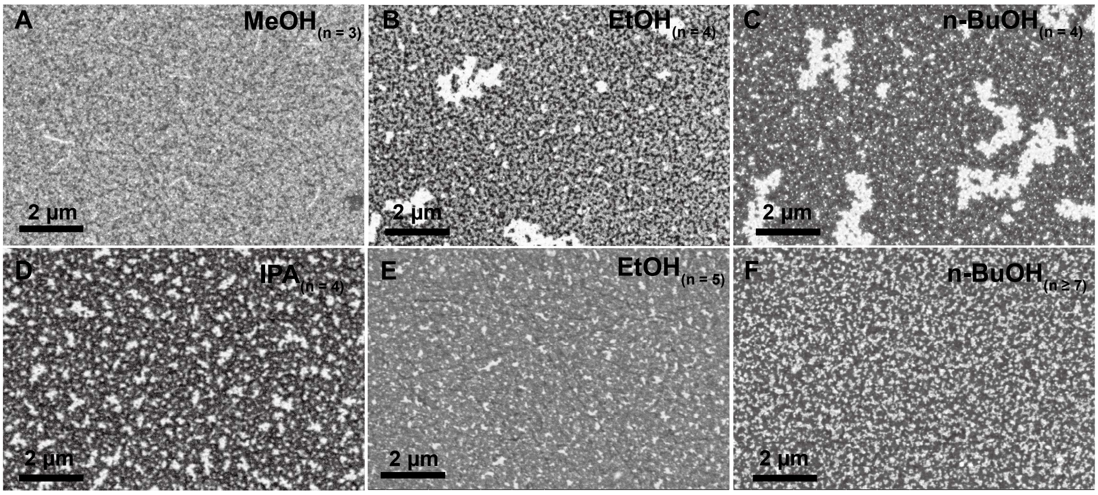

Fig. S5. SEM images of $MAPbI_3$ NP films. Prepared by (**A**) $MeOH_{(n = 3)}$, (**B**) $EtOH_{(n = 4)}$, (**C**) $n\text{-}BuOH_{(n = 4)}$, (**D**) $IPA_{(n = 4)}$, (**E**) $EtOH_{(n = 5)}$, (**F**) $n\text{-}BuOH_{(n \geq 7)}$, samples were prepared by spin-coating NP solutions on ITO/PEDOT:PSS/poly-TPD/TFB substrates.

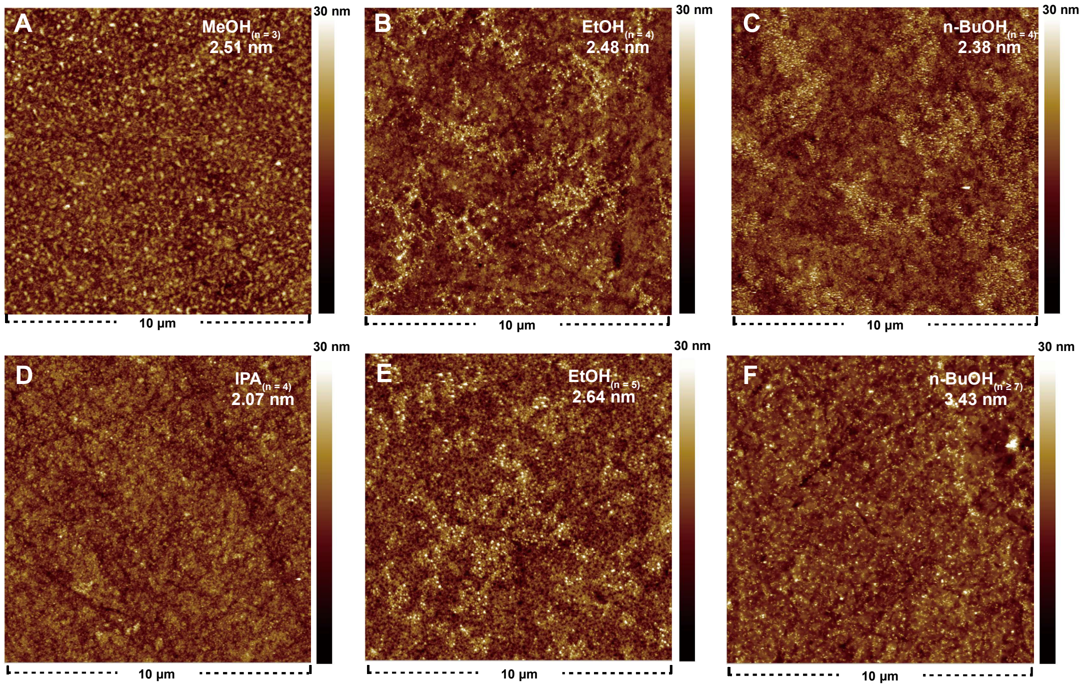

Fig. S6. AFM roughness of NP films. Prepared by (**A**) $MeOH_{(n = 3)}$ (2.51 nm). (**B**) $EtOH_{(n = 4)}$ (2.48 nm). (**C**) n-$BuOH_{(n = 4)}$ (2.38 nm). (**D**) $IPA_{(n = 4)}$ (2.07 nm). (**E**) $EtOH_{(n = 5)}$ (2.64 nm). (**F**) n-$BuOH_{(n \geq 7)}$ (3.43 nm), samples were prepared by spin-coating the final solutions (2.0 $mg \cdot mL^{-1}$) on ITO/PEDOT:PSS/poly-TPD/TFB substrates.

AFM/SEM images of quasi-2D perovskite films

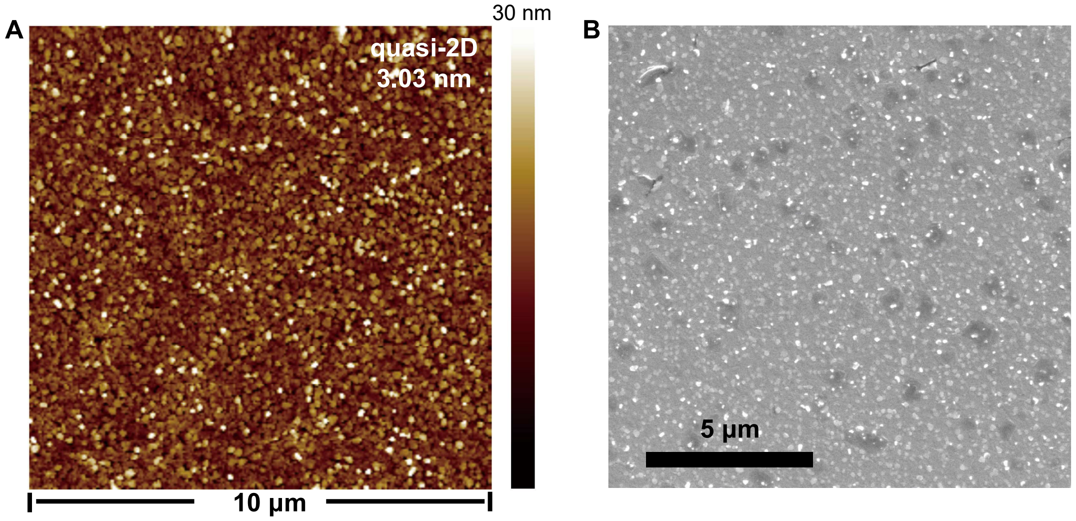

Fig. S7. Morphology of bulk quasi-2D film. (**A**) AFM image, roughness 3.03 nm. (**B**) SEM image. The sample was prepared by spin-coating quasi-2D precursor solution on ITO/PEDOT:PSS/poly-TPD/TFB substrates.

Luminescent property of $APbX_3$ NPs

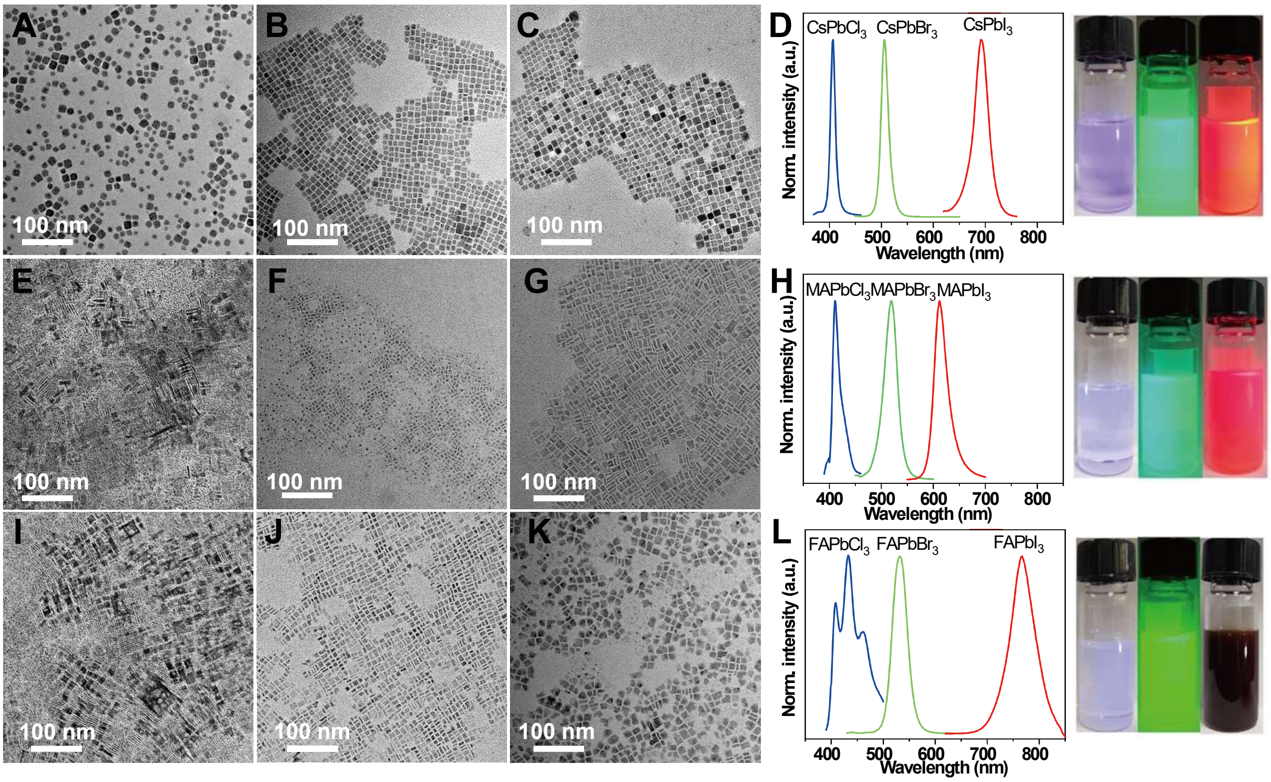

M

NPs	PL Peak (nm)	FWHM (nm)	PLQE (%)	CIE (x)	CIE (y)
$CsPbCl_3$	407	11	26	0.1693	0.0072
$CsPbBr_3$	506	15	70	0.0451	0.6637
$CsPbI_3$	692	33	72	0.7245	0.2755
$MAPbCl_3$	411	17	3.6	0.1679	0.0081
$MAPbBr_3$	519	29	66	0.1115	0.7001
$MAPbI_3$ (MeOH)	612	30	38	0.6627	0.3371
$FAPbCl_3$	433	/	3.0	0.1507	0.0336
$FAPbBr_3$	532	33	87	0.2043	0.7381
$FAPbI_3$	767	56	91	0.7327	0.2673
$Cs_xFA_{1-x}PbI_3$	597	31	99	0.6242	0.3751

N

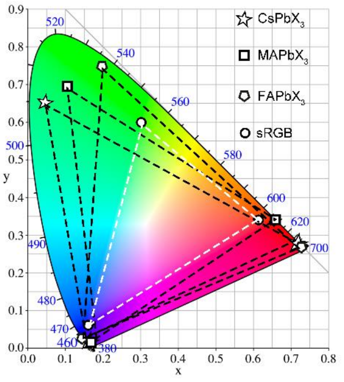

Fig. S8. TEM, PL and solution photos of ABX_3 perovskite NMs prepared by FEPS method. TEM of (**A**) $CsPbCl_3$, (**B**) $CsPbBr_3$, (**C**) $CsPbI_3$. (**D**) PL and photos of $CsPbX_3$ NP solutions**.** TEM of (**E**) $MAPbCl_3$, (**F**) $MAPbBr_3$, (**G**) $MAPbI_3$ (n-BuOH). (**H**) PL and photos $MAPbX_3$ solutions**.** TEM of (**I**) $FAPbCl_3$, (**J**) $FAPbBr_3$, (**K**) $FAPbI_3$. (**L**) PL and photos of $FAPbX_3$ solutions. (**M**) PL parameter summary of ABX_3 perovskite NMs. (**N**) Color gamut of perovskite NMs and sRGB in the International Commission on Illumination (CIE) 1931 chromaticity diagram. FA, formamidinium.

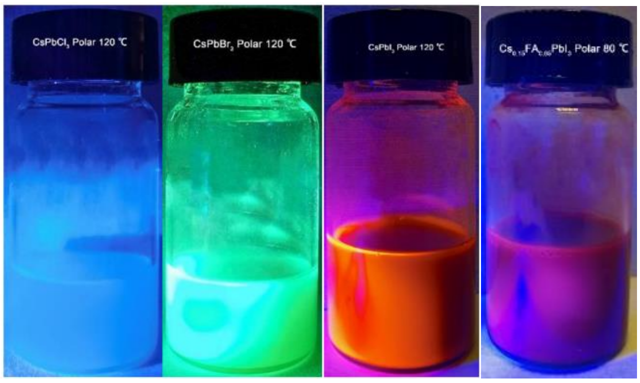

Fig. S9. Photos of $CsPbCl_3$, $CsPbBr_3$, $CsPbI_3$, $Cs_xFA_{1-x}PbI_3$ NM solutions prepared by FEPS method. Mixed A-cations or mixed halide NMs were obtained from mixed AX or PbX_2 precursors. Dispersed in toluene.

TEM images of $MAPbI_3$ NPs

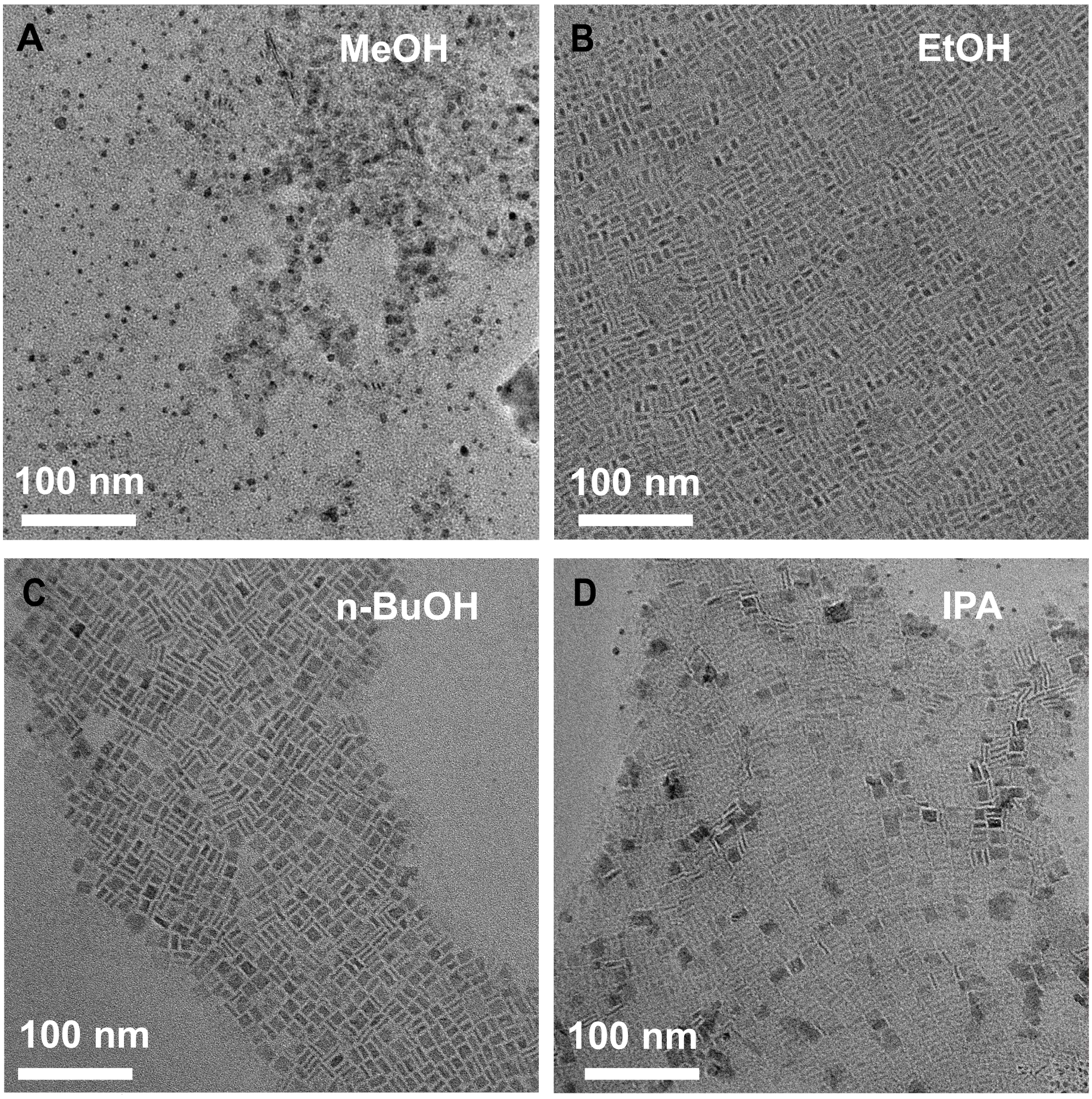

Fig. S10. TEM images of $MAPbI_3$ NPs prepared by different alcohols. (**A**) MeOH. (**B**) EtOH, (**C**) n-BuOH. (**D**) IPA. Samples were prepared by dropping the final solutions (2.0 $mg \cdot mL^{-1}$) on copper grids with carbon films.

Luminescent property of $MAPbI_3$ NPs

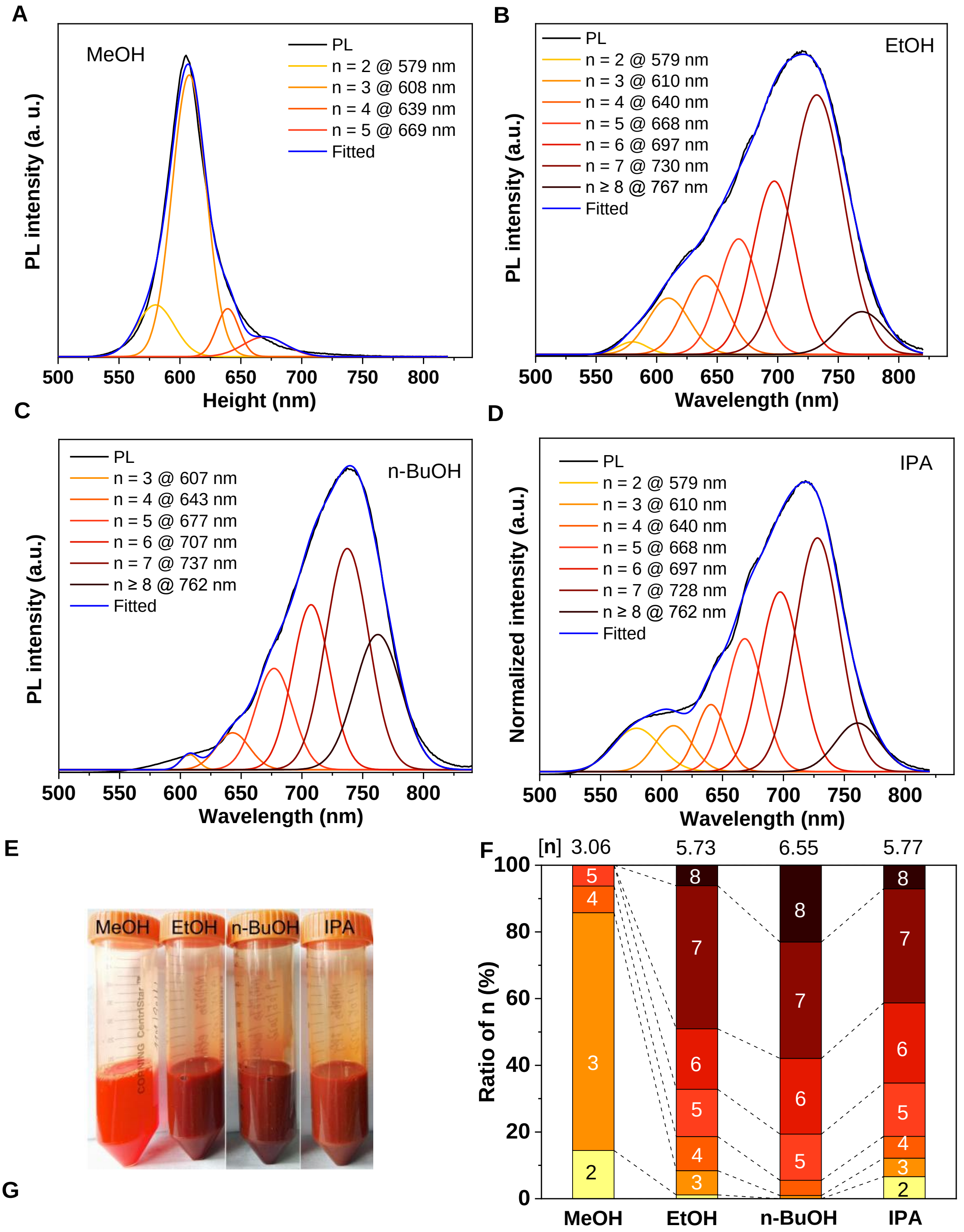

n	1	2	3	4	5	6	7	≥ 8	[n]
MeOH	0.0	14.5	71.3	8.0	6.2	0.0	0.0	0.0	3.06
EtOH	0.0	1.1	7.0	9.9	13.7	17.5	41.5	5.9	5.73
n-BuOH	0.0	0.0	1.0	4.5	13.9	22.7	34.9	23.0	6.55
IPA	0.0	6.6	5.6	6.5	16.0	24.0	34.2	7.1	5.77

Fig. S11. PL and multiple peaks fitting results of $MAPbI_3$ NPs crude solutions without dilution or other treatments. Prepared by (**A**) MeOH; (**B**) EtOH; (**C**) n-BuOH; (**D**) IPA. (**E**) Photos of crude solutions (left to right: MeOH, EtOH, n-BuOH, IPA). (**F**) the 2D graph of PL intensity ratio (%) from multiple peaks fitting results. (**G**) The ratio (%) of different phases from the fitting results.

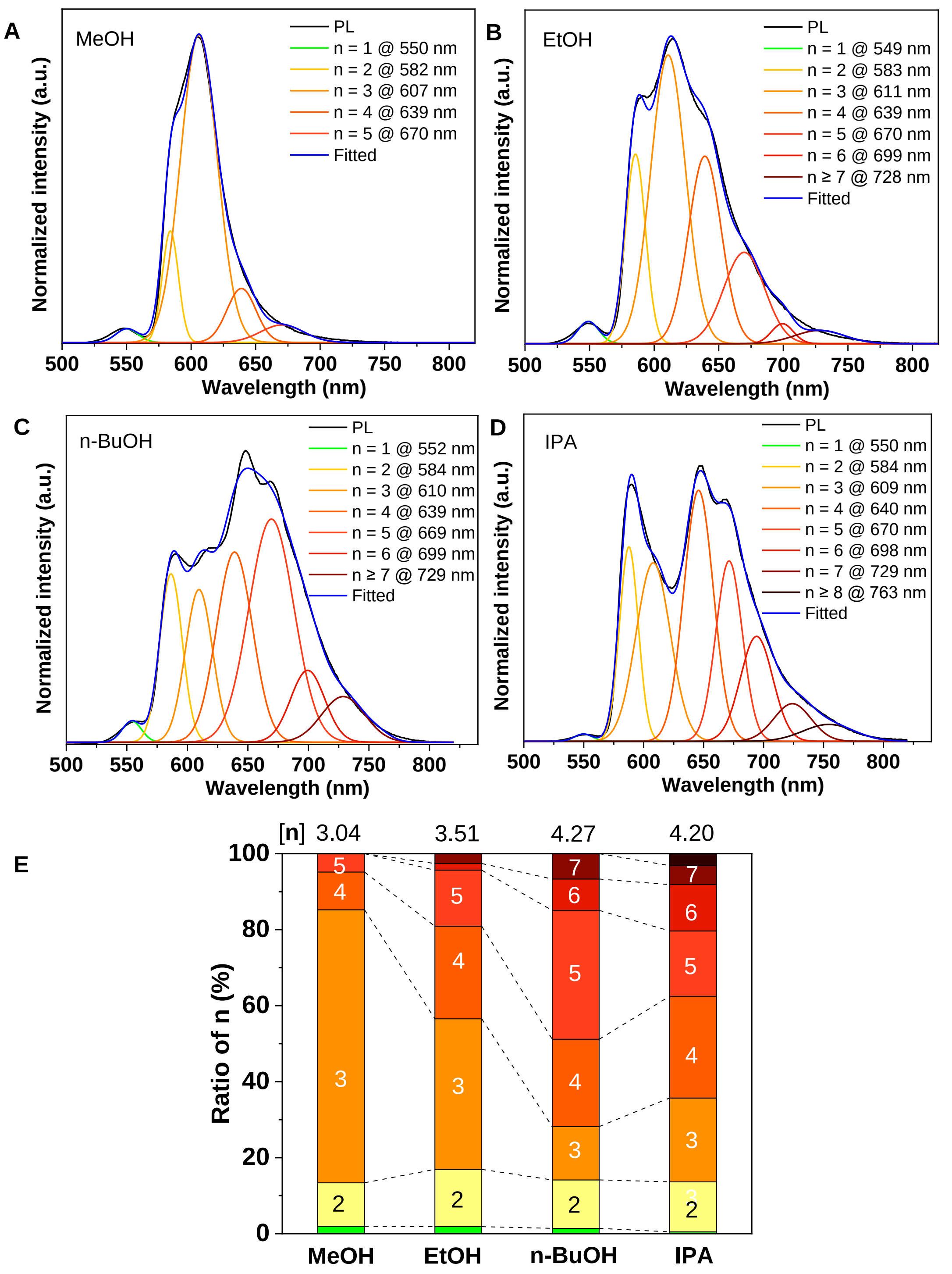

F

n	1	2	3	4	5	6	7	≥ 8	[n]
MeOH	1.9	11.4	71.9	9.9	4.8	0.0	0.0	0.0	3.04
EtOH	1.8	15.0	39.6	24.4	14.8	1.7	2.6	0.0	3.51
n-BuOH	1.4	12.8	14.0	23.0	33.9	8.3	6.7	0.0	4.27
IPA	0.5	13.1	22.1	26.7	17.2	12.2	5.0	3.1	4.20

Fig. S12. PL and multiple peaks fitting results of $MAPbI_3$ NPs crude solution diluted 100 times with toluene. Prepared by different alcohols. (**A**) MeOH. (**B**) EtOH, (**C**) n-BuOH, (**D**) IPA. (**E**) the 2D graph of PL intensity ratio (%) from multiple peaks fitting results. (**F**) The ratio (%) of different phases from the fitting results.

A

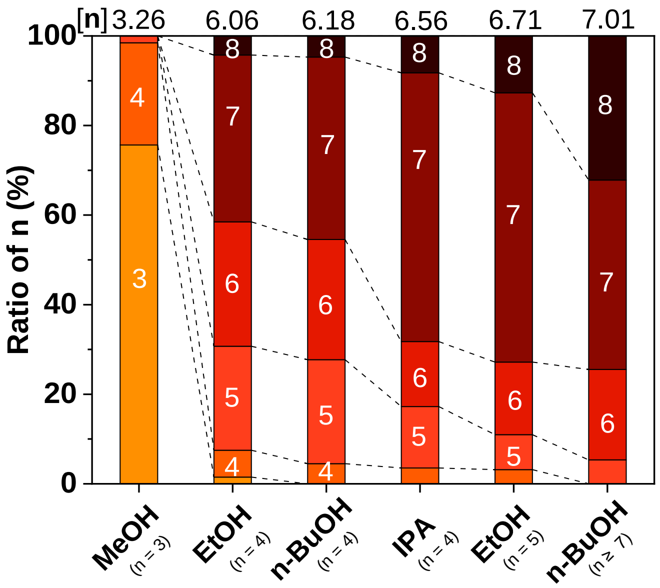

B

n	1	2	3	4	5	6	7	≥ 8	[n]
**MeOH(n = 3)**	0.0	0.0	75.6	22.8	1.5	0.0	0.0	0.0	3.26
**EtOH(n = 4)**	0.0	0.0	1.5	6.0	23.2	27.8	37.2	4.3	6.06
**n-BuOH(n = 4)**	0.0	0.0	0.0	4.5	23.2	26.8	40.7	4.7	6.18
**IPA(n = 4)**	0.0	0.0	0.0	3.5	13.7	14.5	60.0	8.2	6.56
**EtOH(n = 5)**	0.0	0.0	0.0	3.2	7.8	16.2	60.1	12.7	6.71
**n-BuOH(n ≥ 7)**	0.0	0.0	0.0	0.0	5.3	20.2	42.3	32.2	7.01

Fig. S13. PL and fitting results of $MAPbI_3$ NP solutions (2.0 mg·mL^{-1}) for LED preparation. (**A**) 2D graph of PL intensity ratio (%) from multiple peaks fitting results. (**B**) The ratio (%) of different phases from the fitting results. More fitting details in Fig. S2.

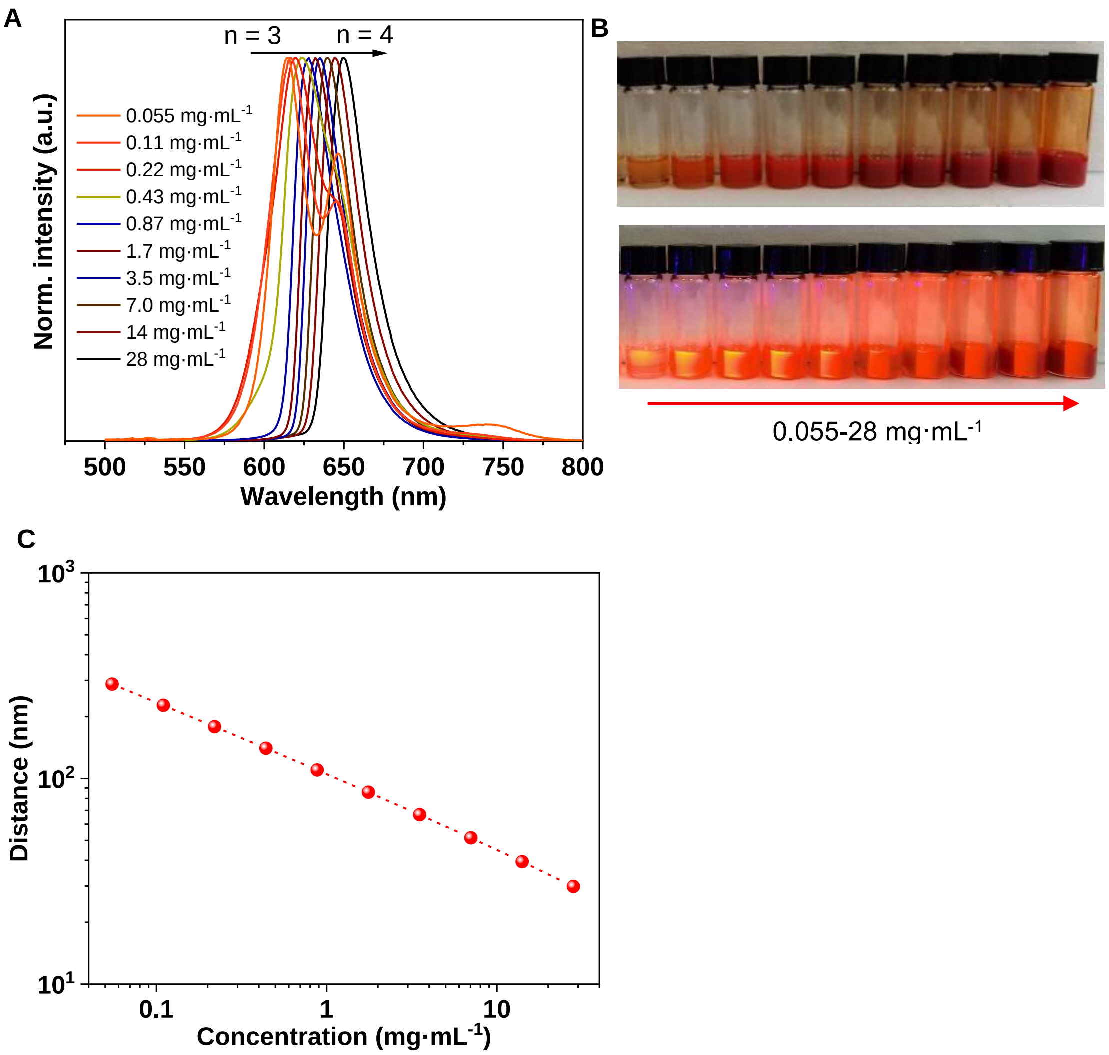

Fig. S14. 0.055-28 mg·mL^{-1} $MAPbI_3$ NP solutions synthesized by MeOH. (**A**) PL spectra. (**B**) Photos of solutions and solutions under 395 nm UV lamp. (**C**) Estimated distance between $MAPbI_3$ NPs in different concentration solutions.

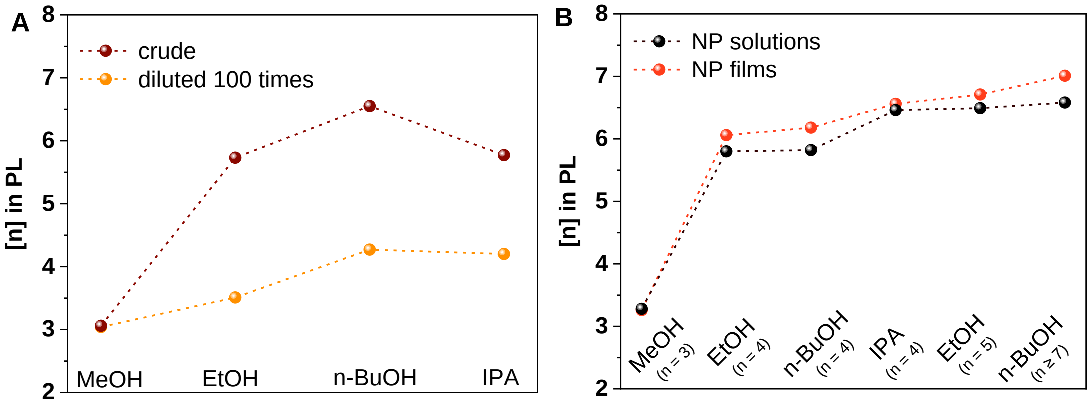

Fig. S15. [n] in PL of $MAPbI_3$ solutions and films. (**A**) crude and diluted crude NP solutions. (**B**) [n] in PL of NP solutions (2.0 mg/mL) and films for LEDs.

Surface composition analysis of $MAPbI_3$ NPs by different alcohols

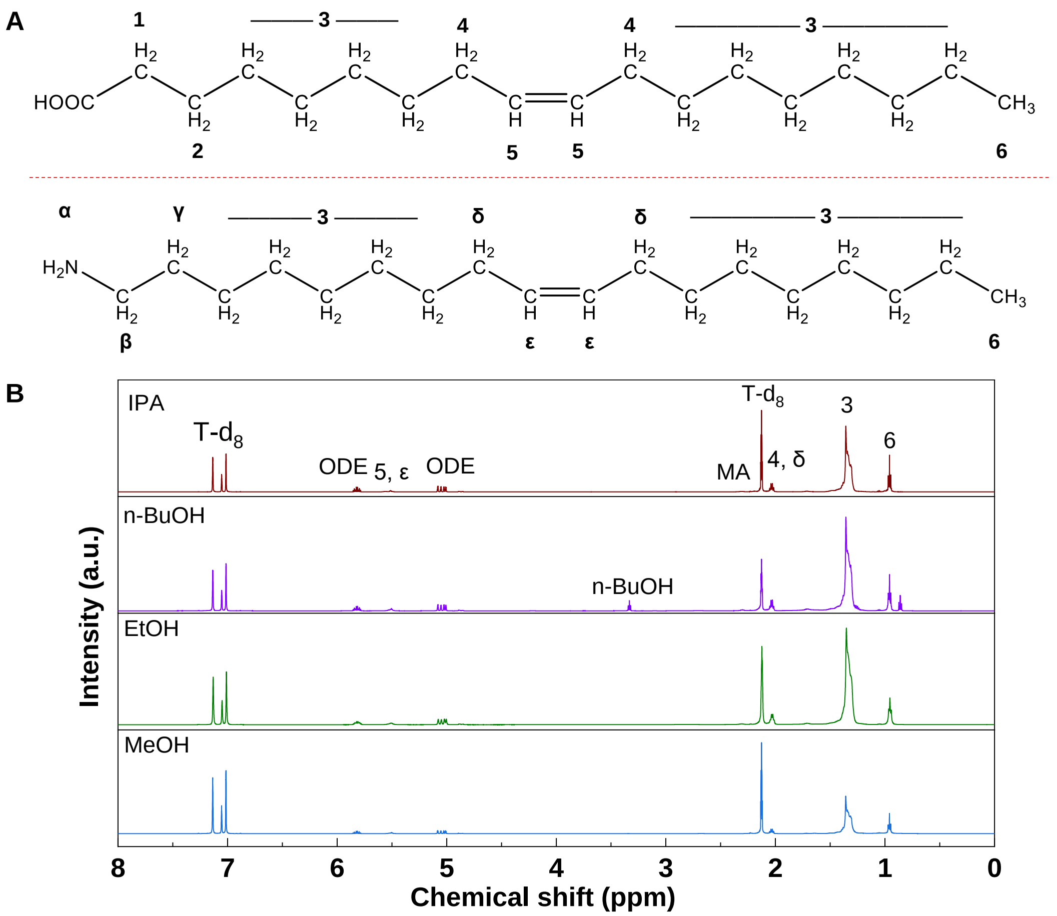

Fig. S16. ^{1}H-NMR of $MAPbI_3$ NP solutions. (**A**) Molecular structure of OA and OM. (**B**) ^{1}H-NMR spectra of $MAPbI_3$ NP solutions prepared by dispersing NPs into deuterated toluene (T-d_8, $C_6D_5CD_3$, toluene-d_8).

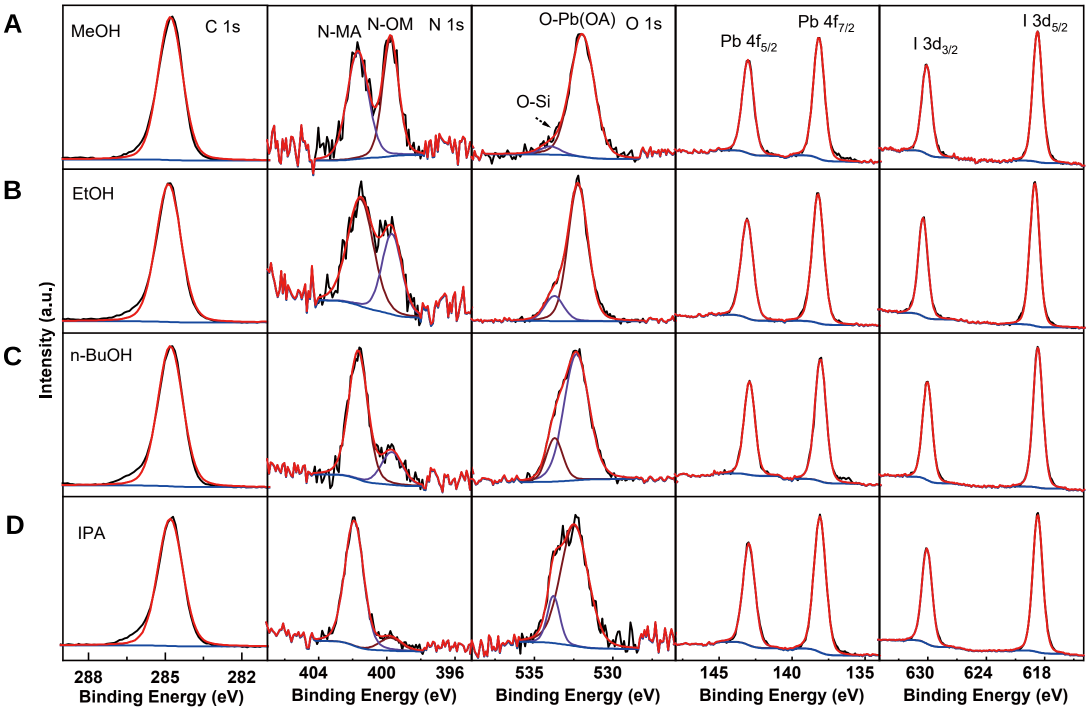

Fig. S17. XPS, C 1s, N 1s, O1s, Pb 4f, I 3d, of $MAPbI_3$ NPs. Prepared by (**A**) MeOH; (**B**) EtOH; (**C**) n-BuOH, (**D**) IPA. Samples prepared by dropping the final solutions (2.0 mg·mL^{-1}) on a silicon wafer. (N-MA)/(N-OM) ratio means N ratio from MA^+ (BE, 401.5 eV) to OM^+ (BE, 399.7 eV). BE, binding energy.

The conspicuously low I/Pb ratio (~2.0, lower than typical quasi-2D, (3n+1)/n) of the NP surface is ascribed to OA^- partially replace iodide and coordinate with the Pb atom as XPS O1s fitting results proved, this coordinated OA^- makes NPs surrounded with long-chain ligands and more isolated from each other.

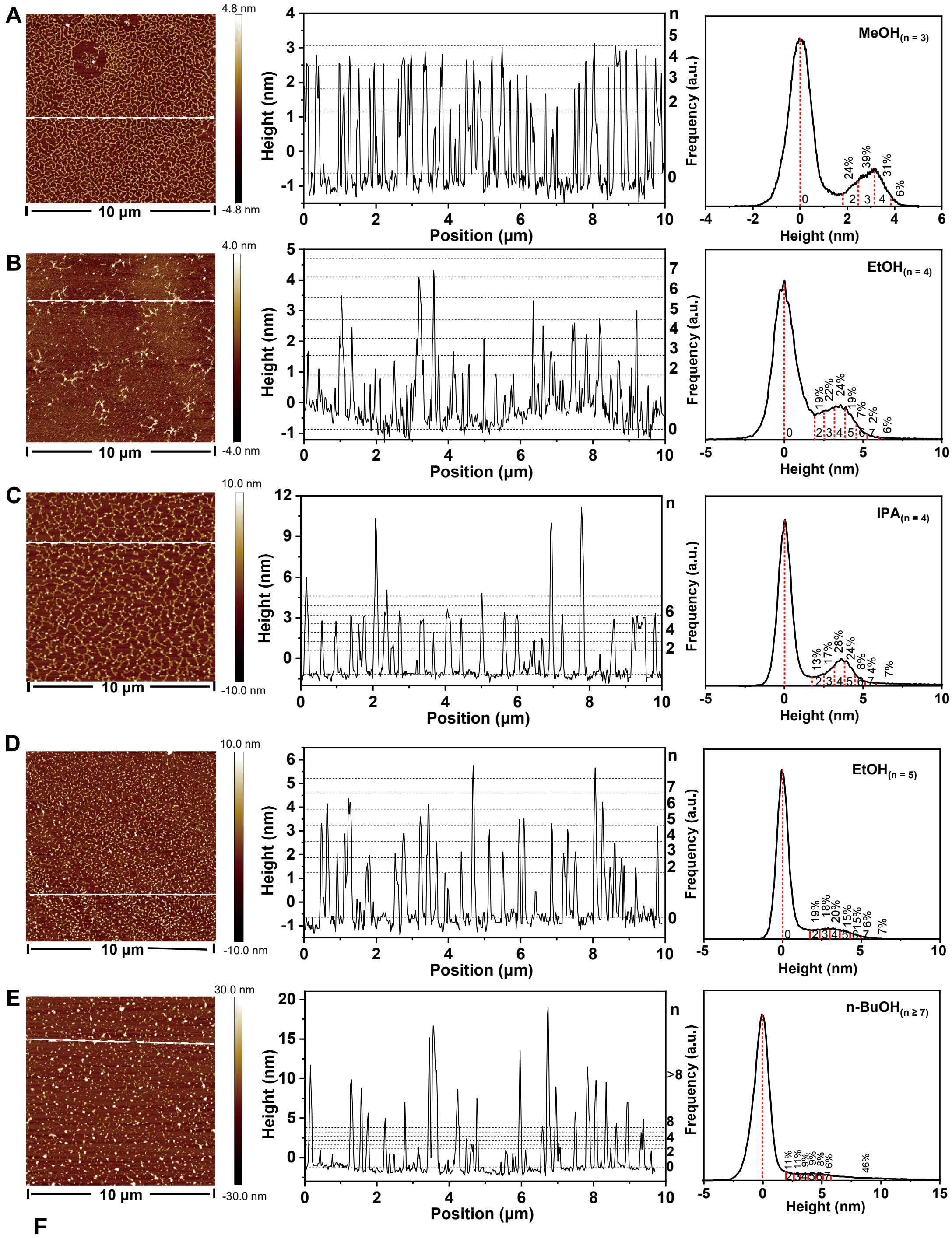

F

Ratio (%)	2	3	4	5	6	7	≥8	[n]
**MeOH**$_{(n = 3)}$	24	39	31	6				3.19
**EtOH**$_{(n = 4)}$	19	22	24	19	7	2	6	4.05
**n-BuOH**$_{(n = 4)}$	21	24	18	13	7	4	13	4.23
**IPA**$_{(n = 4)}$	13	17	28	24	8	4	7	4.38
**EtOH**$_{(n = 5)}$	19	18	20	15	15	6	7	4.33
**n-BuOH**$_{(n \geq 7)}$	11	11	9	9	8	6	46	5.91

Fig. S18. Thickness and real n-phase ratio distribution of NPs via AFM. AFM images with dash line, height along the dashed line and height statistics result of $MAPbI_3$ NPs prepared by (**A**) MeOH$_{(n = 3)}$; (**B**) EtOH$_{(n = 4)}$; (**C**) IPA$_{(n = 4)}$; (**D**) EtOH$_{(n = 5)}$; (**E**) n-BuOH$_{(n \geq 7)}$. (**F**) n-phase ratio (%). Diluted NP solutions (~0.5 mg·mL^{-1}) were spin-coated on silicon wafers to form discontinuous films for testing.

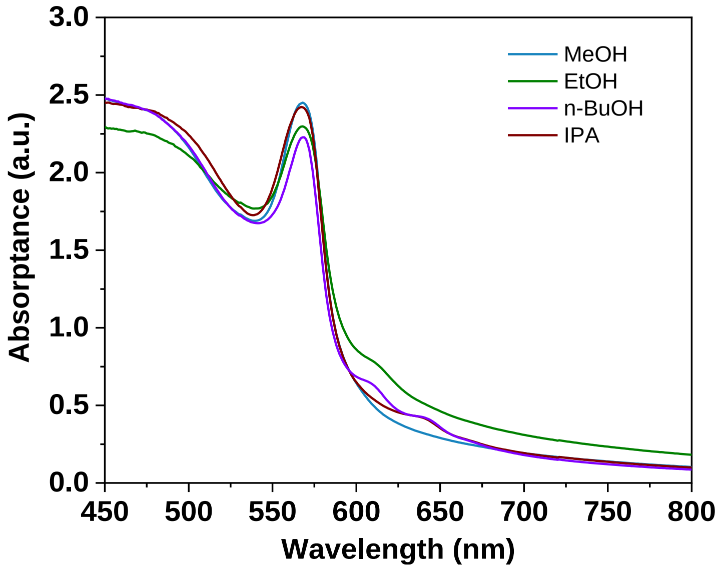

Fig. S19. UV-Vis of $MAPbI_3$ NPs crude solutions. Prepared by MeOH, EtOH, n-BuOH, IPA, diluted 100 times with toluene.

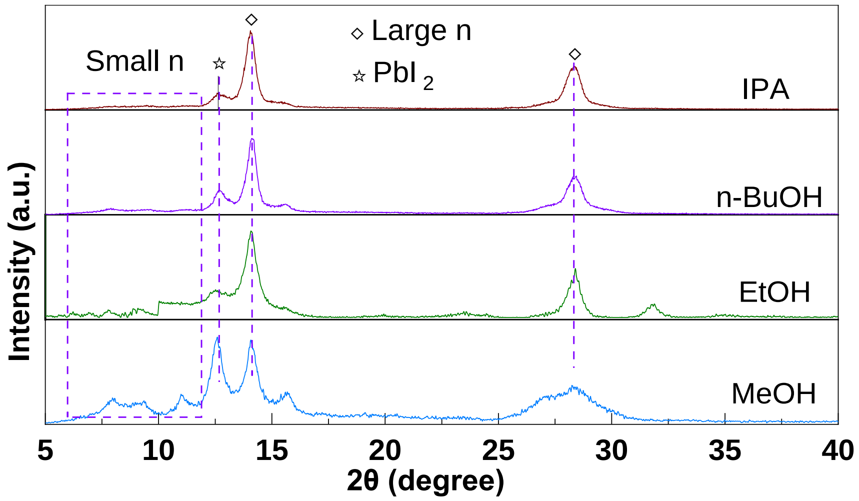

Fig. S20. XRD of $MAPbI_3$ NPs prepared by different alcohols.

A

n		3	4	5	6	≥ 7	≥ 8	[n]
**MeOH$_{(n=3)}$**	PL	78.6	19.1	2.3				3.23
	EL	87.9	12.1					3.12
**EtOH$_{(n=4)}$**	PL	2.3	13.8	21.2	28.5	25.9	6.8	5.75
	EL	7.7	52.9	28.2	10.1	1.0		4.43
**n-BuOH$_{(n=4)}$**	PL		11.0	28.4	32.4	24.4	3.8	5.82
	EL		65.4	27.1	5.4	2.1		4.44
**IPA$_{(n=4)}$**	PL		6.8	14.1	24.0	45.4	9.8	6.37
	EL		61.4	26.6	9.6	2.4		4.53
**EtOH$_{(n=5)}$**	PL		6.8	15.8	26.0	41.3	10.0	6.32
	EL		10.3	62.1	25.9	1.7		5.19
**n-BuOH$_{(n\geq7)}$**	PL				26.4	53.0	20.6	6.94
	EL				15.4	80.4	4.3	6.89

B

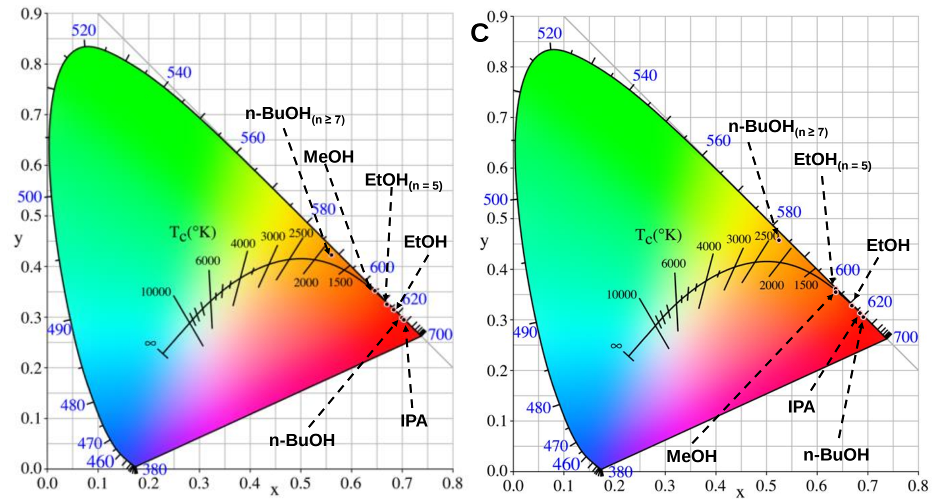

D

	PL of $MAPbI_3$ NP films		EL of $MAPbI_3$ NP LEDs	
**NPs**	**CIE (x)**	**CIE (y)**	**CIE (x)**	**CIE (y)**
**MeOH$_{(n=3)}$**	0.6461	0.3525	0.6376	0.3610
**EtOH$_{(n=4)}$**	0.6843	0.3142	0.6691	0.3282
**n-BuOH$_{(n=4)}$**	0.6995	0.2992	0.6925	0.3059
**IPA$_{(n=4)}$**	0.7045	0.2941	0.685	0.3129
**EtOH$_{(n=5)}$**	0.6713	0.3253	0.6375	0.3544
**n-BuOH$_{(n\geq7)}$**	0.5617	0.4232	0.5249	0.4567

Fig. S21. Comparison of PL of NP films and EL of LEDs. (**A**) Ratio of different n-phases from the fitting results of PL and EL. (**B**) Coordinates of PL of $MAPbI_3$ NP films and (**C**) EL of $MAPbI_3$ NP LEDs in CIE 1931 Chromaticity Coordinate. (**D**) Coordinate data of PL and EL.

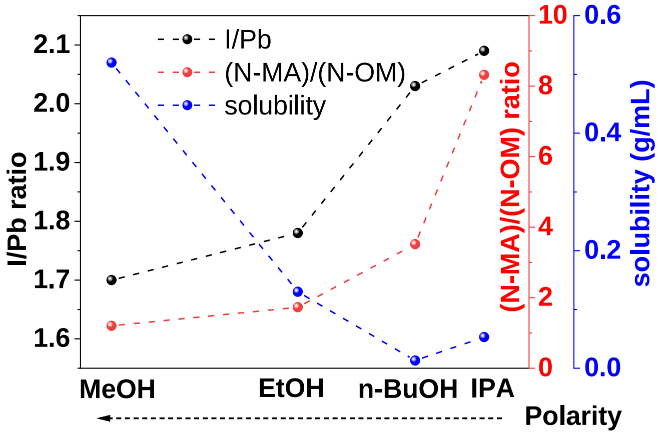

Fig. S22. Relevance of relative polarity of polar solvents, I/Pb ratio, (N-MA)/(N-OM) ratio of $MAPbI_3$ NPs. N-MA)/(N-OM) ratio means N from MA^+ to N from OM^+, tested by XPS. NPs were prepared by different alcohols and solubility (in 1 mL alcohol) of MAI in different alcohols.

Table S5. Relative polarity of alcohols, I/Pb ratio, the ratio of N from MA^+ (binding energy, BE, 401.5 eV) and OM^+ (BE, 399.7 eV) of $MAPbI_3$ NP solutions. Synthesized by MeOH, EtOH, n-BuOH, IPA and solubility (in 1 mL alcohols) of MAI in different alcohols.

Alcohols	Relative polarity	Solubility (g/mL)	I/Pb	(N-MA)/(N-OM)
**MeOH**	0.762	0.52	1.70	1.20
**EtOH**	0.654	0.13	1.78	1.73
**n-BuOH**	0.586	0.013	2.03	3.52
**IPA**	0.546	0.053	2.09	8.32

UV-Vis of $MAPbI_3$ NP solutions

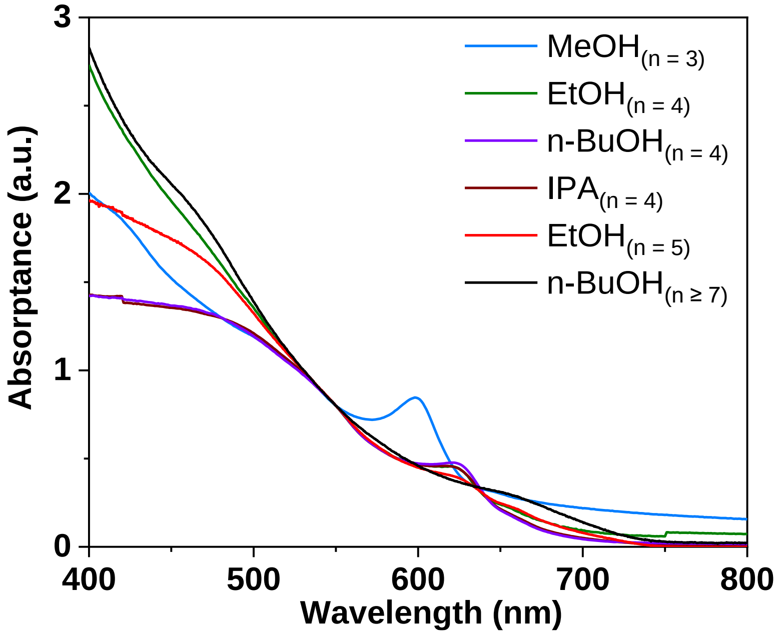

Fig. S23. UV-Vis of $MAPbI_3$ NP solutions (0.2 mg/mL). Prepared by $MeOH_{(n = 3)}$, $EtOH_{(n = 4)}$, n-$BuOH_{(n = 4)}$, $IPA_{(n = 4)}$, $EtOH_{(n = 5)}$, n-$BuOH_{(n \geq 7)}$.

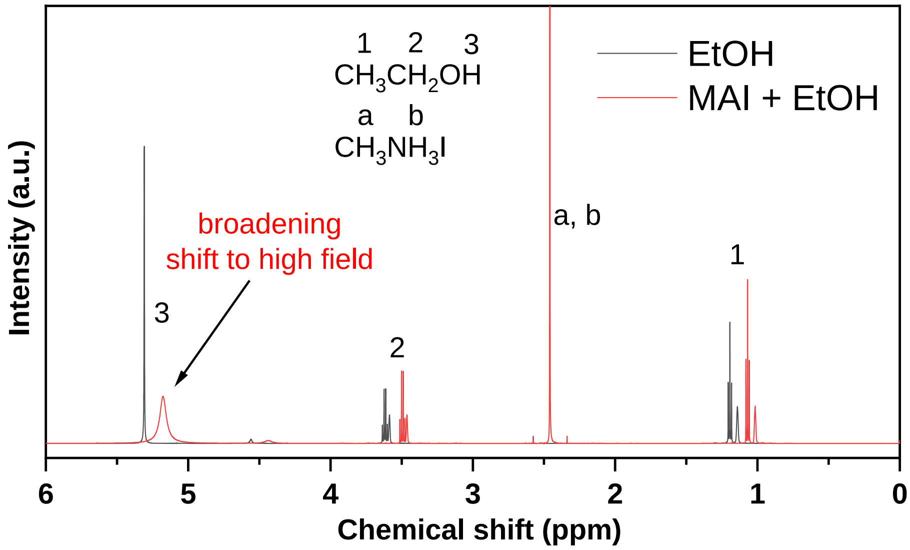

Fig. S24. ^{1}H-NMR of MAI solution in EtOH. The EtOH is from the un-purified deuterated EtOH (CD_3CD_2OD), and MAI + EtOH was prepared by dissolving MAI into CD_3CD_2OD.

PLQE of NP solutions/films

Table S6. PLQE of $MAPbI_3$ NP solutions and films. Prepared by $MeOH_{(n = 3)}$, $EtOH_{(n = 4)}$, $n\text{-}BuOH_{(n = 4)}$, $EtOH_{(n = 5)}$, $IPA_{(n = 4)}$, $n\text{-}BuOH_{(n \geq 7)}$.

Sample	$MeOH_{(n = 3)}$	$EtOH_{(n = 4)}$	$n\text{-}BuOH_{(n = 4)}$	$EtOH_{(n = 5)}$	$IPA_{(n = 4)}$	$n\text{-}BuOH_{(n \geq 7)}$
Solutions	38.0	71.1	77.8	81.3	76.5	38.4
Films	29.4	61.3	68.8	70.7	68.4	19.8

MAPbI$_3$ NP LEDs with high EQE

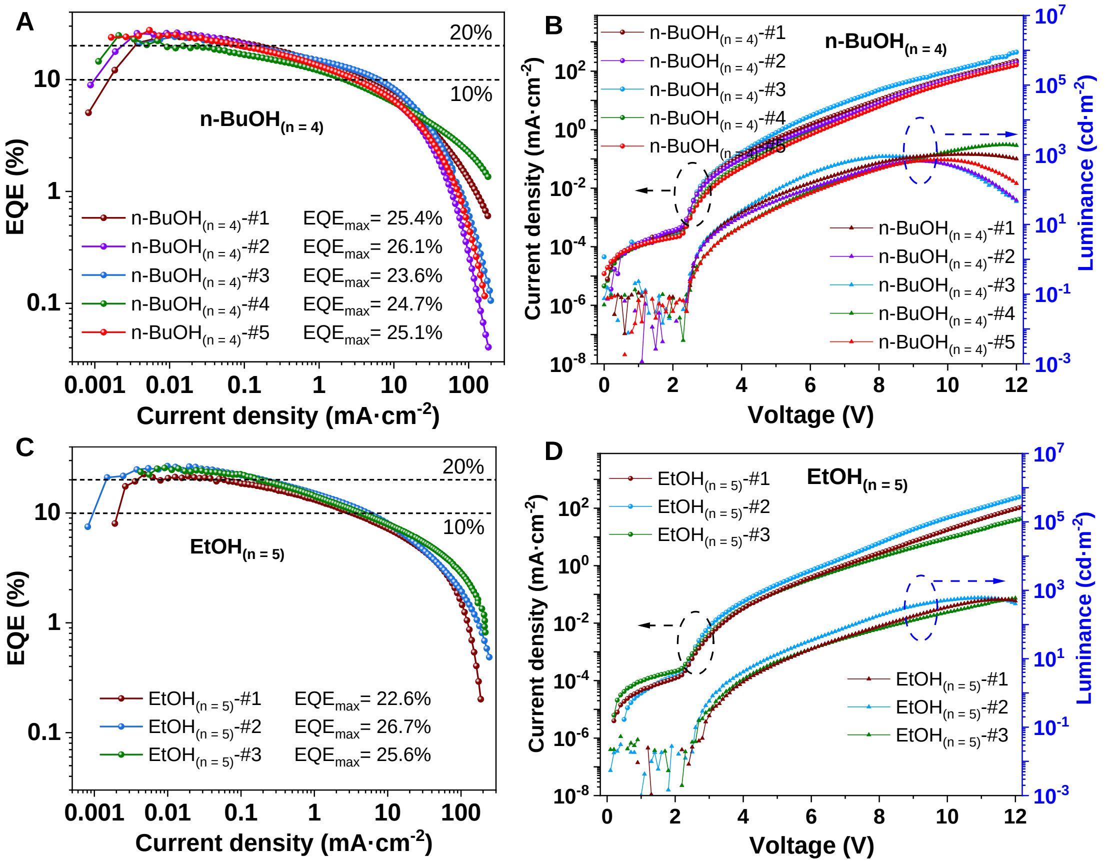

E

NPs	Max. EQE (%) at J (mA·cm^{-2})	Luminance (cd·m^{-2}) and J (mA·cm^{-2}) at EQE = 20%	Max. photon flux photo (sr^{-1}·s^{-1})	Max. luminance (cd·m^{-2}) at J (mA·cm^{-2})
**n-BuOH$_{(n=4)}$-#1**	25.4 (1.9 × 10^{-2})	24.5 (1.6 × 10^{-1})	1.18 × 10^{14}	1.03 × 10^{3} (8.6 × 10^{1})
**n-BuOH$_{(n=4)}$-#2**	26.1 (1.3 × 10^{-2})	18.7 (1.2 × 10^{-1})	7.59 × 10^{13}	6.63 × 10^{2} (2.5 × 10^{1})
**n-BuOH$_{(n=4)}$-#3**	23.6 (1.7 × 10^{-2})	13.2 (8.2 × 10^{-2})	1.03 × 10^{14}	9.00 × 10^{2} (2.9 × 10^{1})
**n-BuOH$_{(n=4)}$-#4**	24.7 (2.1 × 10^{-3})	3.4 (2.3 × 10^{-2})	2.24 × 10^{14}	1.96 × 10^{3} (1.7 × 10^{2})
**n-BuOH$_{(n=4)}$-#5**	25.1 (1.2 × 10^{-2})	14.3 (9.0 × 10^{-2})	8.27 × 10^{13}	7.21 × 10^{2} (3.2 × 10^{1})
**EtOH$_{(n=5)}$-#1**	22.6 (4.8 × 10^{-3})	3.6 (5.9 × 10^{-2})	1.60 × 10^{14}	5.42 × 10^{2} (7.0 × 10^{1})
**EtOH$_{(n=5)}$-#2**	26.7 (1.0 × 10^{-2})	12.1 (1.9 × 10^{-1})	1.79 × 10^{14}	6.06 × 10^{2} (1.2 × 10^{2})
**EtOH$_{(n=5)}$-#3**	25.6 (9.1 × 10^{-3})	10.7 (1.7 × 10^{-1})	2.68 × 10^{14}	9.09 × 10^{2} (1.3 × 10^{2})

Fig. S25. MAPbI$_3$ NP LEDs with high EQE prepared by n-BuOH$_{(n=4)}$ and EtOH$_{(n=5)}$. (**A**) EQE-current density curves and (**B**) current density-voltage (J-V) and luminance-voltage curves of LEDs prepared by n-BuOH. (**C**) EQE-current density curves and (**D**) current density-voltage (J-V) and luminance-voltage curves of LEDs of LEDs prepared by EtOH$_{(n=5)}$. (**E**) Performance parameters. J means current density (mA·cm^{-2}).

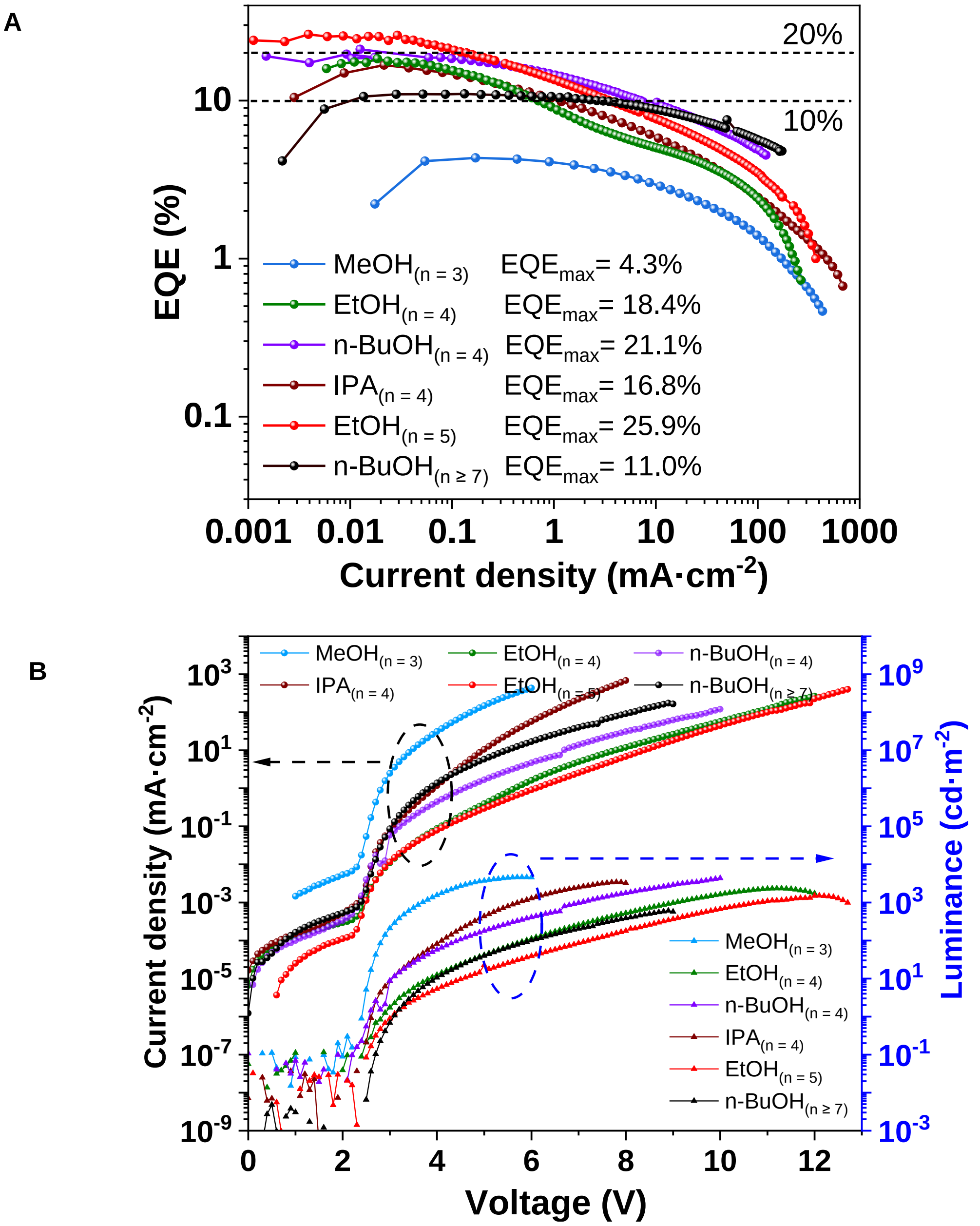

C

NPs	Max. EQE (%) at J (mA·cm^{-2})	Max. photon flux photo·(sr^{-1}·s^{-1})	Max. luminance (cd·m^{-2}) at J (mA·cm^{-2})	$L_{(h. l.)}/L_{(h. EQE)}$ (times)
**MeOH$_{(n = 3)}$**	4.3 (4.4 × 10^{-1})	1.82 × 10^{14}	4.62 × 10^{3} (3.6 × 10^{2})	2.05
**EtOH$_{(n = 4)}$**	18.4 (1.9 × 10^{-2})	2.37 × 10^{14}	2.34 × 10^{3} (1.5 × 10^{2})	1.05
**n-BuOH$_{(n = 4)}$**	21.1 (1.3 × 10^{-2})	4.87 × 10^{14}	4.33 × 10^{3} (1.5 × 10^{2})	5.29
**IPA$_{(n = 4)}$**	16.8 (2.2 × 10^{-2})	4.34 × 10^{14}	3.42 × 10^{3} (1.2 × 10^{2})	5.87
**EtOH$_{(n = 5)}$**	25.7 (2.9 × 10^{-2})	4.37 × 10^{14}	1.50 × 10^{3} (2.5 × 10^{2})	3.36
**n-BuOH$_{(n \geq 7)}$**	11.0 (1.3 × 10^{-1})	7.41 × 10^{14}	6.03 × 10^{2} (1.7 × 10^{2})	8.46

Fig. S26. $MAPbI_3$ NP LEDs with high luminance. Obtained by increasing the thickness of the NPs layer and reducing the thickness of the charge transfer layer. (**A**) EQE-current density curves of LEDs. (**B**) Current density-voltage (J-V) and luminance-voltage curves of LEDs. (**C**) Parameters and comparison with the LEDs with the highest EQE. $L_{(h. l.)}/L_{(h. EQE)}$ means the luminance of devices with high luminance to the luminance of devices with the highest EQE.

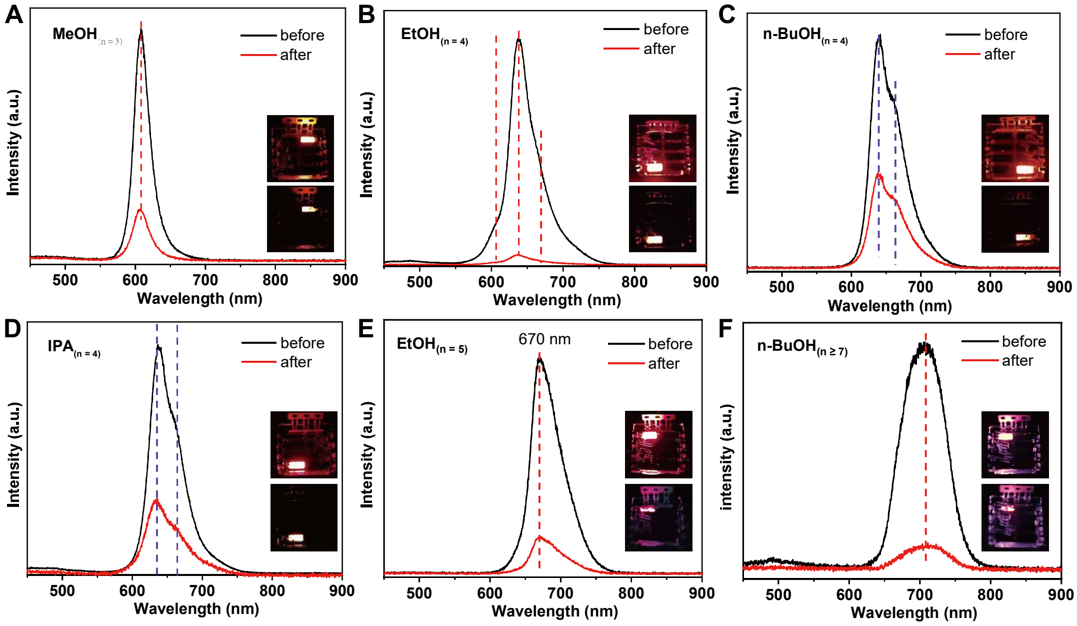

Fig. S27. Spectra stability test of MAPbI3 NP LEDs. Prepared by (**A**) MeOH**(n = 3)**; (**B**) EtOH**(n = 4)**; (**C**) BuOH**(n = 4)**; (**D**) IPA**(n = 4)**; (**E**) EtOH(n = 5); (**F**) n-BuOH(n ≥ 7). EL spectra before and after the stability test and pictures of LEDs before (upside) and after the stability test (downside).

UPS of NP films

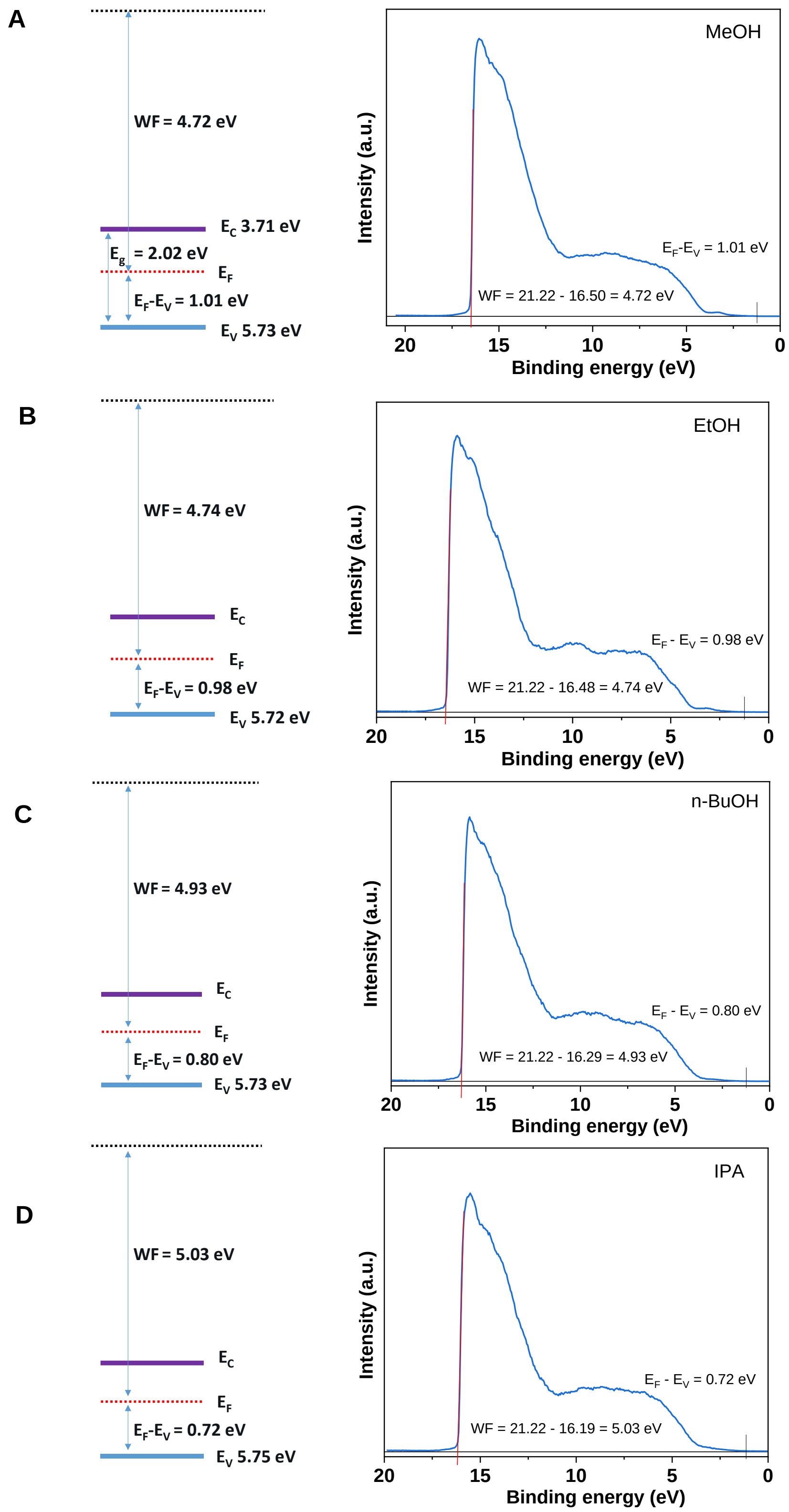

Fig. S28. UPS of $MAPbI_3$ NPs. Prepared by (**A**) MeOH. (**B**) EtOH. (**C**) n-BuOH. (**D**) IPA. Samples were prepared by dropping $MAPbI_3$ NP solutions (2.0 $mg \cdot mL^{-1}$, after washing with ethyl acetate) on a silicon wafer. Work function (WF), Fermi energy (E_F), band-gap energy (E_g), the energy of conduction band minima (E_c), energy of valence band maxima (E_v).

TA of NP solutions/films

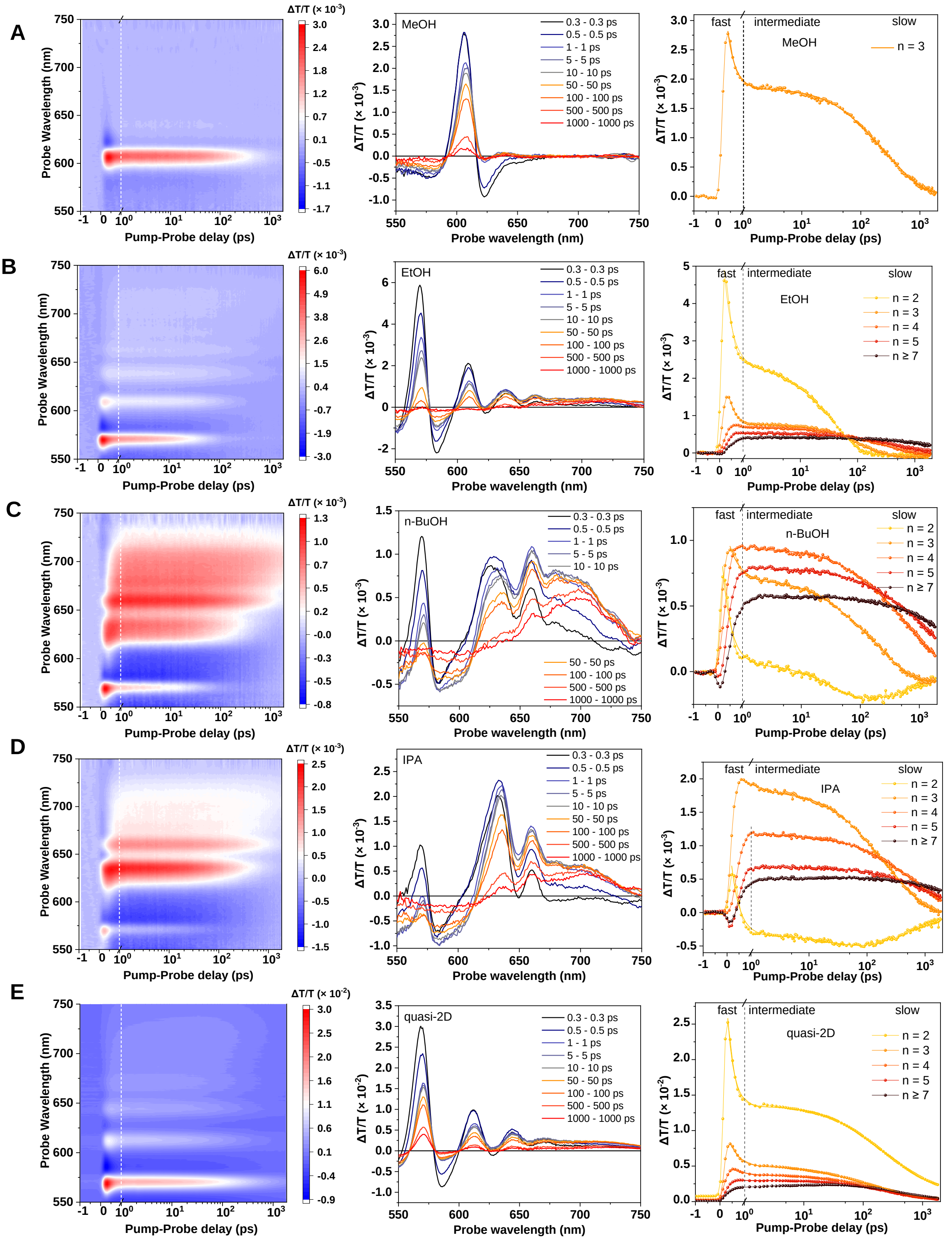

F

	n	fast τ_1 (ps)	intermediate τ_2 (ps)	slow τ_3 (ps)
**MeOH**	3	0.32	92	450
	2	0.36	9.3	42
	3	0.28	124	97
**EtOH**	4	/	54	437
	5	/	60	579
	≥7	/	64	2064
	2	0.36	28	/
	3	0.44	38	278
**n-BuOH**	4	/	87	750
	5	/	126	1209
	≥7	/	179	3338
	2	0.35	20	/
	3	0.88	64	407
**IPA**	4	3.6	119	934
	5	/	128	1158
	≥7	/	204	2596
	2	0.27	77	500
	3	0.38	36	296
**Quasi-2D**	4	0.47	66	383
	5	/	156	776
	7	/	274	689

Fig. S29. Transient absorption spectra of $MAPbI_3$ NP films. The 2D spectrograph of time-resolved absorbance, transient absorption spectra and decay kinetics of $MAPbI_3$ NP films on glass prepared by: (**A**) MeOH**;** (**B**) EtOH; (**C**) n-BuOH; (**D**) IPA; (**E**) quasi-2D, correspondingly. (**F**) Fitting results of transient absorption decay kinetics. The excitation fluence for the transient absorption measurement was 5.09 $\mu J \cdot cm^{-2}$. Samples were prepared by spin-coating NP solutions (2.0 $mg \cdot mL^{-1}$) on glass substrates, and encapsulated with cover glass.

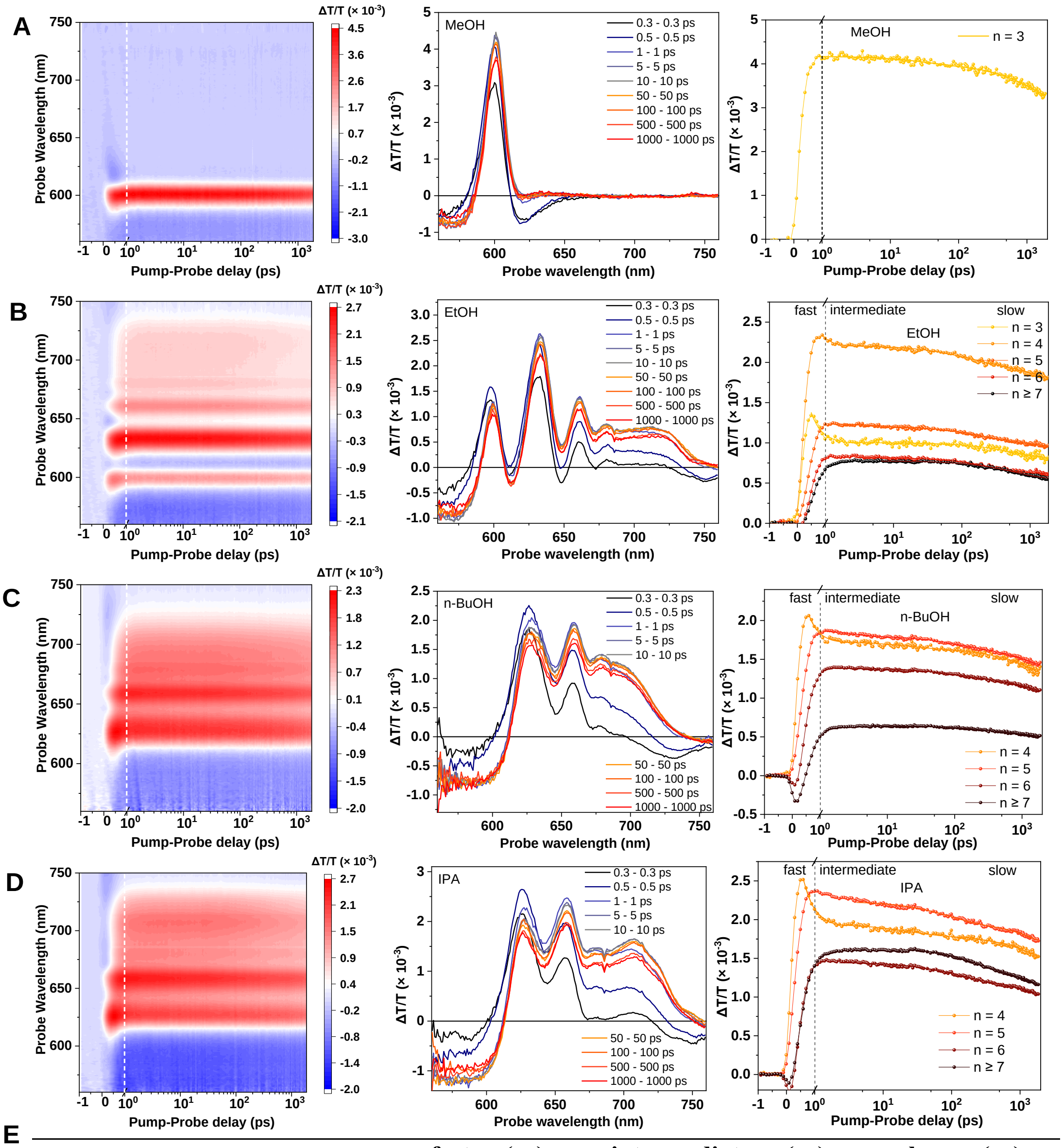

E

	n	fast τ_1 (ps)	intermediate τ_2 (ps)	slow τ_3 (ps)
**MeOH**	3	/	29	1424
**EtOH**	3	0.38	/	608
	4	0.34	90	965
	5	4.2	179	2515
	6	/	280	/
	≥7	/	607	/
**n-BuOH**	4	0.34	20	1521
	5	2.3	97	1892
	6	/	88	1778
	≥7	/	226	1850
**IPA**	4	0.36	23	1999
	5		85	2934
	6	/	104	2562
	≥7	/	189	2390

Fig. S30. Transient absorption spectra of $MAPbI_3$ NP solutions (2.0 mg·mL^{-1}). The 2D spectrograph of time-resolved absorbance, transient absorption spectra and decay kinetics of $MAPbI_3$ NP solutions by: (**A**) MeOH; (**B**) EtOH; (**C**) n-BuOH; (**D**) IPA, correspondingly. (**E**) Fitting results of transient absorption decay kinetics. The excitation fluence used was 5.09 μJ·cm^{-2}.

Conductivity test of 3D, quasi-2D and NP films

The conductivity of films, tested by the 4-probe method:

$$\sigma = Id/(HLV)$$

I is the current (A), d is the gap (135 μm) between electrodes, L is the length of electrodes (2000 μm), H is the thickness of perovskite films and tested by the profiler, V is the voltage between the 2nd and 3rd electrodes.

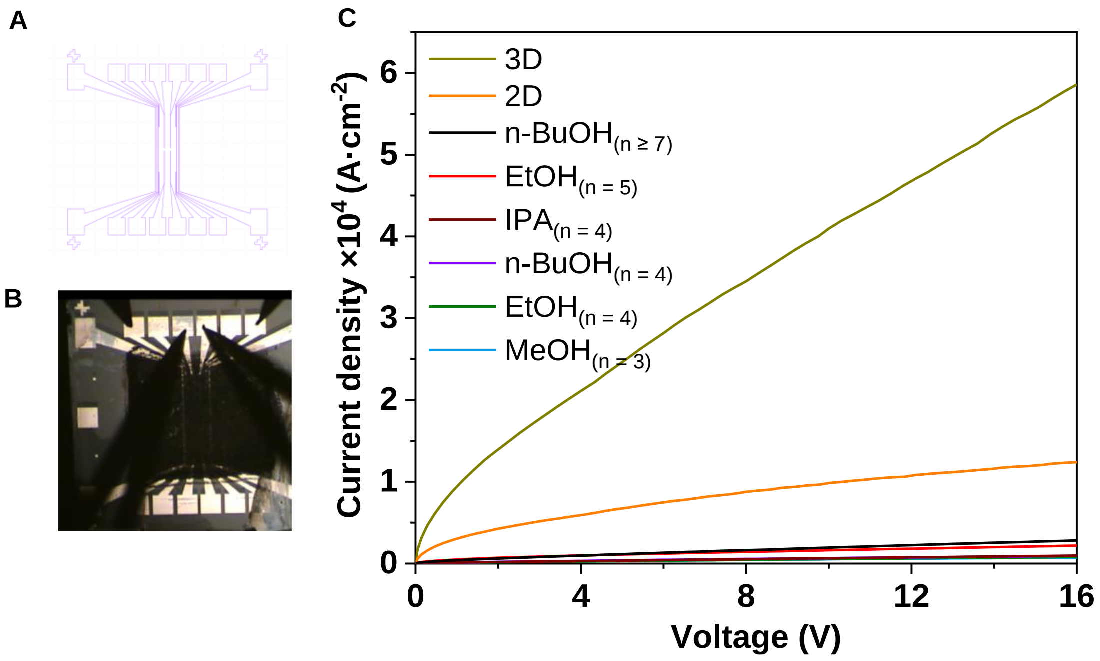

Fig. S31. Conductivity of films tested by 4-probe method. (**A**) The design drawing of electrodes (Cr/Au) for conductivity. (**B**) The optical image of the device for a conductivity test. (**C**) Current density ($A·cm^{-2}$) to voltage (V) curves.

Table S7. Correlation of ligand/Pb ratio (L/Pb), average real n phase ([n] via AFM), conductivity and average n phase in EL ([n] in EL). L/Pb was obtained from XPS data.

Samples	L/Pb	[n] via AFM	Conductivity ($S·cm^{-1}$)	[n] in EL
**$MeOH_{(n = 3)}$**	4.96	3.19	5.80×10^{-9}	3.12
**$EtOH_{(n = 4)}$**	2.99	4.05	6.40×10^{-9}	4.43
**$n\text{-}BuOH_{(n = 4)}$**	2.34	4.23	7.45×10^{-9}	4.44
**$IPA_{(n = 4)}$**	2.08	4.38	7.85×10^{-9}	4.53
**$EtOH_{(n = 5)}$**	2.01	4.33	1.63×10^{-8}	5.19
**$n\text{-}BuOH_{(n \geq 7)}$**	1.55	5.91	2.24×10^{-8}	6.89
**2D $MA_2PEA_2Pb_3I_{10}$**	0.67	3.00	9.41×10^{-8}	6-7
**3D $MAPbI_3$**	0	/	4.57×10^{-7}	3D

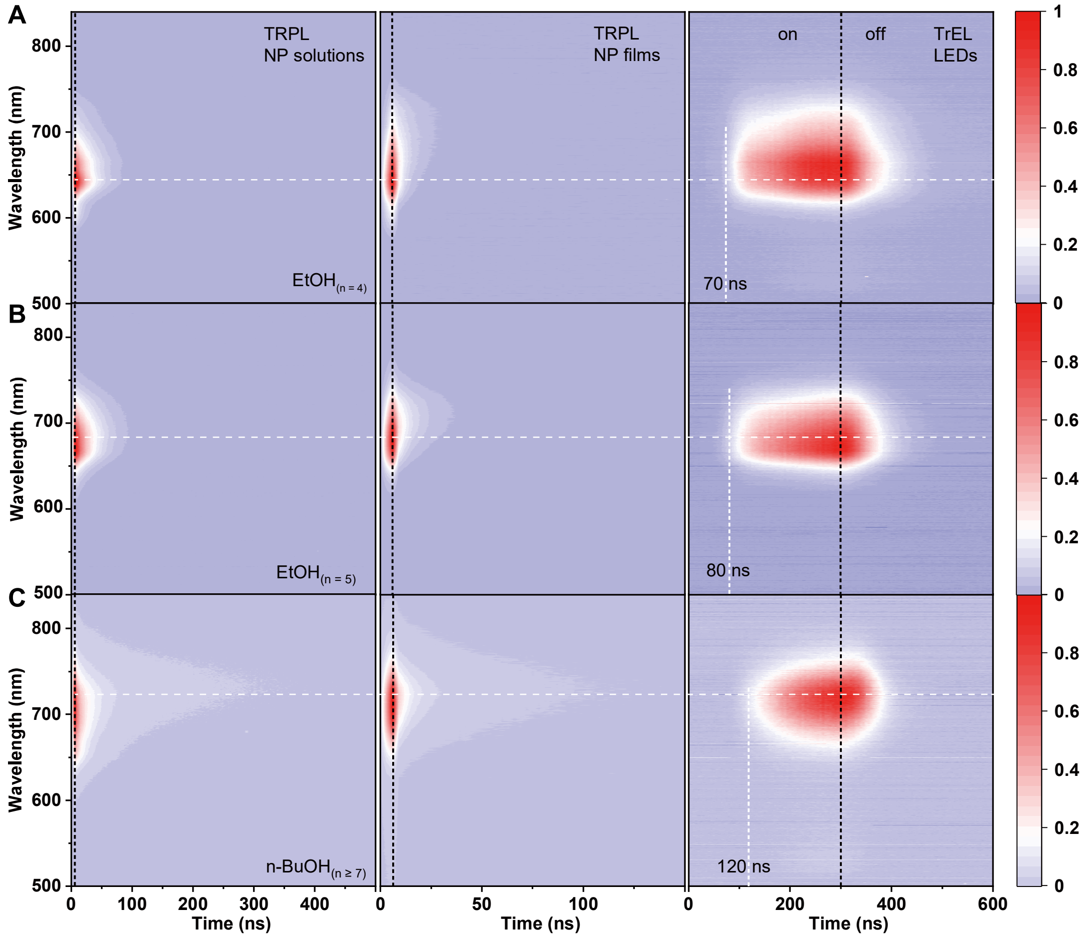

Fig. S32. TRPL of NP solutions & films and TrEL and of NP LEDs. Prepared by (**A**) EtOH$_{(n=4)}$; (**B**) EtOH$_{(n=5)}$ and (**C**) n-BuOH$_{(\mathbf{n} \geq 7)}$. In TREL, 0-300 ns, turn-on; 300-600 ns, turn-off.

EL of LEDs before/after ligand exchange

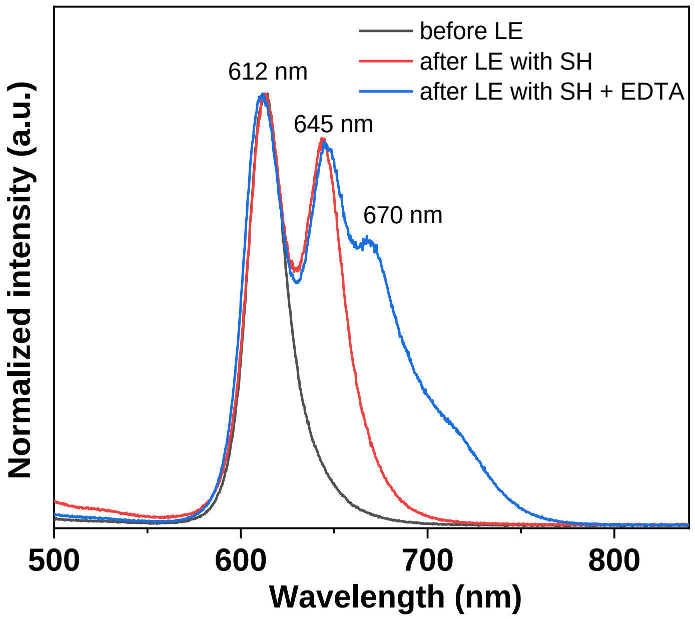

Fig. S33. EL spectra of $MAPbI_3$ LEDs synthesized by MeOH before and after ligand exchange. (SH: L-Cysteine, EDTA: Ethylenediaminetetraacetic acid).

Removal of ligands on the surface by the ligand exchange (LE) method can promote the charge transfer between different n-value NPs. A similar LE method inspired by a previous report by using the multidentate ligands method was attempted. Usage of L-Cysteine with -SH group alone and synergetic usage of L-Cysteine and Ethylenediaminetetraacetic acid (EDTA, multidentate -COOH) induced 2, even 3 EL peaks which attributed to the n = 3, 4, 5 NPs after ligand exchange.

Legends for Movie S1 and Data S1

Movie S1: The synthesis process of $MAPbI_3$ NPs using FEPS method.

Data S1: Original performance data of efficient $MAPbI_3$ NP LEDs.

REFERENCES AND NOTES

1. A. I. Ekimov, A. L. Efros, A. A. Onushchenko, Quantum size effect in semiconductor microcrystals. *Solid State Commun.* **56**, 921–924 (1985).

2. L. E. Brus, Electron–electron and electron-hole interactions in small semiconductor crystallites: The size dependence of the lowest excited electronic state. *J. Chem. Phys.* **80**, 4403–4409 (1984).

3. C. Murray, D. J. Norris, M. G. Bawendi, Synthesis and characterization of nearly monodisperse CdE (E= sulfur, selenium, tellurium) semiconductor nanocrystallites. *J. Am. Chem. Soc.* **115**, 8706–8715 (1993).

4. Z.-K. Tan, R. S. Moghaddam, M. L. Lai, P. Docampo, R. Higler, F. Deschler, M. Price, A. Sadhanala, L. M. Pazos, D. Credgington, Bright light-emitting diodes based on organometal halide perovskite. *Nat. Nanotechnol.* **9**, 687–692 (2014).

5. Y.-H. Kim, S. Kim, A. Kakekhani, J. Park, J. Park, Y.-H. Lee, H. Xu, S. Nagane, R. B. Wexler, D.-H. Kim, S. H. Jo, L. Martínez-Sarti, P. Tan, A. Sadhanala, G.-S. Park, Y.-W. Kim, B. Hu, H. J. Bolink, S. Yoo, R. H. Friend, A. M. Rappe, T.-W. Lee, Comprehensive defect suppression in perovskite nanocrystals for high-efficiency light-emitting diodes. *Nat. Photonics* **15**, 148–155 (2021).

6. J. Xing, F. Yan, Y. Zhao, S. Chen, H. Yu, Q. Zhang, R. Zeng, H. V. Demir, X. Sun, A. Huan, High-efficiency light-emitting diodes of organometal halide perovskite amorphous nanoparticles. *ACS Nano* **10**, 6623–6630 (2016).

7. Y. Hassan, J. H. Park, M. L. Crawford, A. Sadhanala, J. Lee, J. C. Sadighian, E. Mosconi, R. Shivanna, E. Radicchi, M. Jeong, Ligand-engineered bandgap stability in mixed-halide perovskite LEDs. *Nature* **591**, 72–77 (2021).

8. Y. Jiang, C. Sun, J. Xu, S. Li, M. Cui, X. Fu, Y. Liu, Y. Liu, H. Wan, K. Wei, Synthesis-on-substrate of quantum dot solids. *Nature* **612**, 679–684 (2022).

9. X. Zhang, Q. Huang, W. Yin, W. Zheng, Challenges in developing perovskite nanocrystals for commercial applications. *ChemPlusChem* **89**, e202300693 (2024).

10. J. Zhang, T. Zhang, Z. Ma, F. Yuan, X. Zhou, H. Wang, Z. Liu, J. Qing, H. Chen, X. Li, A multifunctional "halide-equivalent" anion enabling efficient $CsPb(Br/I)_3$ nanocrystals pure-red light-emitting diodes with external quantum efficiency exceeding 23%. *Adv. Mater.* ***35***, 2209002 (2022).

11. F. Zhao, H.-W. Duan, S.-N. Li, J.-L. Pan, W.-S. Shen, S.-M. Li, Q. Zhang, Y.-K. Wang, L.-S. Liao, Iodotrimethylsilane as a reactive ligand for surface etching and passivation of perovskite nanocrystals toward efficient pure-red to deep-red LEDs. *Angew. Chem. Int. Edit.* **62**, e202311089 (2023).

12. J. Zhang, W. Shen, S. Chen, Z. Zhang, B. Cai, Y. Qiu, Y. Liu, J. Jiang, Y. He, M. Nan, Multidentate ligand-passivated $CsPbI_3$ perovskite nanocrystals for stable and efficient red-light-emitting diodes. *J. Phys. Chem. Lett.* **14**, 6639–6646 (2023).

13. M. Xie, J. Guo, X. Zhang, C. Bi, L. Zhang, Z. Chu, W. Zheng, J. You, J. Tian, High-efficiency pure-red perovskite quantum-dot light-emitting diodes. *Nano Lett.* **22**, 8266–8273 (2022).

14. J. S. Kim, J.-M. Heo, G.-S. Park, S.-J. Woo, C. Cho, H. J. Yun, D.-H. Kim, J. Park, S.-C. Lee, S.-H. Park, Ultra-bright, efficient and stable perovskite light-emitting diodes. *Nature* **611**, 688–694 (2022).

15. Y.-H. Kim, J. Park, S. Kim, J. S. Kim, H. Xu, S.-H. Jeong, B. Hu, T.-W. Lee, Exploiting the full advantages of colloidal perovskite nanocrystals for large-area efficient light-emitting diodes. *Nat. Nanotechnol.* **17**, 590–597 (2022).

16. T. Chiba, Y. Hayashi, H. Ebe, K. Hoshi, J. Sato, S. Sato, Y.-J. Pu, S. Ohisa, J. Kido, Anion-exchange red perovskite quantum dots with ammonium iodine salts for highly efficient light-emitting devices. *Nat. Photonics* **12**, 681–687 (2018).

17. Y. Han, X. Chang, X. Cheng, Y. Lin, B. B. Cui, Recent progress of organic–inorganic hybrid perovskite quantum dots: Preparation, optical regulation, and application in light-emitting diodes. *Laser Photonics Rev.* **17**, 2300383 (2023).

18. J. Zhang, B. Cai, X. Zhou, F. Yuan, C. Yin, H. Wang, H. Chen, X. Ji, X. Liang, C. Shen, Ligand-induced cation-Π interactions enable high-efficiency, bright and spectrally stable rec. 2020 pure-red perovskite light-emitting diodes. *Adv. Mater.* **35**, 2303938 (2023).

19. Q. Zhang, D. Zhang, B. Cao, S. Poddar, X. Mo, Z. Fan, Improving the operational lifetime of metal-halide perovskite light-emitting diodes with dimension control and ligand engineering. *ACS Nano* **18**, 8557–8570 (2024).

20. I. L. K. Sivakumar, V. B. Shetty, S. Paramasivam, M. K. Rao, S. Kumar, S. S. Kumar, A review on perovskites based nanocrystals as potential ECL emitter: Challenges and future opportunities. *J. Mater. Chem. C* **12**, 10390–10407 (2024).

21. Y. Gao, Y. Liu, F. Zhang, X. Bao, Z. Xu, X. Bai, M. Lu, Y. Wu, Z. Wu, Y. Zhang, High-performance perovskite light-emitting diodes enabled by passivating defect and constructing dual energy-transfer pathway through functional perovskite nanocrystals. *Adv. Mater.* **34**, e2207445 (2022).

22. Y. K. Wang, F. Yuan, Y. Dong, J. Y. Li, A. Johnston, B. Chen, M. I. Saidaminov, C. Zhou, X. Zheng, Y. Hou, All-inorganic quantum-dot LEDs based on a phase-stabilized α-$CsPbI_3$ perovskite. *Angew. Chem. Int. Edit.* **60**, 16164–16170 (2021).

23. Y. Dong, Y.-K. Wang, F. Yuan, A. Johnston, Y. Liu, D. Ma, M.-J. Choi, B. Chen, M. Chekini, S.-W. Baek, Bipolar-shell resurfacing for blue LEDs based on strongly confined perovskite quantum dots. *Nat. Nanotechnol.* **15**, 668–674 (2020).

24. Y. Feng, H. Li, M. Zhu, Y. Gao, Q. Cai, G. Lu, X. Dai, Z. Ye, H. He, Nucleophilic reaction-enabled chloride modification on $CsPbI_3$ quantum dots for pure red light-emitting diodes with efficiency exceeding 26%. *Angew. Chem. Int. Edit.* **63**, e202318777 (2024).

25. J. Zeng, X. Sun, Y. Liu, W. Jin, S. He, X. Zhu, K. Niu, G. Sun, J. Li, H. He, Switchable interfacial reaction enables bright and stable deep-red perovskite light-emitting diodes. *Nat. Photon.* **18**, 325–333 (2024)

26. H. Li, Y. Feng, M. Zhu, Y. Gao, C. Fan, Q. Cui, Q. Cai, K. Yang, H. He, X. Dai, J. Huang, Z. Ye, Nanosurface-reconstructed perovskite for highly efficient and stable active-matrix light-emitting diode display. *Nat. Nanotechnol.* **19***,* 638–645 (2024).

27. S. Sun, M. Lu, P. Lu, X. Li, F. Zhang, Z. Wu, T. Wang, F. Yan, T. Li, T. Feng, Modulation of nucleation and growth kinetics of perovskite nanocrystals enables efficient and spectrally stable pure-red light-emitting diodes. *Nano Lett.* **24**, 5631–5638 (2024)

28. Y.-K. Wang, H. Wan, S. Teale, L. Grater, F. Zhao, Z. Zhang, H.-W. Duan, M. Imran, S.-D. Wang, S. Hoogland, L.-S. Liao, Long-range order enabled stability in quantum dot light-emitting diodes. *Nature* **629**, 586–591 (2024)

29. B. Lyu, H. Lin, D. Li, A. Sergeev, Q. Wang, Z. Jiang, L. Huo, H. Su, K. S. Wong, Y. Wang, Side-chain-promoted polymer architecture enabling stable mixed-halide perovskite light-emitting diodes. *ACS Energy Lett.* **9**, 2118–2127 (2024).

30. D. J. Norris, M. Bawendi, Measurement and assignment of the size-dependent optical spectrum in CdSe quantum dots. *Phys. Rev. B* **53**, 16338–16346 (1996).

31. S. Baskoutas, A. F. Terzis, Size-dependent band gap of colloidal quantum dots. *J. Appl. Phys.* **99**, 013708 (2006).

32. R. E. Bailey, S. Nie, Alloyed semiconductor quantum dots: Tuning the optical properties without changing the particle size. *J. Am. Chem. Soc.* **125**, 7100–7106 (2003)..

33. J. Jiang, Z. Chu, Z. Yin, J. Li, Y. Yang, J. Chen, J. Wu, J. You, X. Zhang, Red perovskite light-emitting diodes with efficiency exceeding 25% realized by co-spacer cations. *Adv. Mater.* **34**, e2204460 (2022).

34. Y. K. Wang, K. Singh, J. Y. Li, Y. Dong, X. Q. Wang, J. M. Pina, Y. J. Yu, R. Sabatini, Y. Liu, D. Ma, In-situ inorganic ligand replenishment enables bandgap stability in mixed-halide perovskite quantum dot solids. *Adv. Mater.* **34**, e2200854 (2022).

35. K. Wang, Z.-Y. Lin, Z. Zhang, L. Jin, K. Ma, A. H. Coffey, H. R. Atapattu, Y. Gao, J. Y. Park, Z. Wei, Suppressing phase disproportionation in quasi-2D perovskite light-emitting diodes. *Nat. Commun.* **14**, 397 (2023).

36. D. Ma, K. Lin, Y. Dong, H. Choubisa, A. H. Proppe, D. Wu, Y.-K. Wang, B. Chen, P. Li, J. Z. Fan, Distribution control enables efficient reduced-dimensional perovskite LEDs. *Nature* **599**, 594–598 (2021).

37. S. Wei, Y. Yang, X. Kang, L. Wang, L. Huang, D. Pan, Room-temperature and gram-scale synthesis of $CsPbX_3$ (X= Cl, Br, I) perovskite nanocrystals with 50–85% photoluminescence quantum yields. *Chem. Commun.* **52**, 7265–7268 (2016).

38. Q. A. Akkerman, V. D'Innocenzo, S. Accornero, A. Scarpellini, A. Petrozza, M. Prato, L. Manna, Tuning the optical properties of cesium lead halide perovskite nanocrystals by anion exchange reactions. *J. Am. Chem. Soc.* **137**, 10276–10281 (2015).

39. H. Zhang, X. Fu, Y. Tang, H. Wang, C. Zhang, W. W. Yu, X. Wang, Y. Zhang, M. Xiao, Phase segregation due to ion migration in all-inorganic mixed-halide perovskite nanocrystals. *Nat. Commun.* **10**, 1–8 (2019).

40. A. J. Knight, L. M. Herz, Preventing phase segregation in mixed-halide perovskites: A perspective. *Energ. Environ. Sci.* **13**, 2024–2046 (2020).

41. S. J. Yang, K. Wang, Y. Luo, J. Y. Park, H. Yang, A. H. Coffey, K. Ma, J. Sun, S. Wieghold, C. Zhu, Two-factor phase separations in mixed-halide quasi-2D perovskite LEDs: Dimensionality and halide segregations. *ACS Energy Lett.* **8**, 3693–3701 (2023).

42. W. Jin, Y. Deng, B. Guo, Y. Lian, B. Zhao, D. Di, X. Sun, K. Wang, S. Chen, Y. Yang, On the accurate characterization of quantum-dot light-emitting diodes for display applications. *NPJ Flex. Electron.* **6**, 35 (2022).

43. S. Coe-Sullivan, Quantum dot developments. *Nat. Photonics* **3**, 315–316 (2009).

44. Y. Shirasaki, G. J. Supran, M. G. Bawendi, V. Bulović, Emergence of colloidal quantum-dot light-emitting technologies. *Nat. Photonics* **7**, 13–23 (2013).

45. M. Yuan, L. N. Quan, R. Comin, G. Walters, R. Sabatini, O. Voznyy, S. Hoogland, Y. Zhao, E. M. Beauregard, P. Kanjanaboos, Perovskite energy funnels for efficient light-emitting diodes. *Nat. Nanotechnol.* **11**, 872–877 (2016).

46. M. G. Greiner, A. Singldinger, N. A. Henke, C. Lampe, U. Leo, M. Gramlich, A. S. Urban, Energy transfer in stability-optimized perovskite nanocrystals. *Nano Lett.* **22**, 6709–6715 (2022).

47. P. Chen, Y. Meng, M. Ahmadi, Q. Peng, C. Gao, L. Xu, M. Shao, Z. Xiong, B. Hu, Charge-transfer versus energy-transfer in quasi-2D perovskite light-emitting diodes. *Nano Energy* **50**, 615–622 (2018).

48. J. Ye, Z. Li, D. J. Kubicki, Y. Zhang, L. Dai, C. Otero-Martínez, M. A. Reus, R. Arul, K. R. Dudipala, Z. Andaji-Garmaroudi, Elucidating the role of antisolvents on the surface chemistry and optoelectronic properties of $CsPbBr_xI_{3-x}$ perovskite nanocrystals. *J. Am. Chem. Soc.* **144**, 12102–12115 (2022).

49. C. Zou, L. Y. Lin, Effect of emitter orientation on the outcoupling efficiency of perovskite light-emitting diodes. *Opt. Lett.* **45**, 4786–4789 (2020)..

50. Y. Jiang, J. Wei, M. Yuan, Energy-funneling process in quasi-2D perovskite light-emitting diodes. *J. Phys. Chem. Lett.* **12**, 2593–2606 (2021).

51. L. Lei, D. Seyitliyev, S. Stuard, J. Mendes, Q. Dong, X. Y. Fu, Y. A. Chen, S. L. He, X. P. Yi, L. P. Zhu, C. H. Chang, H. Ade, K. Gundogdu, F. So, Efficient energy funneling in quasi-2D perovskites: From light emission to lasing. *Adv. Mater.* **32**, e1906571 (2020).

52. B. Zhao, S. Bai, V. Kim, R. Lamboll, R. Shivanna, F. Auras, J. M. Richter, L. Yang, L. Dai, M. Alsari, High-efficiency perovskite–polymer bulk heterostructure light-emitting diodes. *Nat. Photonics* **12**, 783–789 (2018).

53. L. Dai, Z. Deng, F. Auras, H. Goodwin, Z. Zhang, J. C. Walmsley, P. D. Bristowe, F. Deschler, N. C. Greenham, Slow carrier relaxation in tin-based perovskite nanocrystals. *Nat. Photonics* **15**, 696–702 (2021).

54. M. Xu, Q. Peng, W. Zou, L. Gu, L. Xu, L. Cheng, Y. He, M. Yang, N. Wang, W. Huang, A transient-electroluminescence study on perovskite light-emitting diodes. *Appl. Phys. Lett.* **115**, 041102 (2019).

55. Z. Liu, W. Qiu, X. Peng, G. Sun, X. Liu, D. Liu, Z. Li, F. He, C. Shen, Q. Gu, Perovskite light-emitting diodes with EQE exceeding 28% through a synergetic dual-additive strategy for defect passivation and nanostructure regulation. *Adv. Mater.* **33**, e2103268 (2021).

56. C. Sun, Y. Jiang, M. Cui, L. Qiao, J. Wei, Y. Huang, L. Zhang, T. He, S. Li, H.-Y. Hsu, High-performance large-area quasi-2D perovskite light-emitting diodes. *Nat. Commun.* **12**, 1–11 (2021).

57. P. Giannozzi, S. Baroni, N. Bonini, M. Calandra, R. Car, C. Cavazzoni, D. Ceresoli, G. L. Chiarotti, M. Cococcioni, I. Dabo, QUANTUM ESPRESSO: A modular and open-source software project for quantum simulations of materials. *J. Phys. Condens. Mat.* **21**, 395502 (2009).

58. P. Giannozzi, O. Andreussi, T. Brumme, O. Bunau, M. B. Nardelli, M. Calandra, R. Car, C. Cavazzoni, D. Ceresoli, M. Cococcioni, Advanced capabilities for materials modelling with Quantum ESPRESSO. *J. Phys. Condens. Mat.* **29**, 465901 (2017).

59. J. P. Perdew, A. Ruzsinszky, G. I. Csonka, O. A. Vydrov, G. E. Scuseria, L. A. Constantin, X. Zhou, K. Burke, Restoring the density-gradient expansion for exchange in solids and surfaces. *Phys. Rev. Lett.* **100**, 136406 (2008).

60. K. F. Garrity, J. W. Bennett, K. M. Rabe, D. Vanderbilt, Pseudopotentials for high-throughput DFT calculations. *Comp. Mater. Sci.* **81**, 446–452 (2014).

61. S. Grimme, J. Antony, S. Ehrlich, H. Krieg, A consistent and accurate ab initio parametrization of density functional dispersion correction (DFT-D) for the 94 elements H-Pu. *J. Chem. Phys.* **132**, 154104 (2010).

62. H. J. Monkhorst, J. D. Pack, Special points for Brillouin-zone integrations. *Phys. Rev. B* **13**, 5188–5192 (1976).

63. S. Maheshwari, T. J. Savenije, N. Renaud, F. C. Grozema, Computational design of two-dimensional perovskites with functional organic cations. *J. Phys. Chem. C* **122**, 17118–17122 (2018).

64. D. Ghosh, D. Acharya, L. Pedesseau, C. Katan, J. Even, S. Tretiak, A. J. Neukirch, Charge carrier dynamics in two-dimensional hybrid perovskites: Dion–Jacobson vs. Ruddlesden–Popper phases. *J. Mater. Chem. A* **8**, 22009–22022 (2020).

65. A. Bala, A. K. Deb, V. Kumar, Atomic and electronic structure of two-dimensional inorganic halide perovskites $A_{n+1}M_nX_{3n+1}$ (n= 1–6, A= Cs, M= Pb and Sn, and X= Cl, Br, and I) from ab initio calculations. *J. Phys. Chem. C* **122**, 7464–7473 (2018).

66. B. Traoré, J. Even, L. Pedesseau, M. Kepenekian, C. Katan, Band gap, effective masses, and energy level alignment of 2D and 3D halide perovskites and heterostructures using DFT-1/2. *Phys. Rev. Mater.* **6**, 014604 (2022).

67. Y. Marcus, *Properties of Solvents* (Wiley, 1998).

68. M. Anaya, B. P. Rand, R. J. Holmes, D. Credgington, H. J. Bolink, R. H. Friend, J. Wang, N. C. Greenham, S. D. Stranks, Best practices for measuring emerging light-emitting diode technologies. *Nat. Photonics* **13**, 818–821 (2019).

69. H. Cho, S.-H. Jeong, M.-H. Park, Y.-H. Kim, C. Wolf, C.-L. Lee, J. H. Heo, A. Sadhanala, N. Myoung, S. Yoo, S. H. Im, R. H. Friend, T.-W. Lee, Overcoming the electroluminescence efficiency limitations of perovskite light-emitting diodes. *Science* **350**, 1222–1225 (2015).

70. Y. Shen, L. P. Cheng, Y. Q. Li, W. Li, J. D. Chen, S. T. Lee, J. X. Tang, High-efficiency perovskite light-emitting diodes with synergetic outcoupling enhancement. *Adv. Mater.* **31**, e1901517 (2019).

71. Y. Jiang, M. Cui, S. Li, C. Sun, Y. Huang, J. Wei, L. Zhang, M. Lv, C. Qin, Y. Liu, Reducing the impact of Auger recombination in quasi-2D perovskite light-emitting diodes. *Nat. Commun.* **12**, 336 (2021).

72. W. Dong, X. Zhang, F. Yang, Q. Zeng, W. Yin, W. Zhang, H. Wang, X. Yang, S. V. Kershaw, B. Yang, Amine-terminated carbon dots linking hole transport layer and vertically oriented quasi-2D perovskites through hydrogen bonds enable efficient LEDs. *ACS Nano* **16**, 9679–9690 (2022).

73. Y. Zhao, W. Feng, M. Li, J. Lu, X. Qin, K. Lin, J. Luo, W.-H. Zhang, E. L. Lim, Z. Wei, Efficient perovskite light-emitting diodes with chemically bonded contact and regulated charge behavior. *Nano Lett.* **23**, 8560–8567 (2023).

74. Q. Wan, W. Zheng, C. Zou, F. Carulli, C. Zhang, H. Song, M. Liu, Q. Zhang, L. Y. Lin, L. Kong, Ultrathin light-emitting diodes with external efficiency over 26% based on resurfaced perovskite nanocrystals. *ACS Energy Lett.* **8**, 927–934 (2023).

75. D. Zhang, Y. Fu, W. Wu, B. Li, H. Zhu, H. Zhan, Y. Cheng, C. Qin, L. Wang, Comprehensive passivation for high-performance quasi-2D perovskite LEDs. *Small* **19**, e2206927 (2023).

76. W. Yu, M. Wei, Z. Tang, H. Zou, L. Li, Y. Zou, S. Yang, Y. Wang, Y. Zhang, X. Li, Separating crystal growth from nucleation enables the in situ controllable synthesis of nanocrystals for efficient perovskite light-emitting diodes. *Adv. Mater.* **35**, 2301114 (2023).

77. C. Peng, R. Zhang, H. Chen, Y. Liu, S. Zhang, T. Fang, R. Guo, J. Zhang, Q. Shan, Y. Jin, A demulsification–crystallization model for high-quality perovskite nanocrystals. *Adv. Mater.* **35**, e2206969 (2023).

78. Z. Li, Z. Chen, Z. Shi, G. Zou, L. Chu, X.-K. Chen, C. Zhang, S. K. So, H.-L. Yip, Charge injection engineering at organic/inorganic heterointerfaces for high-efficiency and fast-response perovskite light-emitting diodes. *Nat. Commun.* **14**, 6441 (2023).

79. L. Kong, Y. Luo, L. Turyanska, T. Zhang, Z. Zhang, G. Xing, Y. Yang, C. Zhang, X. Yang, A spacer cation assisted nucleation and growth strategy enables efficient and high-luminance quasi-2D perovskite LEDs. *Adv. Funct. Mater.* **33**, 2209186 (2023).

80. Y. B. Cao, D. Zhang, Q. Zhang, X. Qiu, Y. Zhou, S. Poddar, Y. Fu, Y. Zhu, J.-F. Liao, L. Shu, High-efficiency, flexible and large-area red/green/blue all-inorganic metal halide perovskite quantum wires-based light-emitting diodes. *Nat. Commun.* **14**, 4611 (2023).

81. S. Ding, Q. Wang, W. Gu, Z. Tang, B. Zhang, C. Wu, X. Zhang, H. Chen, X. Zhang, R. Cao, Phase dimensions resolving of efficient and stable perovskite light-emitting diodes at high brightness. *Nat. Photon.* **18**, 363–370 (2024).

82. D. Zhang, C. Liu, J. Sun, Q. Xiong, X. Xiao, D. Li, B. Lyu, H. Su, W. C. Choy, Self-stabilized quasi-2D perovskite with an ion-migration-inhibition ligand for pure green LEDs. *ACS Energy Lett.* **9**, 1133–1140 (2024).

83. Q. Zhang, Y. Zhao, X. Qin, M. Li, H. Sun, P. Zhou, W. Feng, Y. Li, J. Lu, K. Lin, Efficient perovskite light-emitting diodes enabled by nickel acetate interlayer. *Adv. Funct. Mater.* **34**, 2308547 (2024).

84. S. Xing, Y. Yuan, G. Zhang, S. Zhang, Y. Lian, W. Tang, K. Zhou, S. Liu, Y. Gao, Z. Ren, Energy-efficient perovskite LEDs with Rec. 2020 compliance. *ACS Energy Lett.* **9**, 3643–3651 (2024).

85. S. Q. Sun, J. W. Tai, W. He, Y. J. Yu, Z. Q. Feng, Q. Sun, K. N. Tong, K. Shi, B. C. Liu, M. Zhu, Enhancing light outcoupling efficiency via anisotropic low refractive index electron transporting materials for efficient perovskite light-emitting diodes. *Adv. Mater.* **36**, e2400421 (2024).

86. H.-D. Lee, S.-J. Woo, S. Kim, J. Kim, H. Zhou, S. J. Han, K. Y. Jang, D.-H. Kim, J. Park, S. Yoo, T.-W. Lee, Valley-centre tandem perovskite light-emitting diodes. *Nat. Nanotechnol.* **19**, 624–631 (2024).

87. Y. Ke, J. Guo, D. Kong, J. Wang, G. Kusch, C. Lin, D. Liu, Z. Kuang, D. Qian, F. Zhou, Efficient and bright deep-red light-emitting diodes based on a lateral 0D/3D perovskite heterostructure. *Adv. Mater.* **36**, 2207301 (2024).

88. P. Lu, M. Lu, F. Zhang, F. Qin, S. Sun, Y. Zhang, W. Y. William, X. Bai, Bright and spectrally stable pure-red $CsPb(Br/I)_3$ quantum dot LEDs realized by synchronous device structure and ligand engineering. *Nano Energy* **108**, 108208 (2023).

89. H. W. Duan, F. Zhao, S. N. Li, J. L. Pan, W. S. Shen, S. M. Li, Q. Zhang, Y. K. Wang, L. S. Liao, Bi-ligand synergy enables threshold low voltage and bandgap stable pure-red mix-halide perovskite LEDs. *Adv. Funct. Mater.* **34**, 2310697 (2024).

90. L. Kong, Y. Sun, B. Zhao, K. Ji, J. Feng, J. Dong, Y. Wang, Z. Liu, S. Maqbool, Y. Li, Y. Yang, L. Dai, W. Lee, C. Cho, S. D. Stranks, R. H. Friend, N. Wang, N. C. Greenham, X. Yang, Fabrication of red-emitting perovskite LEDs by stabilizing their octahedral structure. *Nature* **631**, 73–79 (2024).

91. A. Liang, K. Wang, Y. Gao, B. P. Finkenauer, C. Zhu, L. Jin, L. Huang, L. Dou, Highly efficient halide perovskite light-emitting diodes via molecular passivation. *Chem. Int. Edit.* **60**, 8337–8343 (2021).

92. L. Zhao, K. Roh, S. Kacmoli, K. Al Kurdi, S. Jhulki, S. Barlow, S. R. Marder, C. Gmachl, B. P. Rand, Thermal management enables bright and stable perovskite light-emitting diodes. *Adv. Mater.* **32**, e2000752 (2020).

93. W. Xu, Q. Hu, S. Bai, C. Bao, Y. Miao, Z. Yuan, T. Borzda, A. J. Barker, E. Tyukalova, Z. Hu, Rational molecular passivation for high-performance perovskite light-emitting diodes. *Nat. Photonics* **13**, 418–424 (2019).

94. L. Zhu, H. Cao, C. Xue, H. Zhang, M. Qin, J. Wang, K. Wen, Z. Fu, T. Jiang, L. Xu, Unveiling the additive-assisted oriented growth of perovskite crystallite for high performance light-emitting diodes. *Nat. Commun.* **12**, 5081 (2021).

95. Y. Sun, L. Ge, L. Dai, C. Cho, J. Ferrer Orri, K. Ji, S. J. Zelewski, Y. Liu, A. J. Mirabelli, Y. Zhang, J.-Y. Huang, Y. Wang, K. Gong, M. C. Lai, L. Zhang, D. Yang, J. Lin, E. M. Tennyson, C. Ducati, S. D. Stranks, L.-S. Cui, N. C. Greenham, Bright and stable perovskite light-emitting diodes in the near-infrared range. *Nature* **615**, 830–835 (2023).

96. Z. Li, Z. Ren, Q. Liang, P. W. Fong, J. Tian, G. Li, Eliminating the adverse impact of composition modulation in perovskite light-emitting diodes toward ultra-high brightness and stability. *Adv. Mater.* **36**, e2313981 (2024).

97. F. Yuan, G. Folpini, T. Liu, U. Singh, A. Treglia, J. W. M. Lim, J. Klarbring, S. I. Simak, I. A. Abrikosov, T. C. Sum, A. Petrozza, F. Gao, Bright and stable near-infrared lead-free perovskite light-emitting diodes. *Nat. Photonics* **18**, 170–176 (2024).

98. M. Li, Y. Yang, Z. Kuang, C. Hao, S. Wang, F. Lu, Z. Liu, J. Liu, L. Zeng, Y. Cai, Y. Mao, J. Guo, H. Tian, G. Xing, Y. Cao, C. Ma, N. Wang, Q. Peng, L. Zhu, W. Huang, J. Wang, Acceleration of radiative recombination for efficient perovskite LEDs. *Nature* **630**, 631–635 (2024).

99. Y. Tong, E. Bladt, M. F. Aygüler, A. Manzi, K. Z. Milowska, V. A. Hintermayr, P. Docampo, S. Bals, A. S. Urban, L. Polavarapu, Highly luminescent cesium lead halide perovskite nanocrystals with tunable composition and thickness by ultrasonication. *Angew. Chem. Int. Edit.* **55**, 13887–13892 (2016).

100. Z.-Y. Zhu, Q.-Q. Yang, L.-F. Gao, L. Zhang, A.-Y. Shi, C.-L. Sun, Q. Wang, H.-L. Zhang, Solvent-free mechanosynthesis of composition-tunable cesium lead halide perovskite quantum dots. *J. Phys. Chem. Lett.* **8**, 1610–1614 (2017).

101. Q. Pan, H. Hu, Y. Zou, M. Chen, L. Wu, D. Yang, X. Yuan, J. Fan, B. Sun, Q. Zhang, Microwave-assisted synthesis of high-quality “all-inorganic” $CsPbX_3$ (X= Cl, Br, I) perovskite

nanocrystals and their application in light emitting diodes. *J. Mater. Chem. C* **5**, 10947–10954 (2017).

102. L. Protesescu, S. Yakunin, M. I. Bodnarchuk, F. Krieg, R. Caputo, C. H. Hendon, R. X. Yang, A. Walsh, M. V. Kovalenko, Nanocrystals of cesium lead halide perovskites ($CsPbX_3$, X = Cl, Br, and I): Novel optoelectronic materials showing bright emission with wide color gamut. *Nano Lett.* **15**, 3692–3696 (2015).

103. M. C. Weidman, M. Seitz, S. D. Stranks, W. A. Tisdale, Highly tunable colloidal perovskite nanoplatelets through variable cation, metal, and halide composition. *ACS Nano* **10**, 7830–7839 (2016).

104. K.-K. Liu, Q. Liu, D.-W. Yang, Y.-C. Liang, L.-Z. Sui, J.-Y. Wei, G.-W. Xue, W.-B. Zhao, X.-Y. Wu, L. Dong, Water-induced $MAPbBr_3$@PbBr(OH) with enhanced luminescence and stability. *Light-Sci. Appl.* **9**, 44 (2020).

105. Y. Li, H. Huang, Y. Xiong, A. F. Richter, S. V. Kershaw, J. Feldmann, A. L. Rogach, Using polar alcohols for the direct synthesis of cesium lead halide perovskite nanorods with anisotropic emission. *ACS Nano* **13**, 8237–8245 (2019).

106. B. Zhou, D. Ding, Y. Wang, S. Fang, Z. Liu, J. Tang, H. Li, H. Zhong, B. Tian, Y. Shi, A scalable H_2O–DMF–DMSO solvent synthesis of highly luminescent inorganic perovskite-related cesium lead bromides. *Adv. Opt. Mater.* **9**, 2001435 (2021).

107. F. Fang, W. Chen, Y. Li, H. Liu, M. Mei, R. Zhang, J. Hao, M. Mikita, W. Cao, R. Pan, Employing polar solvent controlled ionization in precursors for synthesis of high-quality inorganic perovskite nanocrystals at room temperature. *Adv. Funct. Mater.* **28**, 1706000 (2018).

108. X. Zhang, X. Bai, H. Wu, X. Zhang, C. Sun, Y. Zhang, W. Zhang, W. Zheng, W. W. Yu, A. L. Rogach, Water-assisted size and shape control of $CsPbBr_3$ perovskite nanocrystals. *Angew. Chem. Int. Edit.* **57**, 3337–3342 (2018).
